# Essential Nutrients and White Matter Hyperintensities: A Two-Sample Mendelian Randomization Study

**DOI:** 10.3390/biomedicines12040810

**Published:** 2024-04-06

**Authors:** Zhengrui Wang, Kailin Xia, Jiayi Li, Yanru Liu, Yumou Zhou, Linjing Zhang, Lu Tang, Xiangzhu Zeng, Dongsheng Fan, Qiong Yang

**Affiliations:** 1 Department of Neurology, Peking University Third Hospital, Beijing 100191, China; 2 Peking University Health Science Center, Beijing 100191, China; 3 Department of Radiology, Peking University Third Hospital, Beijing 100191, China; 4 Beijing Key Laboratory of Biomarker and Translational Research in Neurodegenerative Diseases, Beijing 100191, China; 5 Key Laboratory for Neuroscience, National Health Commission, Ministry of Education, Peking University, Beijing 100191, China

**Keywords:** white matter hyperintensities, Mendelian randomization, genes, risk factors

## Abstract

Stroke and dementia have been linked to the appearance of white matter hyperintensities (WMHs). Meanwhile, diffusion tensor imaging (DTI) might capture the microstructural change in white matter early. Specific dietary interventions may help to reduce the risk of WMHs. However, research on the relationship between specific nutrients and white matter changes is still lacking. We aimed to investigate the causal effects of essential nutrients (amino acids, fatty acids, mineral elements, and vitamins) on WMHs and DTI measures, including fraction anisotropy (FA) and mean diffusivity (MD), by a Mendelian randomization analysis. We selected single nucleotide polymorphisms (SNPs) associated with each nutrient as instrumental variables to assess the causal effects of nutrient-related exposures on WMHs, FA, and MD. The outcome was from a recently published large-scale European Genome Wide Association Studies pooled dataset, including WMHs (N = 18,381), FA (N = 17,663), and MD (N = 17,467) data. We used the inverse variance weighting (IVW) method as the primary method, and sensitivity analyses were conducted using the simple median, weighted median, and MR-Egger methods. Genetically predicted serum calcium level was positively associated with WMHs risk, with an 8.1% increase in WMHs risk per standard deviation unit increase in calcium concentration (OR = 1.081, 95% CI = 1.006–1.161, *p* = 0.035). The plasma linoleic acid level was negatively associated with FA (OR = 0.776, 95% CI = 0.616–0.978, *p* = 0.032). Our study demonstrated that genetically predicted calcium was a potential risk factor for WMHs, and linoleic acid may be negatively associated with FA, providing evidence for interventions from the perspective of gene-environment interactions.

## 1. Introduction

White matter hyperintensities (WMHs) are defined as periventricular and subcortical (semi-ovoid centers) low-density bands on CT or high signal areas on magnetic resonance imaging T2-weighted images, showing patchy or diffuse patchy lesions [1]. WMHs increase with age and are considered as markers of cerebral small vessel diseases and are associated with the increased risk of stroke and dementia [2]. Although their pathogenesis is uncertain, WMHs are usually thought to result from chronic cerebral hypoperfusion, altered vascular permeability, blood–brain barrier dysfunction, and inflammation reaction [3,4,5,6].

Diffusion tensor imaging (DTI) is a quantitative MRI technique that measures the movement of water within the tissue microstructure [7,8]. DTI measures white matter changes both in areas of WMHs and in normal appearing white matter, which indicates that DTI might be more sensitive than WMHs and might be a biomarker to monitor the progression of white matter changes. Two DTI measures that are commonly used to provide information about the white matter microstructure are fractional anisotropy (FA) and mean diffusivity (MD). FA measures the direction of diffusion to reflect the integrity of white matter bundles, and MD measures the diffusion of water molecules to reflect diffuse white matter injury [8]. A lower FA and higher MD reflect lower microstructural connectivity and capture early damage to white matter and are more useful in predicting diseases such as dementia than WMHs [2,9,10].

Essential nutrients, including vitamins, amino acids, fatty acids, and minerals, can be obtained from the diet. Previous studies showed that some nutrients, e.g., vitamins, minerals, and ω3 polyunsaturated fatty acids contained in the diet that is rich in vegetables, fruits, nuts, cereals and fish, were associated with the decreased risk of brain aging, cardiovascular diseases, and cognitive impairment [11]. Given that stroke and cognitive impairment are also related to white matter changes, specific diet or nutrients are assumed to play a role in white matter changes, which was supported by a few studies [12,13,14,15,16] while other studies presented inconsistent results [14,16]. The different methodologies, such as nutrient measurement and the duration of observation and intervention, could partially explain the inconsistency and demonstrate the challenges in studies focused on nutrients. Randomized controlled trials (RCTs) have been recognized to overcome the limitations of observational studies and to provide the highest level of evidence [17], but there have been no previous RCTs to determine the effects of essential nutrients on WMHs. Mendelian randomization (MR) analysis can evaluate the causal inference of modifiable factors on disease risk based on genetic data. MR analysis uses single nucleotide polymorphisms (SNPs) as instrumental variables (IVs), mimicking random assignment by naturally assigning alleles, which are less susceptible to confounding bias or reverse causation [18].

In this study, we used a two-sample MR approach to investigate whether genetically predicted levels of essential nutrients including amino acids, fatty acids, minerals, and vitamins are associated with WMHs and two DTI measures, FA and MD.

## 2. Materials and Methods

### 2.1. Exposure and Outcome Data

Essential nutrients of several types, namely, amino acids, fatty acids, minerals, and vitamins, were chosen as exposures. Exposure-related SNPs were obtained from the largest genome-wide association studies (GWASs) in European populations that were published most recently and available from PubMed [19,20,21,22,23,24,25,26,27,28,29,30,31,32,33,34]. Amino acid-related SNPs were extracted from isoleucine, leucine, lysine, methionine, phenylalanine, tryptophan, and valine datasets [19,20]. The essential fatty acids considered were ω3 polyunsaturated fatty acids including docosapentaenoic acid (DPA), docosahexaenoic acid (DHA), alpha-linolenic acid (ALA), and eicosapentaenoic acid (EPA) and ω6 polyunsaturated fatty acids including arachidonic acid (AA), dihomogamma-linolenic acid (DGLA), gamma-linolenic acid (GLA), and linoleic acid (LA) [19,21,22]. The chosen essential minerals were calcium [23], copper, iron [24], magnesium [25], phosphorus [26], and zinc [27]. The absolute and relative levels of vitamins or vitaminogens in blood were examined to perform a comprehensive assessment of the impact of vitamins and pro-vitamins on disease. The absolute concentrations of vitamins or pro-vitamins, namely, absolute vitamin A (retinol) [28], absolute β-carotene [29], absolute vitamin B-6 [30], absolute vitamin C (ascorbic acid) [31], relative vitamin C (ascorbic acid) [19], absolute vitamin D [25-hydroxyvitamin D (25OHD)] [32], absolute vitamin E (α-tocopherol) [33], relative vitamin E (α-tocopherol and γ-tocopherol [19], and relative retinol [34], were analyzed.

Large-scale European GWAS summary data from a recently published study, which included WMHs (N = 18,381), FA (N = 17,663) and MD (N = 17,467) data, were considered as the outcome dataset [2].

### 2.2. Selection of Instrumental Variables (IVs)

In total, we referenced GWAS data for 32 different nutrient-related exposures. We removed nutrient-related exposures that only had one associated SNP or the associated SNPs cannot provide enough corresponding effects in the outcome. In the WMHs outcome analysis, we included 2 to 174 instrumental variables related to 21 nutrient-related exposures and removed 11 nutrient-related exposures: isoleucine, lysine, methionine, AA, ALA, DGLA, DPA, EPA, GLA, absolute beta-carotene, and absolute vitamin B-6. For the FA outcome, we included 2 to 170 IVs related to 25 nutrient-related exposures, and removed 7 nutrients: isoleucine, lysine, methionine, ALA, EPA, absolute beta-carotene, and absolute vitamin B-6. For the MD outcomes, we included 2 to 170 IVs related to 25 nutrient-related exposures, removing 7 nutrients: isoleucine, lysine, methionine, ALA, EPA, absolute beta-carotene, and absolute vitamin B-6.

We followed a strict set of criteria to select appropriate nutrient IVs, including (1) independent SNP loci (r^2^ = 0.01, KB = 5000) with *p* < 5 × 10^−8^ in the exposure GWAS that were selected as IVs that were significantly associated with the exposures; if no SNP with *p* < 5 × 10^−8^ are available, those with moderate significance level (*p* < 1 × 10^−5^) were used as proxies; (2) no rare SNPs were selected (MAF ≥ 0.01); and (3) loci with strong linkage disequilibrium (r^2^ > 0.8) with the original locus could be used as proxies. Due to methodological limitations, our analyses can only demonstrate exposures with at least 2 associated SNPs.

### 2.3. Mendelian Randomization

The main MR approach for analyzing causality was the multiplicative random-effects inverse variance-weighted (IVW) method. This method involves regressing the effect of SNPs on both the outcome and the exposure variables [35]. The simple median method, the weighted median method (only approved when ≥50% IVs are valid [36]), and the MR-Egger method (for the detection and correction of bias caused by pleiotropy [37]) were used as sensitivity analyses in order to guarantee that the IVW results are robust. In addition, we used the Cochran’s Q test for heterogeneity. Similar estimates for each IV indicated nonsignificant heterogeneity (*p* > 0.05). We used the MR-Egger method to test the horizontal pleiotropy by calculating the regression intercept. When the intercept was not significantly distant from the origin, it was considered to have no impact on multicollinearity. The “Leave one out” (LOO) method was used to assess the effect of individual SNP on the overall causality through individually excluding each genetic variant and recalculating MR estimates.

*p* values were adjusted according to the Bonferroni correction. A *p*-value < 0.05/k (k is the number of exposures) was considered significant, and a *p*-value between 0.05/k and 0.05 indicated a suggestive significant association.

**Figure 1:**
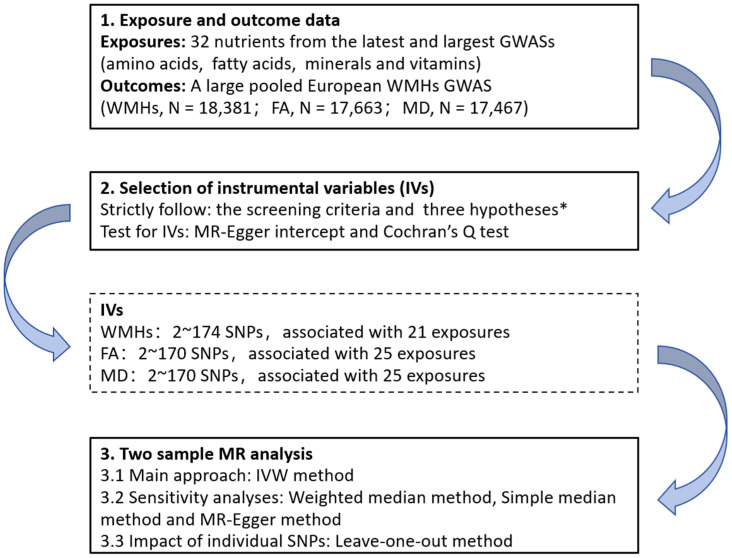
Flow chart of the Mendelian randomization analysis process in this study. * three key assumptions of Mendelian analyses: (1) the selected genetic variants are significantly associated with the risk of the outcome only through the exposure pathway; (2) the selected genetic variants should be significantly associated with the exposures; and (3) the selected genetic variants are not associated with other confounders. GWASs, genome-wide association studies; WMHs, white matter hyperintensities; FA, fractional order anisotropy; MD, mean diffusivity; IVs, instrumental variables; SNPs: single nucleotide polymorphisms; MR, Mendelian randomization; and IVW, inverse variance weighting.

R software, version 4.2.1 (R Core Team, Vienna, Austria), with the “TwoSampleMR” package (version 0.5.7), was used to perform all the analyses [38]. The flowchart of the analysis steps is shown in Figure 1.

## 3. Results

Of the 21 potential risk factors for WMHs from essential nutrients, 4 were related to amino acids, 2 were related to fatty acids, 6 were related to minerals, and 9 were related to vitamins. Of the 25 potential risk factors for FA from essential nutrients, 4 were related to amino acids, 6 were related to fatty acids, 6 were related to minerals, and 9 were related to vitamins. Of the 25 potential risk factors for MD from essential nutrients, 4 were related to amino acids, 6 were related to fatty acids, 6 were related to minerals, and 9 were related to vitamins.

### 3.1. Amino Acids

**Figure 2:**
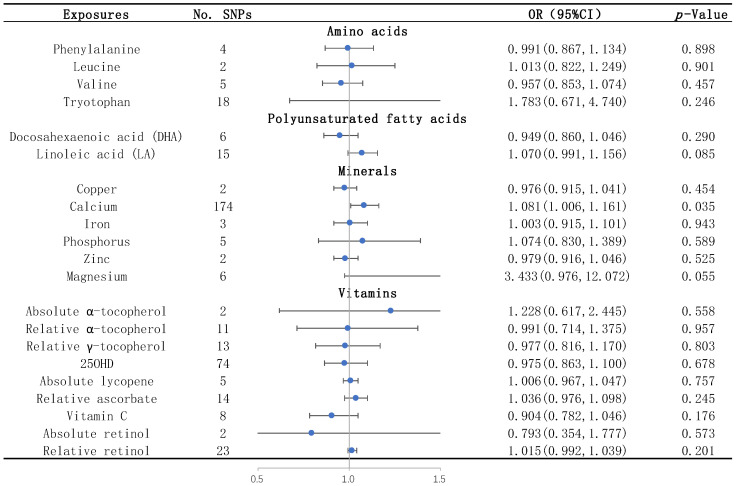
Causal effects of each nutrient on WMHs identified by IVW. The blue dots represent the OR value, and the straight line represents 95% CI. IVW, inverse variance weighting; SNPs, single nucleotide polymorphisms. OR, odds ratio; 95% CI, 95% confidence interval; and 25OHD: 25-hydroxyvitamin D.

None of the studied amino acids, including leucine, phenylalanine, tryptophan, and valine, were significantly associated with WMHs by the IVW method (Table 1 and Figure 2). None of them were significantly associated with the two DTI measures, FA and MD, by the IVW method (Table S2).

### 3.2. Unsaturated Fatty Acids

The results of the IVW analysis showed that the plasma level of linoleic acid was negatively associated with FA (OR = 0.776; 95% CI = 0.616–0.978, *p* = 0.032) (Table S2). Similar trends were shown by the sensitivity analyses including the simple median, the weighted median, and the MR–Egger method (Table S2). The IVs for linoleic acid had no heterogeneity or horizontal pleiotropy according to the Cochran’s Q test and the MR-Egger intercept test (Table S3). Through the LOO method, we found that the effect of linoleic acid on FA did not remain significant after removing some SNPs (rs99780, rs769449, rs821840, or rs7412) (Figure S4.2).

We also found that DGLA (OR = 0.727, 95% CI = 0.588–0.899, *p* = 0.003) and AA (OR = 1.081; 95% CI = 1.027–1.138, *p* = 0.003) were associated with FA by the IVW method (Table S2). However, sensitivity analyses and MR-Egger intercept tests were not performed, as there were only two available IVs for both DGLA and AA.

Regarding the other outcomes, none of the unsaturated fatty acids, including DGLA, DHA, DPA, GLA, AA and linoleic acid, showed effects on MD (Table S2), and neither DHA nor linoleic acid showed effects on WMHs after removing DGLA, DPA, GLA, and AA as described in the Materials and Methods Section (Table 1).

### 3.3. Mineral Elements

We found that the serum calcium level was a risk factor for WMHs, and the risk of WMHs was elevated by 8.1% for each standard deviation unit increase in calcium concentration (OR = 1.081; 95% CI = 1.006–1.161, *p* = 0.035) (Table 1 and Figure 2). The sensitivity analyses including the simple median, the weighted median, and the MR–Egger methods showed similar trends (Table 1). There was no heterogeneity or horizontal pleiotropy of calcium detected by the Cochran’s Q test and the MR-Egger intercept test (Table 2). 

Through the LOO method, we found that the effect of calcium on WMHs did not remain significant after removing some SNPs (rs1688131, rs6909201, rs760077, rs6841429, rs4917, or rs1260326) (Figure S2.13).

Regarding the other outcomes, FA and MD, none of the mineral elements, including copper, iron, zinc, and magnesium, showed effects (Table S2).

### 3.4. Vitamins

None of the vitamins, including absolute α-tocopherol, relative α-tocopherol, relative γ-tocopherol, 25OHD, absolute lycopene, relative ascorbate, vitamin C, absolute retinol, and relative retinol, were significantly associated with WMHs (Table 1 and Figure 2) and the two DTI measures, FA and MD (Table S2), by the IVW method.

## 4. Discussion

In our MR study, we analyzed the effects of essential nutrients on WMHs and two DTI measures, FA and MD, and showed that the serum calcium level was a potential risk factor for WMHs, and the plasma linoleic acid level was a potential risk factor for early damage to white matter as represented by FA.

Our findings suggested that the serum calcium level was a potential risk factor for WMHs. There were few previous studies on the relationship between calcium and WMHs, all of which were small cross-sectional studies; moreover, the conclusions of these studies were inconsistent. One study reported that the serum calcium level was not associated with white matter hyperintensities in older adults [39]. However, other studies have shown that the higher levels of serum calcium might be positively associated with the volume of cerebral white matter lesions in older adults, especially in men and in depressed patients [40]. Calcium and vitamin D intake [41] and the use of calcium-containing dietary supplements [42] might be positively associated with the volume of brain lesions (including those in both the gray and the white matter, albeit predominantly in the white matter) in older adults, which could be explained by our findings. The inconsistency might be interpreted by limitations of studies including the small sample size, the varied measurement methods of white matter changes, the lack of longitudinal data, etc.

Our findings suggested that the plasma level of linoleic acid, an ω-6 unsaturated fatty acid, was a potential risk factor for the early microstructural damage to white matter represented by FA. Previous studies on the relationship between linoleic acid and white matter changes and the underlying mechanism were lacking, probably because the level of linoleic acid is difficult to measure. However, it has been shown that linoleic acid enhances oxidative stress and TNF-alpha [43,44,45]. Therefore, linoleic acid might adversely affect the function and structure of white matter through inflammatory response mechanisms. Further studies are needed to clarify the relationship between the level of linoleic acid and white matter changes.

Previous studies showed that WMHs, as an imaging marker, was closely associated with stroke and dementia [46,47]. Regarding the relationship of calcium with stroke, several prospective studies have shown that serum calcium levels were related with the risk of ischemic stroke. In a cohort study enrolled about 440,000 adults, high serum calcium levels were associated with a significantly increased risk of both ischemic stroke and fatal ischemic stroke compared with low serum calcium levels [48]. Another cohort comprised 13,288 adults and showed a 16% increase in total stroke risk for every one SD increase in the serum calcium concentration [49]. However, an MR study showed that serum calcium concentration was not associated with the various subtypes of ischemic stroke, including large-artery stroke, cardiogenic embolism, and small-vessel stroke [50]. However, the statistical power for measuring calcium in that study was low because the SNPS explained only a small fraction (0.9%) of the variation in serum calcium levels, and thus, a weak association between genetically predicted serum calcium concentrations and ischemic stroke cannot be excluded. Regarding the relationship between calcium and dementia, population-based longitudinal studies showed that calcium supplementation may increase the risk of dementia and stroke-related dementia (including vascular and mixed dementia) in older women with cerebrovascular disease [46]. Higher serum calcium levels may increase the risk of Alzheimer’s disease in older adults [47]. However, an MR study also showed a trend of decreasing risk of Alzheimer’s disease with increasing serum calcium levels, but the results of this study were not statistically significant and other types of dementia including vascular and mixed dementia were not considered [51].

The mechanisms underlying the role calcium as a risk factor for WMHs remain unclear. One possible mechanism was that calcium may promote brain lesions via arterial calcification. Increases in dietary and serum calcium are associated with arterial calcification [41,52,53], while coronary and carotid calcification are independently associated with WMHs [54,55,56]. Second, disturbances in calcium metabolism may also be associated with hypertension and renal disease [40]. In addition, calcium may directly affect brain health by affecting neurotransmitter turnover and neurotoxicity mechanisms [41,42]. Thus, serum calcium may contribute to WMHs by either arterial calcification or another mechanism.

Our study presents the evidence of correlations between nutrients and WMHs and the early microstructural lesions of white matter represented by DTI parameters, FA and MD, providing the potential intervention targets for WMHs and its associated diseases. Our study utilized GWAS data with a large sample size and reliable sources. It overcame several challenges in conducting clinical studies on nutrients, such as the difficulty of accurately measuring nutrient concentrations and the demand for a long period to observe the effect of nutrients on outcomes. However, our study also had some limitations. First, our study investigated lifetime exposure to nutrients, and short-term dietary changes may not impact the outcomes. Second, genetically predicted calcium levels explain only a small fraction of the real calcium levels. Third, the disease may be heterogeneous, and calcium may only have an effect on a part of the population. Our study was performed in European population only and should be interpreted with caution when extrapolated to other populations with different dietary habits. Moreover, in GWASs for FA and MD, only the first principal components were used, which might affect the reliability of estimates and the directionality of the MR results [2].

## 5. Conclusions

In our study, we used a two-sample MR approach to analyze the effect of essential nutrients in blood including amino acids, fatty acids, minerals, and vitamins on white matter changes measured by WMHs and two DTI measures, FA and MD, suggesting that genetically predicted calcium was a potential risk factor for WMHs and that linoleic acid may be negatively associated with FA, which might provide the evidence for medical interventions in the general population from the perspective of gene–environment interactions. No association was found between other nutrients and white matter changes. These findings need to be verified by further clinical longitudinal studies.

## Figures and Tables

**Table 1:** Causal effects of each nutrient on WMHs identified by different MR methods.

Exposures	Methods	No. SNPs	OR(95%CI)	*p*-Value
Amino acids				
Phenylalanine	Simple median	4	0.972 (0.832, 1.136)	0.721
	Weighted median	4	0.991 (0.843, 1.166)	0.917
	MR Egger	4	1.053 (0.641, 1.730)	0.857
	IVW	4	0.991 (0.867, 1.134)	0.898
Leucine	IVW	2	1.013 (0.822, 1.249)	0.901
Valine	Simple median	5	1.094 (0.894, 1.338)	0.383
	Weighted median	5	0.893 (0.777, 1.025)	0.108
	MR Egger	5	0.608 (0.330, 1.119)	0.208
	IVW	5	0.957 (0.853, 1.074)	0.457
Tryptophan	Simple median	18	0.589 (0.164, 2.118)	0.417
	Weighted median	18	0.587 (0.173, 1.993)	0.393
	MR Egger	18	4060.115 (0.009, 1,924,702,705.747)	0.231
	IVW	18	1.783 (0.671, 4.740)	0.246
Polyunsaturated fatty acids				
Docosahexaenoic acid (DHA)	Simple median	6	0.914 (0.813, 1.026)	0.129
	Weighted median	6	0.927 (0.829, 1.037)	0.188
	MR Egger	6	1.380 (0.846, 2.249)	0.267
	IVW	6	0.949 (0.860, 1.046)	0.290
Linoleic acid (LA)	Simple median	15	1.064 (0.979, 1.155)	0.143
	Weighted median	15	1.045 (0.971, 1.125)	0.241
	MR Egger	15	1.125 (0.941, 1.345)	0.217
	IVW	15	1.070 (0.991, 1.156)	0.085
Minerals				
Copper	IVW	2	0.976 (0.915, 1.041)	0.454
Calcium	Simple median	174	1.070 (0.969, 1.182)	0.178
	Weighted median	174	1.058 (0.954, 1.172)	0.286
	MR Egger	174	1.119 (0.905, 1.383)	0.302
	IVW	174	1.081 (1.006, 1.161)	0.035
Iron	Simple median	3	1.047 (0.949, 1.153)	0.360
	Weighted median	3	1.043 (0.958, 1.137)	0.332
	MR Egger	3	1.223 (0.869, 1.723)	0.455
	IVW	3	1.003 (0.915, 1.101)	0.943
Phosphorus	Simple median	5	1.037 (0.731, 1.469)	0.840
	Weighted median	5	1.129 (0.834, 1.528)	0.433
	MR Egger	5	2.946 (0.754, 11.518)	0.218
	IVW	5	1.074 (0.830, 1.389)	0.589
Zinc	IVW	2	0.979 (0.916, 1.046)	0.525
Magnesium	Simple median	6	1.462 (0.216, 9.907)	0.697
	Weighted median	6	1.882 (0.389, 9.102)	0.432
	MR Egger	6	20.027 (0.540, 742.534)	0.179
	IVW	6	3.433 (0.976, 12.072)	0.055
Vitamins				
Absolute α-tocopherol	IVW	2	1.228 (0.617, 2.445)	0.558
Relative α-tocopherol	Simple median	11	0.886 (0.573, 1.369)	0.586
	Weighted median	11	1.041 (0.679, 1.596)	0.854
	MR Egger	11	1.091 (0.543, 2.193)	0.811
	IVW	11	0.991 (0.714, 1.375)	0.957
Relative γ-tocopherol	Simple median	13	0.923 (0.716, 1.190)	0.536
	Weighted median	13	1.014 (0.803, 1.281)	0.905
	MR Egger	13	1.150 (0.774, 1.708)	0.504
	IVW	13	0.977 (0.816, 1.170)	0.803
25OHD	Simple median	74	1.030 (0.869, 1.221)	0.736
	Weighted median	74	0.987 (0.851, 1.144)	0.862
	MR Egger	74	0.942 (0.784, 1.132)	0.525
	IVW	74	0.975 (0.863, 1.100)	0.678
Absolute lycopene	Simple median	5	0.988 (0.937, 1.043)	0.671
	Weighted median	5	1.002 (0.956, 1.051)	0.919
	MR Egger	5	1.008 (0.936, 1.085)	0.844
	IVW	5	1.006 (0.967, 1.047)	0.757
Relative ascorbate	Simple median	14	0.984 (0.893, 1.085)	0.751
	Weighted median	14	1.029 (0.946, 1.120)	0.508
	MR Egger	14	1.059 (0.954, 1.176)	0.304
	IVW	14	1.036 (0.976, 1.098)	0.245
Vitamin C	Simple median	8	0.899 (0.750, 1.078)	0.252
	Weighted median	8	0.875 (0.728, 1.052)	0.157
	MR Egger	8	0.866 (0.576, 1.302)	0.514
	IVW	8	0.904 (0.782, 1.046)	0.176
Absolute retinol	IVW	2	0.793 (0.354, 1.777)	0.573
Relative retinol	Simple median	23	1.013 (0.980, 1.046)	0.451
	Weighted median	23	1.012 (0.981, 1.044)	0.458
	MR Egger	23	0.987 (0.926, 1.052)	0.690
	IVW	23	1.015 (0.992, 1.039)	0.201

WMHs, white matter hyperintensities; MR, Mendelian randomization; SNPs, single nucleotide polymorphisms; OR, odds ratio; 95% CI, 95% confidence interval; IVW, inverse variance weighting; and 25OHD: 25-hydroxyvitamin D.

**Table 2:** Results of the MR-Egger intercept and Cochran’s Q tests for WMHs.

Exposures	MR–Egger		Cochran’s Q	
	Intercept	*p*-Value	Q	*p*-Value
Amino acids				
Phenylalanine	−0.005	0.827	0.782	0.854
Leucine	NA	NA	1.745	0.187
Valine	0.043	0.235	4.319	0.365
Tryptophan	−0.041	0.262	22.173	0.178
Polyunsaturated fatty acids				
Docosahexaenoic acid (DHA)	−0.044	0.201	5.892	0.317
Linoleic acid (LA)	−0.008	0.548	39.887	0.000
Minerals				
Copper	NA	NA	0.002	0.960
Calcium	−0.001	0.735	202.022	0.065
Iron	−0.044	0.450	3.898	0.142
Phosphorus	−0.046	0.236	4.294	0.368
Zinc	NA	NA	0.080	0.778
Magnesium	−0.013	0.365	4.390	0.495
Vitamins				
Absolute α-tocopherol	NA	NA	1.590	0.207
Relative α-tocopherol	−0.003	0.766	6.729	0.751
Relative γ-tocopherol	−0.007	0.385	13.765	0.316
25OHD	0.001	0.627	136.057	0.000
Absolute lycopene	−0.001	0.957	1.325	0.857
Relative ascorbate	−0.003	0.621	6.807	0.912
Vitamin C	0.002	0.830	3.263	0.860
Absolute retinol	NA	NA	3.111	0.078
Relative retinol	0.007	0.362	15.129	0.857

WMH, white matter hyperintensities; 25OHD, 25-hydroxyvitamin D.

## Data Availability

The exposure and outcome GWASs are available in the corresponding previous research.

## Supplementary Materials

The following supporting information can be downloaded at: https://www.mdpi.com/article/10.3390/biomedicines12040810/s1, Table S1. Characteristics of instrumental variables (IVs). Table S2. Causal effects of each nutrient on fraction anisotropy (FA) and mean diffusivity (MD) identified by different MR methods. Table S3. Results of the MR-Egger intercept and Cochran’s Q tests for fraction anisotropy (FA) and mean diffusivity (MD). Figure S1. Scatterplots of essential nutrient exposures and WMHs. Figure S2. Leave-one-out plots of essential nutrient exposures and WMHs. Figure S3. Scatterplots of essential nutrient exposures and FA. Figure S4. Leave-one-out plots of essential nutrient exposures and FA. Figure S5. Scatterplots of essential nutrient exposures and MD. Figure S6. Leave-one-out plots of essential nutrient exposures and MD.

## Abbreviations

25OHD: 25-hydroxyvitamin D; AA: arachidonic acid; ALA: alpha-linolenic acid; CI: 95% confidence interval; DTI: diffusion tensor imaging; DHA: docosahexaenoic acid; DPA: docosapentaenoic acid; DGLA: dihomogamma-linolenic acid; EPA: eicosapentaenoic acid; FA: fraction anisotropy; GLA: gamma-linolenic acid; IVW: inverse variance weighting; LA: linoleic acid; MD: mean diffusivity; MR: Mendelian randomization; MAF: minor allele frequency; OR: odds ratio; SNPs: single nucleotide polymorphisms; and WMHs: white matter hyperintensities.
