# Supplementary material for: Essential Nutrients and White Matter Hyperintensities: A Two-Sample Mendelian Randomization Study
Source: Biomedicines. 2024 Apr 6;12(4):810. doi: 10.3390/biomedicines12040810 (PMC11047968; doi:10.3390/biomedicines12040810)
Supplement: Supplementary file 1 [file biomedicines-12-00810-s001.zip › biomedicines-2903534-supplementary.pdf]

Supplementary Material

Essential Nutrients and White Matter Hyperintensities: A Two-Sample Mendelian Randomization Study

1. Supplementary Table

Table S1. Characteristics of instrumental variables (IVs)

SNP	A1	A2	A1FREQ	BETA	SE	P value	Traits	N
rs1047891	A	C	0.316449	-0.014168	0.002088	1.16E-11	25OHD	443734
rs10500209	C	T	0.282129	-0.013417	0.002169	6.18E-10	25OHD	443734
rs1065853	T	G	0.082083	0.027397	0.00367	8.32E-14	25OHD	443734
rs10818769	G	C	0.856966	-0.016972	0.00287	3.35E-09	25OHD	443734
rs10832218	C	T	0.19774	-0.034215	0.002912	7.09E-32	25OHD	443734
rs10832289	T	A	0.410173	-0.068522	0.001965	2.03E-266	25OHD	443734
rs10859995	C	T	0.581322	-0.039396	0.001971	7.03E-89	25OHD	443734
rs10887718	T	C	0.527362	-0.012476	0.001946	1.44E-10	25OHD	443734
rs11127048	A	G	0.616569	0.018107	0.002038	6.41E-19	25OHD	443734
rs111529171	C	G	0.216376	-0.015488	0.002369	6.24E-11	25OHD	443734
rs112285002	T	C	0.159529	0.060321	0.002701	1.77E-110	25OHD	443734
rs11264360	A	T	0.24292	0.018008	0.002286	3.34E-15	25OHD	443734
rs1149605	C	T	0.171453	0.01928	0.002577	7.34E-14	25OHD	443734
rs115045402	A	G	0.026334	0.10713	0.006843	3.05E-55	25OHD	443734
rs11723621	G	A	0.291123	-0.18693	0.002121	2.903E-1689	25OHD	443734
rs117576073	T	G	0.012385	-0.114573	0.008813	1.22E-38	25OHD	443734
rs117913124	A	G	0.027583	-0.354126	0.005937	1.653E-775	25OHD	443734
rs12123821	T	C	0.04793	0.074472	0.004583	2.25E-59	25OHD	443734
rs1229984	C	T	0.973099	-0.046854	0.006481	4.85E-13	25OHD	443734
rs12317268	G	A	0.151589	-0.018528	0.002717	9.15E-12	25OHD	443734
rs12803256	G	A	0.770616	0.100325	0.002325	8.599E-407	25OHD	443734
rs12997242	A	G	0.437687	-0.012512	0.001972	2.23E-10	25OHD	443734
rs144613541	G	A	0.290985	0.015413	0.002244	6.49E-12	25OHD	443734
rs145432346	C	T	0.825873	0.108617	0.003006	6.78E-286	25OHD	443734
rs157595	G	A	0.614127	-0.015581	0.00205	2.95E-14	25OHD	443734
rs17765311	C	A	0.344611	-0.01515	0.002047	1.35E-13	25OHD	443734
rs1800588	T	C	0.21461	-0.029769	0.002366	2.65E-36	25OHD	443734
rs1800775	A	C	0.486317	-0.016619	0.00195	1.56E-17	25OHD	443734
rs1858889	C	A	0.50084	0.012836	0.001942	3.85E-11	25OHD	443734
rs186881826	A	T	0.223069	0.045907	0.002469	3.64E-77	25OHD	443734
rs187443664	T	G	0.98867	-0.106498	0.013059	3.49E-16	25OHD	443734
rs188480917	G	C	0.010804	-0.343291	0.009688	5.00E-275	25OHD	443734
rs191379475	G	A	0.98893	-0.102767	0.012908	1.70E-15	25OHD	443734
rs1972994	T	A	0.64702	-0.017509	0.002036	7.99E-18	25OHD	443734
rs200641845	T	A	0.545188	0.017645	0.002356	6.92E-14	25OHD	443734
rs2011425	G	T	0.07939	-0.046356	0.00361	9.66E-38	25OHD	443734
rs201501563	T	C	0.122197	-0.066474	0.003851	9.17E-67	25OHD	443734
rs201561609	T	C	0.986941	-0.129276	0.011811	6.99E-28	25OHD	443734
rs2037511	A	G	0.165391	0.016025	0.002618	9.29E-10	25OHD	443734
rs2074735	C	G	0.064197	0.027256	0.003969	6.55E-12	25OHD	443734
rs222026	T	A	0.870776	-0.052159	0.002996	6.98E-68	25OHD	443734
rs2229742	C	G	0.103928	-0.025738	0.00319	7.13E-16	25OHD	443734
rs2585442	G	C	0.246306	0.033609	0.002287	6.87E-49	25OHD	443734
rs261291	C	T	0.35583	-0.022414	0.002033	2.89E-28	25OHD	443734
rs2762942	A	G	0.942083	0.053192	0.004321	7.99E-35	25OHD	443734
rs28364331	G	A	0.018845	0.061386	0.007186	1.31E-17	25OHD	443734
rs2847500	A	G	0.124391	-0.021129	0.002949	7.79E-13	25OHD	443734
rs2909218	T	C	0.792744	0.016894	0.002418	2.81E-12	25OHD	443734
rs2934744	A	C	0.643546	-0.022405	0.002119	3.96E-26	25OHD	443734

rs34726834	T	C	0.253615	0.013824	0.002239	6.65E-10	25OHD	443734
rs3750296	C	G	0.341423	-0.020818	0.002042	2.09E-24	25OHD	443734
rs3768013	A	G	0.369605	-0.01488	0.002011	1.37E-13	25OHD	443734
rs3775150	C	T	0.262221	-0.090838	0.002474	3.90E-295	25OHD	443734
rs3814995	T	C	0.31224	-0.014733	0.002109	2.83E-12	25OHD	443734
rs3822868	G	A	0.835029	0.021918	0.002745	1.41E-15	25OHD	443734
rs523583	C	A	0.469219	0.012174	0.001963	5.58E-10	25OHD	443734
rs532436	A	G	0.184388	-0.015051	0.002515	2.17E-09	25OHD	443734
rs560384646	C	A	0.023835	-0.193328	0.008601	6.91E-112	25OHD	443734
rs56044892	T	C	0.2107	0.015388	0.00244	2.85E-10	25OHD	443734
rs576242124	A	G	0.011317	0.114432	0.014508	3.08E-15	25OHD	443734
rs57631352	G	A	0.297255	-0.012866	0.002128	1.48E-09	25OHD	443734
rs577185477	C	T	0.01469	-0.379366	0.009583	1.624E-342	25OHD	443734
rs58073039	G	A	0.298368	-0.014119	0.002109	2.16E-11	25OHD	443734
rs58542926	T	C	0.075847	0.032488	0.00367	8.57E-19	25OHD	443734
rs6123359	G	A	0.105192	0.032345	0.003213	7.74E-24	25OHD	443734
rs6127099	T	A	0.279034	-0.036797	0.002219	9.30E-62	25OHD	443734
rs61816761	A	G	0.023096	0.125479	0.006905	8.57E-74	25OHD	443734
rs62007299	A	G	0.709377	-0.014437	0.002145	1.69E-11	25OHD	443734
rs62130059	C	A	0.335823	-0.02731	0.002255	9.25E-34	25OHD	443734
rs6438900	G	C	0.260706	0.013584	0.002221	9.59E-10	25OHD	443734
rs6698680	G	A	0.464195	-0.011928	0.001947	8.99E-10	25OHD	443734
rs6724965	G	A	0.171608	-0.016541	0.002573	1.29E-10	25OHD	443734
rs6773343	T	C	0.720232	0.01268	0.002171	5.20E-09	25OHD	443734
rs705117	T	C	0.849453	-0.034494	0.002734	1.71E-36	25OHD	443734
rs71383766	T	C	0.419574	0.012569	0.002065	1.15E-09	25OHD	443734
rs73015021	G	A	0.121031	0.023034	0.002983	1.15E-14	25OHD	443734
rs7519574	A	G	0.181694	0.016991	0.002536	2.09E-11	25OHD	443734
rs7528419	G	A	0.224958	0.019031	0.002321	2.41E-16	25OHD	443734
rs7569755	A	G	0.292374	0.013923	0.002142	8.03E-11	25OHD	443734
rs7650253	A	T	0.689684	0.014562	0.002282	1.76E-10	25OHD	443734
rs7699711	T	G	0.454848	-0.02864	0.001949	6.97E-49	25OHD	443734
rs7718395	G	C	0.319522	0.012632	0.002096	1.67E-09	25OHD	443734
rs77924615	A	G	0.197773	-0.015792	0.002464	1.46E-10	25OHD	443734
rs7828742	G	A	0.596853	-0.02193	0.00199	3.06E-28	25OHD	443734
rs78649910	A	T	0.110004	-0.018331	0.003122	4.32E-09	25OHD	443734
rs8018720	C	G	0.820235	-0.031949	0.002546	4.04E-36	25OHD	443734
rs804280	A	C	0.581959	0.013033	0.001978	4.43E-11	25OHD	443734
rs8063706	T	A	0.272828	0.012968	0.002198	3.64E-09	25OHD	443734
rs8091117	A	C	0.065396	-0.024071	0.003943	1.03E-09	25OHD	443734
rs8103262	C	T	0.305004	0.012519	0.002114	3.18E-09	25OHD	443734
rs867772	G	A	0.681808	-0.013838	0.002091	3.64E-11	25OHD	443734
rs960596	T	C	0.339588	0.012414	0.002076	2.23E-09	25OHD	443734
rs964184	C	G	0.863547	0.03977	0.002858	5.11E-44	25OHD	443734
rs9668081	T	C	0.47058	0.0116	0.001988	5.38E-09	25OHD	443734
rs33972313	C	T	0.96	5.98	1.15	2.00E-07	absolute L-ascorbic acid	15087
rs2232315	A	G	0.03	0.74	0.15	1.26E-06	absolute lycopene	441
rs341075	A	G	0.02	-0.87	0.17	5.75E-07	absolute lycopene	441
rs4635297	A	C	0.08	0.26	0.05	6.46E-07	absolute lycopene	441
rs6108801	C	T	0.04	-0.48	0.09	4.07E-07	absolute lycopene	441
rs7680948	A	C	0.2	-0.19	0.03	4.97E-09	absolute lycopene	441
rs10882272	C	T	0.35	-0.03	0.004	6.51E-15	absolute retinol	5006
rs1667255	C	A	0.31	0.03	0.004	6.35E-14	absolute retinol	5006
rs11057830	A	G	0.15	0.03	0.01	8.20E-09	absolute α-tocopherol	7781
rs2108622	T	C	0.21	0.03	0.01	1.40E-10	absolute α-tocopherol	7781
rs11645428	A	G	0.345	-0.129	0.015	1.50E-17	absolute β-carotene	3918

rs6420424	A	G	0.39	0.155	0.022	6.50E-13	absolute β-carotene	1793
rs6564851	G	T	0.395	0.149	0.015	1.60E-24	absolute β-carotene	3881
rs8044334	G	T	0.318	0.109	0.015	9.30E-13	absolute β-carotene	3915
rs16966952	G	A	0.69	0.199	0.031	2.40E-10	Arachidonic acid (AA)	8631
rs10129874	C	T	0.17	0.025	0.004	3.95E-13	Calcium	313903
rs10224210	C	T	0.72	0.019	0.003	6.87E-11	Calcium	313903
rs1035798	A	G	0.2	0.019	0.003	5.12E-09	Calcium	313903
rs1036332	C	A	0.26	0.022	0.003	2.09E-13	Calcium	313903
rs10444863	T	C	0.21	0.02	0.003	1.02E-09	Calcium	313903
rs1048603	A	G	0.31	0.02	0.003	1.05E-12	Calcium	313903
rs1064608	G	C	0.65	0.021	0.003	1.35E-14	Calcium	313903
rs10739679	G	A	0.36	0.034	0.003	9.19E-35	Calcium	313903
rs10754439	T	G	0.58	0.015	0.003	3.78E-08	Calcium	313903
rs10898822	A	G	0.51	0.015	0.003	1.91E-08	Calcium	313903
rs10917386	T	C	0.31	0.019	0.003	8.49E-12	Calcium	313903
rs11078597	C	T	0.81	0.052	0.003	9.55E-54	Calcium	313903
rs11117777	C	T	0.16	0.022	0.004	1.62E-09	Calcium	313903
rs11122848	A	G	0.47	0.015	0.003	1.67E-08	Calcium	313903
rs11187838	G	A	0.43	0.019	0.003	1.37E-12	Calcium	313903
rs11218725	G	A	0.62	0.015	0.003	2.03E-08	Calcium	313903
rs112371897	T	C	0.91	0.071	0.004	2.54E-56	Calcium	313903
rs113911787	A	G	0.77	0.017	0.003	4.13E-08	Calcium	313903
rs1150781	C	G	0.91	0.044	0.005	1.92E-21	Calcium	313903
rs11538349	C	T	0.1	0.03	0.004	1.53E-11	Calcium	313903
rs11577605	A	G	0.12	0.034	0.004	2.98E-17	Calcium	313903
rs11584885	G	A	0.28	0.026	0.003	9.85E-19	Calcium	313903
rs115946508	A	C	0.89	0.036	0.004	8.95E-18	Calcium	313903
rs116004654	T	C	0.94	0.059	0.006	9.10E-25	Calcium	313903
rs116140498	C	T	0.95	0.05	0.006	2.50E-17	Calcium	313903
rs11629876	C	T	0.33	0.018	0.003	2.93E-11	Calcium	313903
rs11632520	C	T	0.17	0.029	0.003	1.01E-16	Calcium	313903
rs11687510	C	T	0.36	0.018	0.003	1.21E-10	Calcium	313903
rs11730491	T	G	0.83	0.022	0.004	3.20E-10	Calcium	313903
rs11746728	C	T	0.65	0.016	0.003	4.74E-09	Calcium	313903
rs11753096	C	T	0.46	0.019	0.003	6.89E-13	Calcium	313903
rs1177274	T	G	0.56	0.028	0.003	1.45E-26	Calcium	313903
rs11777067	T	C	0.77	0.02	0.003	3.90E-11	Calcium	313903
rs11792928	C	T	0.29	0.017	0.003	5.27E-09	Calcium	313903
rs12212449	C	T	0.61	0.031	0.003	9.55E-31	Calcium	313903
rs12294466	T	C	0.92	0.029	0.005	4.97E-09	Calcium	313903
rs12339541	C	A	0.06	0.056	0.005	1.22E-25	Calcium	313903
rs12378991	G	A	0.08	0.036	0.005	1.08E-13	Calcium	313903
rs12519940	C	T	0.28	0.027	0.003	2.75E-20	Calcium	313903
rs12534476	G	A	0.17	0.021	0.003	1.07E-09	Calcium	313903
rs12583851	T	C	0.75	0.022	0.003	1.48E-13	Calcium	313903
rs1260326	T	C	0.61	0.047	0.003	3.46E-69	Calcium	313903
rs12613807	C	T	0.56	0.018	0.003	9.55E-12	Calcium	313903
rs1262217	A	G	0.17	0.02	0.003	4.99E-09	Calcium	313903
rs12675477	T	C	0.73	0.017	0.003	1.61E-08	Calcium	313903
rs12794834	C	T	0.52	0.015	0.003	3.08E-08	Calcium	313903
rs12917235	C	T	0.37	0.027	0.003	2.33E-23	Calcium	313903
rs12918968	A	C	0.44	0.032	0.003	1.06E-34	Calcium	313903
rs12922549	C	T	0.24	0.023	0.003	2.42E-13	Calcium	313903
rs12932755	A	G	0.51	0.017	0.003	1.62E-10	Calcium	313903
rs12933677	T	C	0.47	0.015	0.003	6.97E-09	Calcium	313903
rs12974855	A	G	0.86	0.026	0.004	1.97E-11	Calcium	313903

rs12998379	G	A	0.19	0.026	0.003	1.43E-14	Calcium	313903
rs1303	T	G	0.74	0.021	0.003	4.69E-12	Calcium	313903
rs13107325	C	T	0.93	0.059	0.005	1.14E-32	Calcium	313903
rs13108218	A	G	0.62	0.039	0.003	4.07E-47	Calcium	313903
rs13254847	C	T	0.22	0.023	0.003	1.33E-12	Calcium	313903
rs13325	G	A	0.17	0.025	0.004	1.44E-12	Calcium	313903
rs13389219	C	T	0.39	0.016	0.003	1.02E-09	Calcium	313903
rs1354034	T	C	0.6	0.019	0.003	3.11E-13	Calcium	313903
rs1476698	A	G	0.37	0.023	0.003	7.70E-17	Calcium	313903
rs1495747	C	T	0.28	0.019	0.003	2.36E-10	Calcium	313903
rs1604081	C	T	0.14	0.022	0.004	7.93E-09	Calcium	313903
rs1621676	G	A	0.25	0.017	0.003	3.96E-08	Calcium	313903
rs1657502	T	G	0.62	0.02	0.003	4.21E-13	Calcium	313903
rs16844401	A	G	0.07	0.051	0.005	1.31E-21	Calcium	313903
rs1688131	T	C	0.71	0.036	0.003	1.79E-35	Calcium	313903
rs17164683	C	T	0.27	0.02	0.003	1.66E-11	Calcium	313903
rs1749849	C	T	0.42	0.021	0.003	2.47E-15	Calcium	313903
rs17668044	A	G	0.71	0.018	0.003	4.66E-10	Calcium	313903
rs17774672	G	A	0.16	0.025	0.004	2.09E-12	Calcium	313903
rs1780316	C	T	0.06	0.037	0.006	1.37E-11	Calcium	313903
rs1801282	C	G	0.88	0.039	0.004	7.95E-22	Calcium	313903
rs1827293	G	A	0.45	0.024	0.003	2.97E-19	Calcium	313903
rs1858800	T	C	0.65	0.032	0.003	1.61E-30	Calcium	313903
rs1875272	G	A	0.75	0.026	0.003	9.81E-17	Calcium	313903
rs2004315	T	C	0.38	0.033	0.003	5.84E-35	Calcium	313903
rs2070179	C	T	0.24	0.017	0.003	3.40E-08	Calcium	313903
rs2100431	A	C	0.3	0.017	0.003	3.04E-09	Calcium	313903
rs218671	G	T	0.55	0.016	0.003	6.44E-10	Calcium	313903
rs2245715	A	G	0.1	0.024	0.004	4.45E-08	Calcium	313903
rs2288920	G	T	0.83	0.026	0.004	1.12E-13	Calcium	313903
rs2298615	C	T	0.23	0.019	0.003	1.27E-09	Calcium	313903
rs2303914	G	T	0.52	0.02	0.003	2.07E-13	Calcium	313903
rs2343592	A	G	0.27	0.023	0.003	2.71E-15	Calcium	313903
rs2419886	C	T	0.26	0.023	0.003	2.91E-14	Calcium	313903
rs2448036	G	T	0.11	0.029	0.004	4.28E-11	Calcium	313903
rs2520265	A	G	0.29	0.022	0.003	5.23E-14	Calcium	313903
rs2546056	T	C	0.86	0.026	0.004	8.33E-12	Calcium	313903
rs2585135	A	G	0.54	0.015	0.003	1.85E-08	Calcium	313903
rs2636695	G	T	0.77	0.021	0.003	2.74E-11	Calcium	313903
rs2681417	G	A	0.93	0.037	0.005	2.82E-13	Calcium	313903
rs2785171	G	A	0.61	0.015	0.003	1.76E-08	Calcium	313903
rs2807880	C	T	0.18	0.02	0.003	1.87E-09	Calcium	313903
rs2855799	G	A	0.81	0.018	0.003	4.06E-08	Calcium	313903
rs28616221	A	G	0.82	0.046	0.003	4.53E-42	Calcium	313903
rs2918247	A	G	0.23	0.017	0.003	2.48E-08	Calcium	313903
rs3026445	T	C	0.37	0.018	0.003	2.52E-11	Calcium	313903
rs302655	G	T	0.43	0.02	0.003	3.55E-14	Calcium	313903
rs308981	C	T	0.12	0.023	0.004	2.25E-08	Calcium	313903
rs3130618	C	A	0.8	0.021	0.003	2.50E-10	Calcium	313903
rs3217795	A	G	0.09	0.025	0.005	4.95E-08	Calcium	313903
rs34010237	A	G	0.84	0.03	0.004	1.39E-17	Calcium	313903
rs34372369	A	G	0.05	0.044	0.006	1.27E-13	Calcium	313903
rs34667500	A	G	0.88	0.038	0.004	3.14E-21	Calcium	313903
rs34895054	C	G	0.26	0.019	0.003	4.86E-10	Calcium	313903
rs35320690	C	T	0.72	0.026	0.003	4.71E-19	Calcium	313903
rs35587941	T	G	0.57	0.015	0.003	1.37E-08	Calcium	313903

rs35674179	C	A	0.9	0.026	0.004	2.00E-09	Calcium	313903
rs35758545	C	T	0.35	0.016	0.003	3.10E-09	Calcium	313903
rs35852840	A	C	0.94	0.031	0.006	4.70E-08	Calcium	313903
rs36086195	T	C	0.42	0.018	0.003	3.78E-12	Calcium	313903
rs36104352	C	A	0.88	0.03	0.004	7.60E-14	Calcium	313903
rs3740690	T	C	0.44	0.015	0.003	1.92E-08	Calcium	313903
rs3741628	G	T	0.57	0.022	0.003	1.24E-16	Calcium	313903
rs3794695	T	C	0.81	0.019	0.003	1.21E-08	Calcium	313903
rs3798236	T	C	0.37	0.017	0.003	7.93E-10	Calcium	313903
rs3931841	A	G	0.68	0.027	0.003	2.37E-21	Calcium	313903
rs41264630	A	G	0.92	0.027	0.005	1.51E-08	Calcium	313903
rs413948	G	A	0.87	0.022	0.004	2.59E-08	Calcium	313903
rs4376797	A	G	0.65	0.026	0.003	5.75E-21	Calcium	313903
rs4594967	A	G	0.67	0.023	0.003	7.83E-17	Calcium	313903
rs4633480	A	G	0.44	0.021	0.003	2.43E-15	Calcium	313903
rs4758621	A	G	0.31	0.028	0.003	2.55E-22	Calcium	313903
rs4763297	C	A	0.57	0.015	0.003	5.80E-09	Calcium	313903
rs4790310	C	T	0.57	0.022	0.003	3.31E-17	Calcium	313903
rs4805129	T	C	0.63	0.02	0.003	1.73E-13	Calcium	313903
rs4915444	C	A	0.4	0.017	0.003	2.52E-10	Calcium	313903
rs4917	C	T	0.36	0.038	0.003	5.43E-43	Calcium	313903
rs4925104	G	T	0.47	0.018	0.003	1.90E-11	Calcium	313903
rs4976647	C	A	0.67	0.018	0.003	1.03E-10	Calcium	313903
rs498490	C	T	0.16	0.033	0.004	8.71E-21	Calcium	313903
rs507666	G	A	0.19	0.021	0.003	2.69E-10	Calcium	313903
rs55633823	C	T	0.75	0.022	0.003	1.45E-12	Calcium	313903
rs55754498	C	T	0.05	0.033	0.006	2.37E-08	Calcium	313903
rs56230940	T	C	0.93	0.031	0.005	3.20E-10	Calcium	313903
rs56252617	T	C	0.6	0.015	0.003	4.02E-08	Calcium	313903
rs56313825	A	G	0.6	0.015	0.003	1.44E-08	Calcium	313903
rs56397046	C	T	0.33	0.021	0.003	2.24E-14	Calcium	313903
rs5751350	A	G	0.67	0.016	0.003	1.75E-08	Calcium	313903
rs57564578	G	A	0.07	0.033	0.005	1.08E-10	Calcium	313903
rs58673639	G	T	0.79	0.018	0.003	6.34E-09	Calcium	313903
rs6013892	A	C	0.93	0.032	0.005	6.19E-10	Calcium	313903
rs60155540	G	A	0.2	0.021	0.003	2.57E-10	Calcium	313903
rs611150	T	C	0.82	0.022	0.003	9.18E-11	Calcium	313903
rs6118	C	T	0.9	0.03	0.004	8.86E-12	Calcium	313903
rs61594679	C	T	0.29	0.018	0.003	4.23E-10	Calcium	313903
rs61649210	G	A	0.45	0.015	0.003	1.35E-08	Calcium	313903
rs61770531	C	T	0.91	0.036	0.005	8.13E-15	Calcium	313903
rs61779309	T	C	0.21	0.02	0.003	9.79E-10	Calcium	313903
rs62134669	C	T	0.85	0.024	0.004	1.22E-10	Calcium	313903
rs62292542	G	A	0.11	0.032	0.004	2.37E-14	Calcium	313903
rs62362239	C	T	0.67	0.02	0.003	7.53E-13	Calcium	313903
rs62439474	T	C	0.7	0.022	0.003	4.81E-14	Calcium	313903
rs6560613	C	T	0.91	0.034	0.005	6.79E-13	Calcium	313903
rs6580981	G	A	0.46	0.019	0.003	4.69E-13	Calcium	313903
rs6590227	C	T	0.12	0.025	0.004	5.42E-10	Calcium	313903
rs66527777	C	T	0.17	0.022	0.004	2.79E-10	Calcium	313903
rs6680117	C	T	0.18	0.027	0.003	3.20E-15	Calcium	313903
rs6719061	C	T	0.6	0.019	0.003	6.19E-13	Calcium	313903
rs6722613	G	A	0.57	0.016	0.003	5.31E-10	Calcium	313903
rs6731551	T	C	0.48	0.015	0.003	1.26E-08	Calcium	313903
rs6734610	G	A	0.48	0.023	0.003	1.58E-18	Calcium	313903
rs6741561	C	T	0.39	0.04	0.003	1.85E-50	Calcium	313903

rs681664	C	T	0.22	0.018	0.003	1.27E-08	Calcium	313903
rs6830950	T	C	0.27	0.017	0.003	1.50E-08	Calcium	313903
rs6841429	C	A	0.17	0.041	0.004	1.02E-31	Calcium	313903
rs6894167	C	T	0.52	0.017	0.003	5.07E-11	Calcium	313903
rs6909201	A	G	0.48	0.043	0.003	4.12E-60	Calcium	313903
rs697852	G	A	0.82	0.022	0.003	3.13E-10	Calcium	313903
rs7012637	A	G	0.52	0.023	0.003	5.48E-18	Calcium	313903
rs7208714	G	A	0.75	0.027	0.003	1.75E-19	Calcium	313903
rs7221118	T	C	0.21	0.021	0.003	1.18E-10	Calcium	313903
rs722298	A	G	0.56	0.016	0.003	3.38E-09	Calcium	313903
rs72740967	C	T	0.31	0.02	0.003	1.15E-12	Calcium	313903
rs72847071	G	A	0.09	0.03	0.005	7.17E-11	Calcium	313903
rs72999033	T	C	0.93	0.043	0.005	5.12E-16	Calcium	313903
rs7312673	A	G	0.07	0.043	0.005	2.41E-17	Calcium	313903
rs7313874	T	C	0.6	0.017	0.003	6.13E-10	Calcium	313903
rs73183155	C	T	0.23	0.028	0.003	1.40E-18	Calcium	313903
rs7320843	C	T	0.14	0.036	0.004	2.60E-22	Calcium	313903
rs74753001	C	T	0.93	0.028	0.005	3.12E-08	Calcium	313903
rs7559013	C	A	0.87	0.026	0.004	2.07E-11	Calcium	313903
rs7568296	T	C	0.42	0.02	0.003	5.83E-14	Calcium	313903
rs760077	A	T	0.61	0.027	0.003	1.84E-23	Calcium	313903
rs76758508	C	T	0.32	0.015	0.003	4.19E-08	Calcium	313903
rs77722590	A	G	0.85	0.023	0.004	2.38E-10	Calcium	313903
rs7864156	G	T	0.61	0.018	0.003	1.42E-11	Calcium	313903
rs7913072	G	A	0.86	0.021	0.004	3.95E-08	Calcium	313903
rs7968405	C	T	0.82	0.022	0.003	2.24E-10	Calcium	313903
rs8011945	G	T	0.09	0.026	0.005	1.14E-08	Calcium	313903
rs80339483	T	C	0.93	0.031	0.005	1.81E-09	Calcium	313903
rs8034835	A	G	0.47	0.019	0.003	9.95E-13	Calcium	313903
rs80350997	A	G	0.92	0.046	0.005	1.03E-20	Calcium	313903
rs8081353	C	T	0.1	0.025	0.004	1.21E-08	Calcium	313903
rs838718	G	A	0.48	0.047	0.003	9.09E-73	Calcium	313903
rs841572	A	G	0.59	0.033	0.003	1.30E-35	Calcium	313903
rs848492	A	G	0.28	0.027	0.003	1.01E-19	Calcium	313903
rs872629	A	C	0.89	0.02404	0.004	6.00E-09	Calcium	313903
rs900399	G	A	0.6	0.019	0.003	3.79E-12	Calcium	313903
rs915894	T	G	0.6	0.015	0.003	1.70E-08	Calcium	313903
rs926103	C	T	0.35	0.022	0.003	8.02E-16	Calcium	313903
rs9282641	G	A	0.92	0.036	0.005	2.71E-14	Calcium	313903
rs9388399	T	C	0.31	0.024	0.003	1.94E-17	Calcium	313903
rs9420589	T	G	0.56	0.017	0.003	6.74E-11	Calcium	313903
rs9530	A	G	0.55	0.032	0.003	3.64E-34	Calcium	313903
rs9562385	A	C	0.56	0.015	0.003	1.08E-08	Calcium	313903
rs9635741	C	A	0.93	0.039	0.005	8.14E-15	Calcium	313903
rs965344	A	G	0.79	0.031	0.003	1.08E-21	Calcium	313903
rs9806062	G	T	0.89	0.026	0.004	5.10E-10	Calcium	313903
rs9895661	C	T	0.83	0.028	0.003	5.04E-16	Calcium	313903
rs1175550	G	A	0.22	0.198	0.032	5.03E-10	Copper	2603
rs2769264	G	T	0.16	0.313	0.034	2.63E-20	Copper	2603
rs11604424	T	C	0.756672	-0.08311	0.014241	7.84E-09	Docosahexaenoic acid (DHA)	13495
rs143988316	T	C	0.069487	-0.150045	0.024351	1.10E-09	Docosahexaenoic acid (DHA)	13494
rs145717049	T	C	0.044058	-0.201292	0.03275	1.21E-09	Docosahexaenoic acid (DHA)	13491
rs174546	T	C	0.402849	-0.127635	0.012483	4.81E-24	Docosahexaenoic acid (DHA)	13499
rs2281591	G	A	0.13372	-0.108394	0.018174	3.66E-09	Docosahexaenoic acid (DHA)	13498
rs261334	C	G	0.769129	-0.110247	0.014749	1.44E-13	Docosahexaenoic acid (DHA)	13498
rs3734398	C	T	0.43	0.04	0.003	1.00E-43	Docosapentaenoic acid (DPA)	8631

rs174538	G	A	0.72	0.083	0.005	5.00E-58	Eicosapentaenoic acid (EPA)	8631
rs3798713	C	G	0.43	0.035	0.005	2.00E-12	Eicosapentaenoic acid (EPA)	8631
rs1799945	G	C	0.15	0.189	0.01	1.10E-81	Iron	48972
rs1800562	A	G	0.07	0.328	0.016	2.72E-97	Iron	48972
rs855791	G	A	0.55	0.181	0.007	1.32E-139	Iron	48972
rs1440580	A	T	0.481233	0.072612	0.00941	1.68E-14	Isoleucine	24772
rs12325419	A	G	0.120221	-0.082062	0.014985	4.55E-08	Leucine	22500
rs17789027	G	A	0.384329	0.108594	0.009541	6.16E-30	Leucine	24725
rs10402112	A	T	0.09963	-0.171088	0.021087	7.90E-16	Linoleic acid (LA)	13522
rs12239737	A	T	0.260158	-0.100493	0.014041	1.20E-12	Linoleic acid (LA)	13523
rs144064722	G	A	0.026298	0.230203	0.039539	7.45E-09	Linoleic acid (LA)	13520
rs144723570	T	C	0.01117	-0.332453	0.059815	3.42E-08	Linoleic acid (LA)	13523
rs174418	C	T	0.562555	-0.086064	0.012538	9.42E-12	Linoleic acid (LA)	13524
rs190934192	A	G	0.027535	-0.248268	0.043367	1.32E-08	Linoleic acid (LA)	13523
rs4296389	T	C	0.325777	-0.081438	0.013081	6.37E-10	Linoleic acid (LA)	13521
rs4609471	A	C	0.041643	-0.253291	0.035446	1.30E-12	Linoleic acid (LA)	13526
rs7412	T	C	0.057292	-0.295042	0.028251	3.40E-25	Linoleic acid (LA)	13523
rs76366838	A	G	0.018475	0.287426	0.049651	9.06E-09	Linoleic acid (LA)	13524
rs769449	A	G	0.156683	0.142266	0.016956	8.04E-17	Linoleic acid (LA)	13524
rs79225634	T	C	0.35159	0.096057	0.012933	1.66E-13	Linoleic acid (LA)	13523
rs821840	G	A	0.25211	0.083597	0.014639	1.43E-08	Linoleic acid (LA)	13523
rs9804646	T	C	0.124469	-0.105674	0.019115	4.05E-08	Linoleic acid (LA)	13520
rs99780	T	C	0.401006	0.148368	0.012472	3.35E-32	Linoleic acid (LA)	13523
rs2863979	a	g	0.7241	0.0143	0.0017	1.44E-17	lysine	7812
rs11144134	C	T	0.08	0.011	0.001	8.21E-15	Magnesium	23829
rs13146355	A	G	0.44	0.005	0.001	6.27E-13	Magnesium	23829
rs3925584	T	C	0.55	0.006	0.001	5.20E-16	Magnesium	23829
rs4072037	T	C	0.54	0.01	0.001	2.01E-36	Magnesium	23829
rs448378	A	G	0.53	0.004	0.001	1.25E-08	Magnesium	23829
rs7965584	A	G	0.71	0.007	0.001	1.05E-16	Magnesium	23829
rs320485	t	c	0.1324	0.0094	0.0017	4.97E-08	methionine	7795
rs1718309	G	A	0.602584	-0.077165	0.009719	2.50E-15	Phenylalanine	22660
rs182695896	C	A	0.018066	0.245321	0.040112	1.09E-09	Phenylalanine	21467
rs2731672	C	T	0.740037	0.094918	0.011618	3.85E-16	Phenylalanine	20436
rs4253238	T	C	0.555072	0.067292	0.010053	2.53E-11	Phenylalanine	20435
rs1697421	A	G	0.49	0.05	0.005	1.14E-27	Phosphorus	21726
rs17265703	A	G	0.85	0.036	0.006	4.32E-09	Phosphorus	21733
rs2970818	A	T	0.09	0.047	0.008	4.38E-09	Phosphorus	21734
rs9469578	C	T	0.92	0.059	0.009	1.11E-11	Phosphorus	21734
rs947583	C	T	0.29	0.035	0.005	3.45E-12	Phosphorus	21733
rs11167905	C	T	0.15	-0.08	0.0164	9.83E-07	relative ascorbate	2063
rs13069990	T	C	0.62	-0.05	0.011	4.44E-06	relative ascorbate	2063
rs13103690	G	T	0.46	0.05	0.0104	5.20E-06	relative ascorbate	2063
rs2070006	C	T	0.63	-0.05	0.0112	4.76E-06	relative ascorbate	2063
rs577596	A	G	0.33	-0.06	0.0114	6.68E-07	relative ascorbate	2063
rs6713914	C	T	0.43	-0.06	0.0116	3.22E-07	relative ascorbate	2063
rs6826474	T	C	0.04	-0.14	0.0288	1.56E-06	relative ascorbate	2063
rs6834631	G	T	0.04	-0.13	0.0267	1.03E-06	relative ascorbate	2063
rs7112460	T	C	0.07	0.11	0.0223	1.14E-06	relative ascorbate	2063
rs8057559	T	C	0.03	0.14	0.0314	9.10E-06	relative ascorbate	2063
rs808686	A	G	0.49	0.06	0.0128	3.01E-06	relative ascorbate	2063
rs8105491	T	G	0.85	-0.07	0.0148	2.30E-06	relative ascorbate	2063
rs9419004	C	G	0.19	-0.25	0.0563	6.53E-06	relative ascorbate	2063
rs9606290	A	G	0.24	0.16	0.0352	6.32E-06	relative ascorbate	2063
rs10019071	A	G	0.019	0.66	0.16	3.64E-06	relative retinol	1957
rs112293959	G	A	0.03117	-0.43	0.13	5.70E-06	relative retinol	1957

rs114515641	G	T	0.03	0.41	0.13	7.12E-06	relative retinol	1957
rs1153379	A	G	0.92872	-0.32	0.08	6.10E-06	relative retinol	1957
rs1176744	C	A	0.3183	-0.21	0.05	3.51E-07	relative retinol	1957
rs118025446	A	G	0.03091	-0.48	0.12	9.84E-06	relative retinol	1957
rs12955464	G	C	0.1423	-0.23	0.06	3.71E-06	relative retinol	1957
rs139726207	G	A	0.03883	0.37	0.11	4.46E-06	relative retinol	1957
rs149478645	G	A	0.02402	-0.51	0.14	1.30E-06	relative retinol	1957
rs17005512	C	G	0.17	-0.22	0.06	2.77E-06	relative retinol	1957
rs1842947	G	A	0.5217	-0.19	0.04	8.34E-07	relative retinol	1957
rs2147337	G	T	0.66	0.16	0.04	9.00E-06	relative retinol	1957
rs2367816	G	A	0.7713	0.23	0.05	9.46E-06	relative retinol	1957
rs2417325	T	C	0.93306	0.33	0.08	1.29E-06	relative retinol	1957
rs3890033	C	T	0.3835	0.14	0.04	8.56E-06	relative retinol	1957
rs3898702	T	C	0.1995	-0.22	0.05	3.02E-06	relative retinol	1957
rs4135385	G	A	0.2366	0.21	0.05	9.80E-06	relative retinol	1957
rs58411567	A	G	0.2156	-0.21	0.05	3.02E-07	relative retinol	1957
rs6550239	A	G	0.7422	-0.18	0.05	4.40E-06	relative retinol	1957
rs75308833	T	C	0.02248	-0.49	0.15	3.51E-06	relative retinol	1957
rs7926028	T	G	0.4451	-0.13	0.04	2.75E-06	relative retinol	1957
rs945817	A	G	0.1924	-0.28	0.05	6.46E-07	relative retinol	1957
rs9586119	C	T	0.07282	0.35	0.08	3.34E-06	relative retinol	1957
rs10163969	T	G	0.04	-0.04	0.008	9.39E-06	relative α-tocopherol	7276
rs10245705	T	C	0.02	-0.07	0.0127	1.95E-07	relative α-tocopherol	7276
rs10935814	A	G	0.1	-0.04	0.0083	9.44E-06	relative α-tocopherol	7276
rs11145330	A	C	0.89	0.03	0.0068	1.95E-06	relative α-tocopherol	7276
rs11992435	A	G	0.95	0.03	0.0072	6.38E-06	relative α-tocopherol	7276
rs1404410	C	G	0.79	-0.02	0.0052	4.57E-06	relative α-tocopherol	7276
rs1532701	A	G	0.55	0.01	0.003	5.07E-06	relative α-tocopherol	7276
rs2074731	A	C	0.17	-0.02	0.0039	2.31E-06	relative α-tocopherol	7276
rs261342	C	G	0.79	-0.02	0.0037	5.41E-06	relative α-tocopherol	7276
rs7238006	T	C	0.93	0.03	0.0057	6.77E-07	relative α-tocopherol	7276
rs7930821	T	C	0.02	0.07	0.0149	7.53E-06	relative α-tocopherol	7276
rs10077932	T	C	0.14	-0.04	0.0087	4.08E-06	relative γ-tocopherol	5822
rs1013104	T	C	0.44	-0.02	0.0045	3.83E-06	relative γ-tocopherol	5822
rs10466757	A	T	0.16	0.06	0.0143	9.56E-06	relative γ-tocopherol	5822
rs10492212	T	C	0.16	-0.03	0.0062	8.66E-06	relative γ-tocopherol	5822
rs10520845	A	C	0.02	0.19	0.0419	5.27E-06	relative γ-tocopherol	5822
rs1060467	A	G	0.59	0.02	0.0045	2.61E-07	relative γ-tocopherol	5822
rs13336771	A	C	0.17	0.06	0.0139	7.39E-06	relative γ-tocopherol	5822
rs261301	T	C	0.13	0.03	0.0068	2.06E-06	relative γ-tocopherol	5822
rs2794327	T	C	0.67	-0.04	0.008	8.78E-06	relative γ-tocopherol	5822
rs5994305	A	G	0.83	0.03	0.0062	7.15E-07	relative γ-tocopherol	5822
rs6821770	A	G	0.14	0.04	0.0086	8.92E-06	relative γ-tocopherol	5822
rs7038957	T	C	0.83	-0.03	0.0062	3.86E-06	relative γ-tocopherol	5822
rs7350776	C	G	0.7	0.02	0.0052	3.86E-06	relative γ-tocopherol	5822
rs1016522	A	G	0.5803	0.0058	0.0009	1.59E-10	tryotophan	7804
rs13122250	T	C	0.5542	0.0062	0.0009	8.95E-12	tryotophan	7804
rs1373962	T	C	0.5973	0.005	0.0009	2.71E-08	tryotophan	7804
rs1559063	C	G	0.6209	0.0052	0.0009	7.82E-09	tryotophan	7804
rs2111118	T	C	0.6207	0.0051	0.0009	1.21E-08	tryotophan	7804
rs284191	A	G	0.615	-0.006	0.001	1.97E-09	tryotophan	7804
rs38271	A	G	0.5925	-0.0051	0.0009	1.19E-08	tryotophan	7804
rs4306882	T	G	0.6153	-0.0057	0.0009	2.52E-10	tryotophan	7804
rs4615256	A	G	0.5327	0.0049	0.0009	4.99E-08	tryotophan	7804
rs4695138	A	T	0.5352	0.0052	0.0009	8.00E-09	tryotophan	7804
rs4958379	A	G	0.433	-0.005	0.0009	2.18E-08	tryotophan	7804

rs603446	T	C	0.4448	0.0051	0.0009	1.38E-08	tryotophan	7804
rs6480970	A	G	0.6159	-0.0049	0.0009	4.29E-08	tryotophan	7804
rs6901004	C	G	0.5742	-0.0061	0.0009	1.08E-11	tryotophan	7804
rs6935961	A	G	0.5578	-0.0056	0.0009	3.75E-10	tryotophan	7804
rs710580	A	C	0.3566	-0.005	0.0009	3.57E-08	tryotophan	7804
rs7463805	T	C	0.5213	-0.0053	0.0009	4.60E-09	tryotophan	7804
rs7584842	T	C	0.5377	-0.005	0.0009	4.15E-08	tryotophan	7804
rs9511152	A	G	0.559	-0.005	0.0009	2.99E-08	tryotophan	7804
rs972459	T	C	0.5787	0.005	0.0009	1.97E-08	tryotophan	7804
rs10211524	A	G	0.40991	0.086403	0.009427	5.24E-20	Valine	24898
rs2072560	C	T	0.928539	0.104738	0.017688	3.28E-09	Valine	24895
rs7406661	C	T	0.243378	0.079073	0.012727	5.35E-10	Valine	22659
rs7655059	G	C	0.217667	-0.068459	0.011163	8.91E-10	Valine	24897
rs9637599	C	A	0.46958	0.113927	0.009156	1.67E-35	Valine	24899
rs1256335	A	G	0.79	0.14	0.02	1.40E-15	Vitamin B6	4763
rs4654748	T	C	0.52	0.1	0.01	4.30E-11	Vitamin B6	4763
rs10051765	C	T	0.342	0.039	0.007	3.64E-09	vitamin C	52018
rs10136000	A	G	0.283	0.04	0.007	1.33E-08	vitamin C	52018
rs117885456	A	G	0.087	0.078	0.012	1.70E-11	vitamin C	52018
rs13028225	T	C	0.857	0.102	0.009	2.38E-30	vitamin C	52018
rs174547	C	T	0.328	0.036	0.007	3.84E-08	vitamin C	52018
rs2559850	A	G	0.598	0.058	0.006	6.30E-20	vitamin C	52018
rs56738967	C	G	0.321	0.041	0.007	7.62E-10	vitamin C	52018
rs6693447	T	G	0.551	0.039	0.006	6.25E-10	vitamin C	52018
rs7740812	G	A	0.594	0.038	0.006	1.88E-09	vitamin C	52018
rs1532423	A	G	0.37	0.178	0.026	6.40E-12	Zinc	2603
rs2120019	T	C	0.79	0.287	0.033	1.55E-18	Zinc	2603

Table S2. Causal effects of each nutrient on fraction anisotropy (FA) and mean diffusivity (MD) identified by different MR methods.

Exposures	Methods	No. SNPs	FA		MD	
			OR（95%CI）	*p*-Value	OR (95%CI)	p-Value
**Amino acids**						
Phenylalanine	Simple median	4	1.197(0.573,2.497)	0.632	0.705(0.344,1.444)	0.339
	Weighted median	4	1.043(0.517,2.106)	0.907	0.782(0.390,1.567)	0.488
	MR Egger	4	1.169(0.133,10.304)	0.901	0.729(0.079,6.727)	0.806
	IVW	4	1.180(0.655,2.125)	0.582	0.799(0.438,1.457)	0.464
Leucine	Simple median	3	1.561(0.783,3.113)	0.206	1.153(0.518,2.567)	0.727
	Weighted median	3	1.645(0.893,3.030)	0.110	1.030(0.540,1.965)	0.929
	MR Egger	3	2.393(0.049,117.552)	0.737	0.278(0.005,14.878)	0.642
	IVW	3	1.651(0.925,2.949)	0.090	1.121(0.620,2.028)	0.704
Valine	Simple median	4	1.002(0.482,2.083)	0.995	1.308(0.638,2.685)	0.464
	Weighted median	4	1.274(0.653,2.485)	0.478	0.825(0.444,1.536)	0.545
	MR Egger	4	21.965(0.762,633.411)	0.213	0.130(0.004,4.041)	0.365
	IVW	4	1.307(0.780,2.192)	0.310	0.997(0.585,1.701)	0.992
Tryotophan	Simple median	17	56.239(0.224,14122.721)	0.153	8.882(0.025,3148.921)	0.466
	Weighted median	17	31.565(0.120,8284.921)	0.224	6.263(0.016,2507.617)	0.548
	MR Egger	17	46.048(0.000, 2.873E+26)	0.897	0.000(0.000,5.671E+22)	0.769
	IVW	17	9.750(0.146,651.319)	0.288	0.666(0.007,63.795)	0.862
**Polyunsaturated fatty acids**						
Dihomo-gamma-linolenic acid (DGLA)	IVW	2	0.727(0.588,0.899)	0.003	1.200(0.969,1.488)	0.095
Docosahexaenoic acid (DHA)	Simple median	5	1.464(0.695,3.081)	0.316	1.056(0.557,2.000)	0.868
	Weighted median	5	1.575(0.818,3.033)	0.174	0.839(0.487,1.446)	0.527
	MR Egger	5	14.639(1.26,170.016)	0.121	1.651(0.185,14.763)	0.684
	IVW	5	1.533(0.816,2.881)	0.184	0.830(0.531,1.297)	0.413
Docosapentaenoic acid (DPA)	IVW	2	3.798(0.687,20.981)	0.126	0.948(0.196,4.577)	0.947
	Simple median	3	\	\	0.568(0.198,1.634)	0.295
	Weighted median	3	\	\	0.310(0.037,2.608)	0.476
	MR Egger	3	\	\	0.577(0.209,1.592)	0.288
Gamma linolenic acid (GLA)	IVW	2	1131.012(0.156,8203097.297)	0.121	0.122(0.000,392.412)	0.610
Arachidonic acid (AA)	IVW	2	1.081(1.027,1.138)	0.003	0.971(0.908,1.038)	0.381
Linoleic acid (LA)	Simple median	15	0.788(0.567,1.094)	0.154	1.243(0.889,1.738)	0.203
	Weighted median	15	0.776(0.579,1.040)	0.089	1.248(0.924,1.684)	0.148
	MR Egger	15	0.874(0.516,1.479)	0.623	0.896(0.476,1.685)	0.738
	IVW	15	0.776(0.616,0.978)	0.032	1.174(0.884,1.559)	0.269
**Minerals**						
Copper	IVW	2	1.045(0.527,2.072)	0.899	1.079(0.625,1.861)	0.785
Calcium	Simple median	170	0.852(0.533,1.363)	0.504	1.237(0.788,1.941)	0.356
	Weighted median	170	0.734(0.456,1.180)	0.202	1.419(0.875,2.301)	0.156
	MR Egger	170	0.649(0.238,1.769)	0.399	2.915(1.054,8.067)	0.041
	IVW	170	0.880(0.626,1.238)	0.464	1.147(0.809,1.627)	0.440
Iron	IVW	2	1.106(0.795,1.539)	0.549	0.963(0.687,1.350)	0.828
Phosphorus	Simple median	4	2.326(0.547,9.879)	0.253	0.849(0.181,3.987)	0.835
	Weighted median	4	1.547(0.400,5.991)	0.527	1.511(0.378,6.034)	0.559
	MR Egger	4	3.821(0.009,1579.634)	0.705	2.095(0.000,15046.597)	0.885

	IVW	4	1.723(0.534,5.556)	0.363	0.976(0.236,4.040)	0.974
Zinc	IVW	2	1.370(0.937,2.003)	0.105	0.783(0.583,1.051)	0.104
Magnesium	Simple median	6	0.355(0,807.923)	0.793	3.086(0.001,11821.779)	0.789
	Weighted median	6	0.090(0.000,87.584)	0.492	16.662(0.013,21367.822)	0.441
	MR Egger	6	0.000(0.000,3054.916)	0.389	67101.982(0.006,6.982E+11)	0.249
	IVW	6	0.028(0.000,6.920)	0.204	86.438(0.313,23892.511)	0.120
**Vitamins**						
Absolute α-tocopherol	IVW	2	1.699(0.156,18.524)	0.663	0.730(0.001,364.012)	0.921
Relative α-tocopherol	Simple median	9	1.219(0.148,10.054)	0.854	0.617(0.065,5.858)	0.674
	Weighted median	9	1.126(0.134,9.439)	0.913	0.518(0.064,4.198)	0.537
	MR Egger	9	1.516(0.067,34.224)	0.801	0.503(0.021,12.155)	0.685
	IVW	9	1.301(0.273,6.204)	0.741	0.638(0.129,3.149)	0.581
Relative γ-tocopherol	Simple median	11	1.261(0.408,3.896)	0.687	1.337(0.339,5.269)	0.678
	Weighted median	11	1.367(0.470,3.975)	0.566	1.344(0.381,4.735)	0.646
	MR Egger	11	1.344(0.210,8.582)	0.762	0.915(0.093,9.049)	0.941
	IVW	11	1.170(0.502,2.726)	0.717	1.192(0.440,3.224)	0.730
25OHD	Simple median	58	1.158(0.499,2.687)	0.733	1.137(0.489,2.640)	0.766
	Weighted median	58	1.470(0.712,3.035)	0.297	1.115(0.536,2.320)	0.770
	MR Egger	58	1.193(0.576,2.472)	0.637	0.801(0.401,1.600)	0.532
	IVW	58	1.112(0.674,1.835)	0.677	1.000(0.620,1.613)	0.999
Absolute lycopene	Simple median	5	0.946(0.729,1.227)	0.674	0.992(0.769,1.279)	0.948
	Weighted median	5	1.052(0.854,1.295)	0.635	0.918(0.746,1.130)	0.420
	MR Egger	5	1.331(0.964,1.839)	0.181	0.784(0.564,1.091)	0.245
	IVW	5	1.076(0.902,1.283)	0.416	0.963(0.807,1.149)	0.673
Relative ascorbate	Simple median	13	1.141(0.669,1.945)	0.628	0.862(0.482,1.544)	0.618
	Weighted median	13	0.995(0.609,1.626)	0.985	1.206(0.702,2.073)	0.498
	MR Egger	13	0.641(0.275,1.493)	0.325	2.082(0.878,4.938)	0.124
	IVW	13	1.132(0.790,1.623)	0.499	1.061(0.733,1.536)	0.753
Vitamin C	Simple median	9	1.038(0.510,2.111)	0.918	2.298(0.913,5.783)	0.077
	Weighted median	9	1.068(0.602,1.897)	0.821	0.843(0.478,1.488)	0.556
	MR Egger	9	1.314(0.515,3.355)	0.586	0.685(0.339,1.384)	0.327
	IVW	9	0.878(0.473,1.627)	0.678	1.191(0.695,2.041)	0.525
Absolute retinol	IVW	2	3.987(0.538,29.553)	0.176	0.510(0.066,3.946)	0.519
Relative retinol	Simple median	21	1.199(1.033,1.391)	0.017	0.941(0.809,1.095)	0.435
	Weighted median	21	1.033(0.886,1.205)	0.675	0.945(0.817,1.093)	0.444
	MR Egger	21	1.206(0.898,1.618)	0.228	0.990(0.745,1.317)	0.946
	IVW	21	1.054(0.944,1.177)	0.353	0.965(0.867,1.074)	0.516

FA, fractional order anisotropy; MD, mean diffusivity; MR, Mendelian randomization; SNPs, single nucleotide polymorphisms; OR, odds ratio; 95% CI, 95% confidence interval; IVW, inverse variance weighting; 25OHD: 25-hydroxyvitamin D.

Table S3. Results of the MR–Egger intercept and Cochran's Q tests for fraction anisotropy (FA) and mean diffusivity (MD).

Exposures	FA				MD			
	MR–Egger		Cochran's Q		MR–Egger		Cochran's Q	
	Intercept	*p*	Q	*p*	Intercept	*p*	Q	*p*
**Amino acids**								
Phenylalanine	0.001	0.994	1.095	0.778	0.008	0.941	1.617	0.655
Leucine	-0.035	0.881	0.037	0.982	0.131	0.614	0.037	0.982
Valine	-0.277	0.238	3.097	0.377	0.200	0.361	3.097	0.376
Tryotophan	-0.008	0.958	19.072	0.265	0.048	0.778	19.072	0.264
**Polyunsaturated fatty acids**								
Dihomogamma-linolenic acid (DGLA)	NA	NA	1.024	0.311	NA	NA	1.024	0.311
Docosahexaenoic acid (DHA)	-0.271	0.163	8.350	0.080	-0.083	0.574	1.839	0.765
Docosapentaenoic acid (DPA)	NA	NA	2.846	0.092	0.035	0.633	2.846	0.092
Gamma linolenic acid (GLA)	NA	NA	2.909	0.088	NA	NA	2.909	0.088
Arachidonic acid (AA)	NA	NA	1.010	0.315	NA	NA	1.010	0.315
Linoleic acid (LA)	-0.049	0.255	15.838	0.199	0.055	0.335	18,410	0.189
**Minerals**								
Copper	NA	NA	5.905	0.015	NA	NA	5.905	0.015
Calcium	0.008	0.527	223.249	0.003	-0.025	0.058	223.249	0.003
Iron	NA	NA	0.003	0.955	NA	NA	0.003	0.955
Phosphorus	-0.036	0.816	1.617	0.655	-0.035	0.879	1.095	0.778
Zinc	NA	NA	1.736	0.188	NA	NA	1.736	0.188
Magnesium	0.032	0.607	2.163	0.826	-0.050	0.438	2.162	0.826
**Vitamins**								
Absolute α-tocopherol	NA	NA	0.834	0.361	NA	NA	0.834	0.361
Relative α-tocopherol	-0.005	0.915	4.556	0.804	0.008	0.870	4.556	0.804
Relative γ-tocopherol	-0.006	0.873	4.207	0.938	0.011	0.806	4.206	0.938
25OHD	-0.003	0.796	73.629	0.068	0.009	0.388	73.629	0.068
Absolute lycopene	-0.092	0.224	4.134	0.388	0.088	0.246	4.134	0.388
Relative ascorbate	0.050	0.173	5.503	0.939	-0.060	0.119	5.503	0.939
Vitamin C	-0.041	0.304	14.555	0.024	0.055	0.082	15.708	0.047
Absolute retinol	NA	NA	0.325	0.569	NA	NA	0.324	0.569
Relative retinol	-0.035	0.346	22.186	0.330	-0.007	0.851	22.186	0.330

FA, fractional order anisotropy; MD, mean diffusivity; 25OHD, 25-hydroxyvitamin D.

2. Supplementary Figures

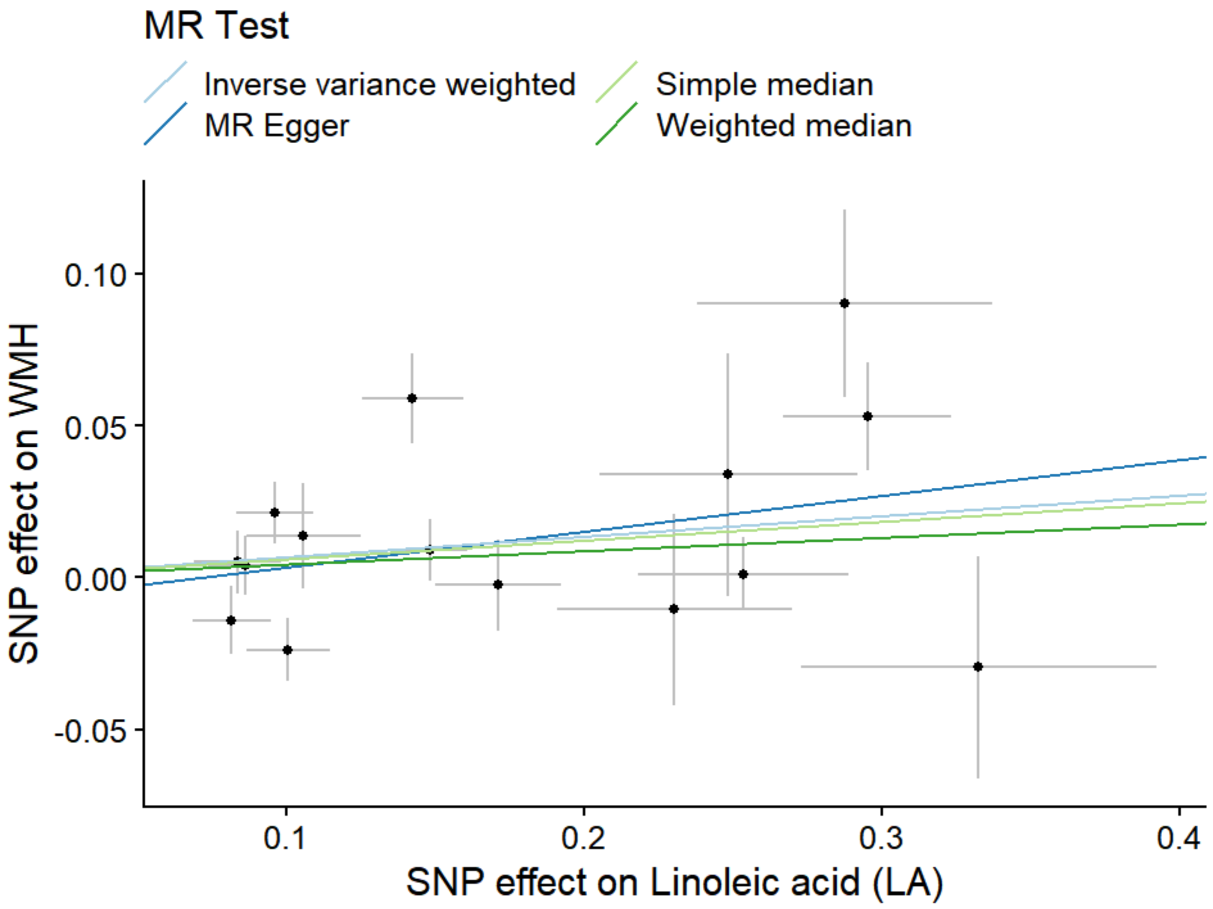

Figure S1.1 Scatterplot of Linolenic acid (LA) and WMH

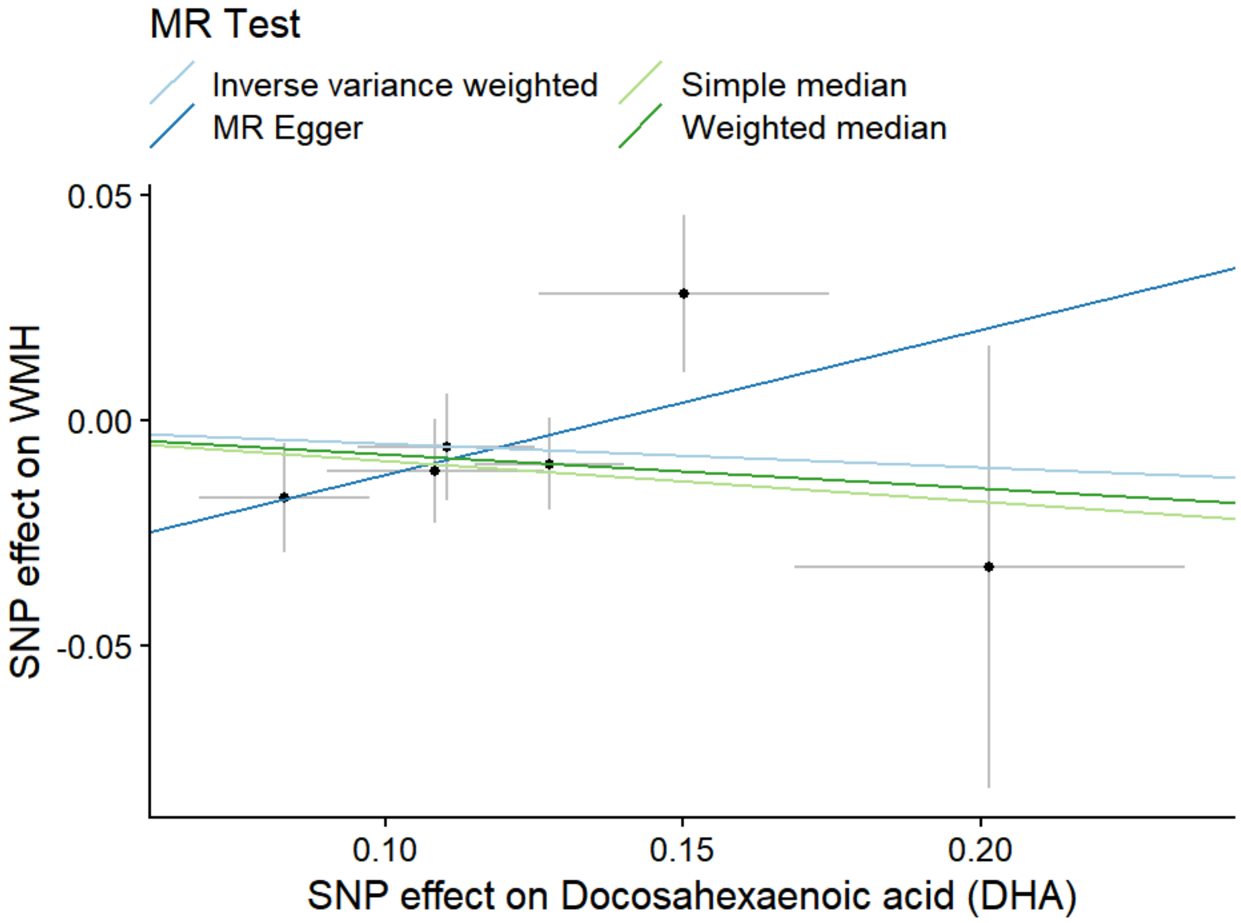

Figure S1.2 Scatterplot of docosahexaenoic acid (DHA) and WMH

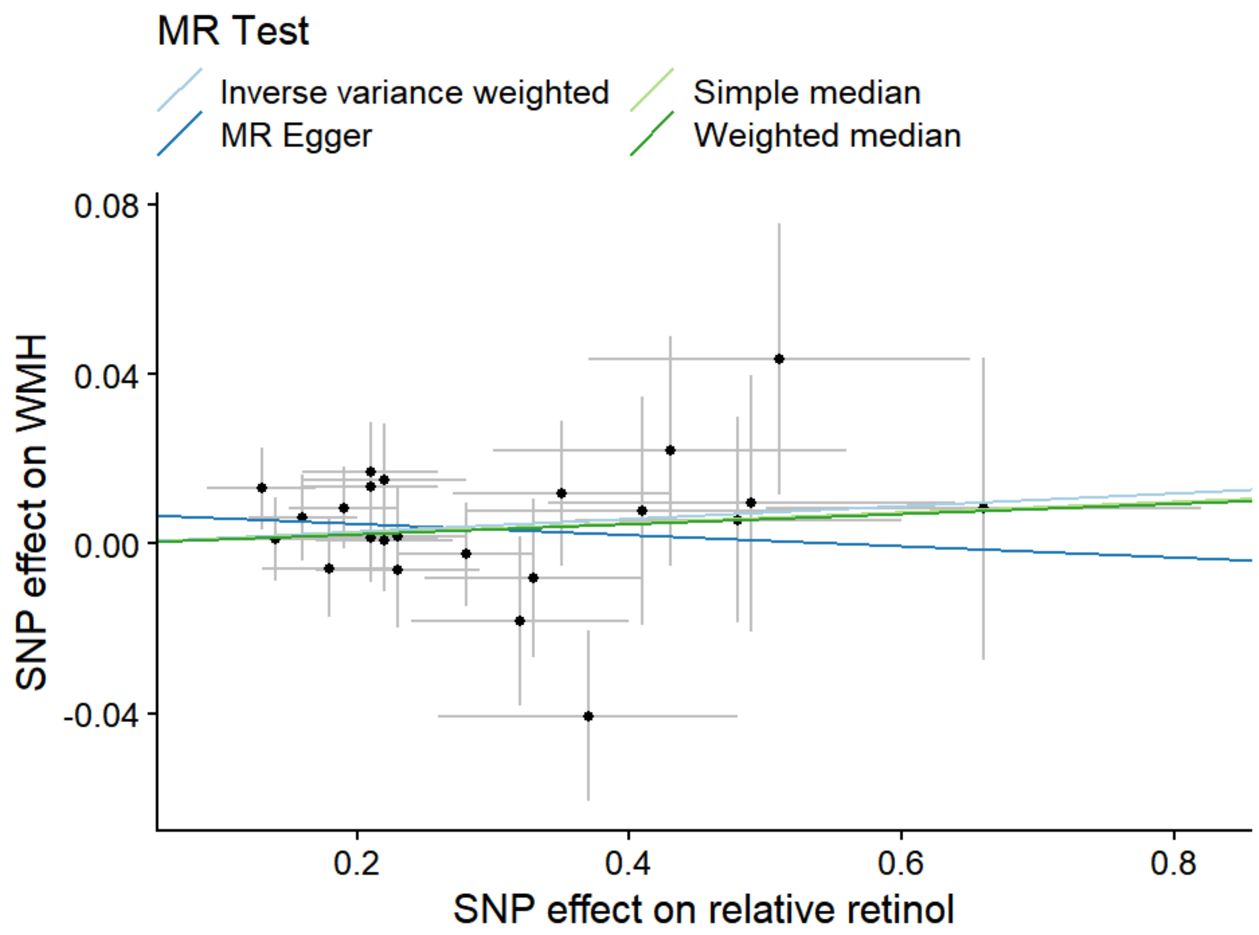

Figure S1.3 Scatterplot of relative retinol and WMH

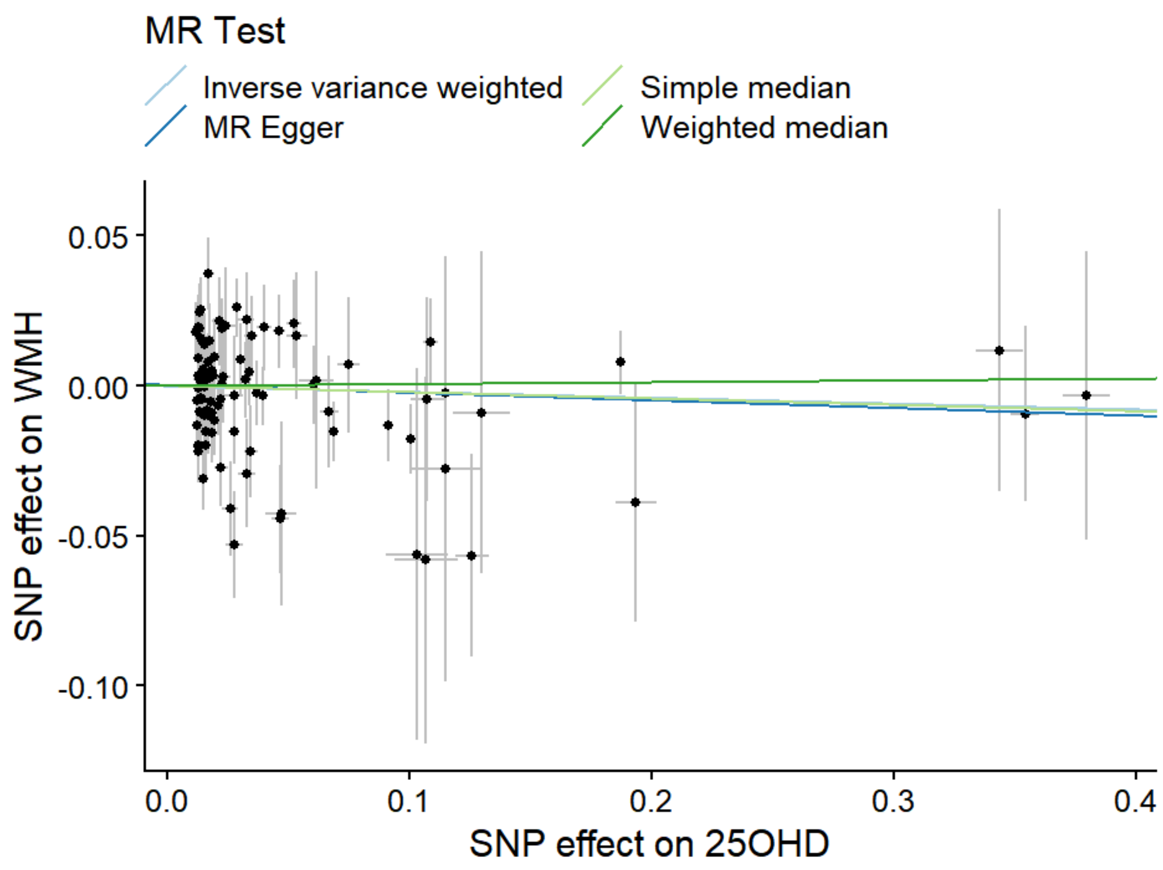

Figure S1.4 Scatterplot of 25OHD and WMH

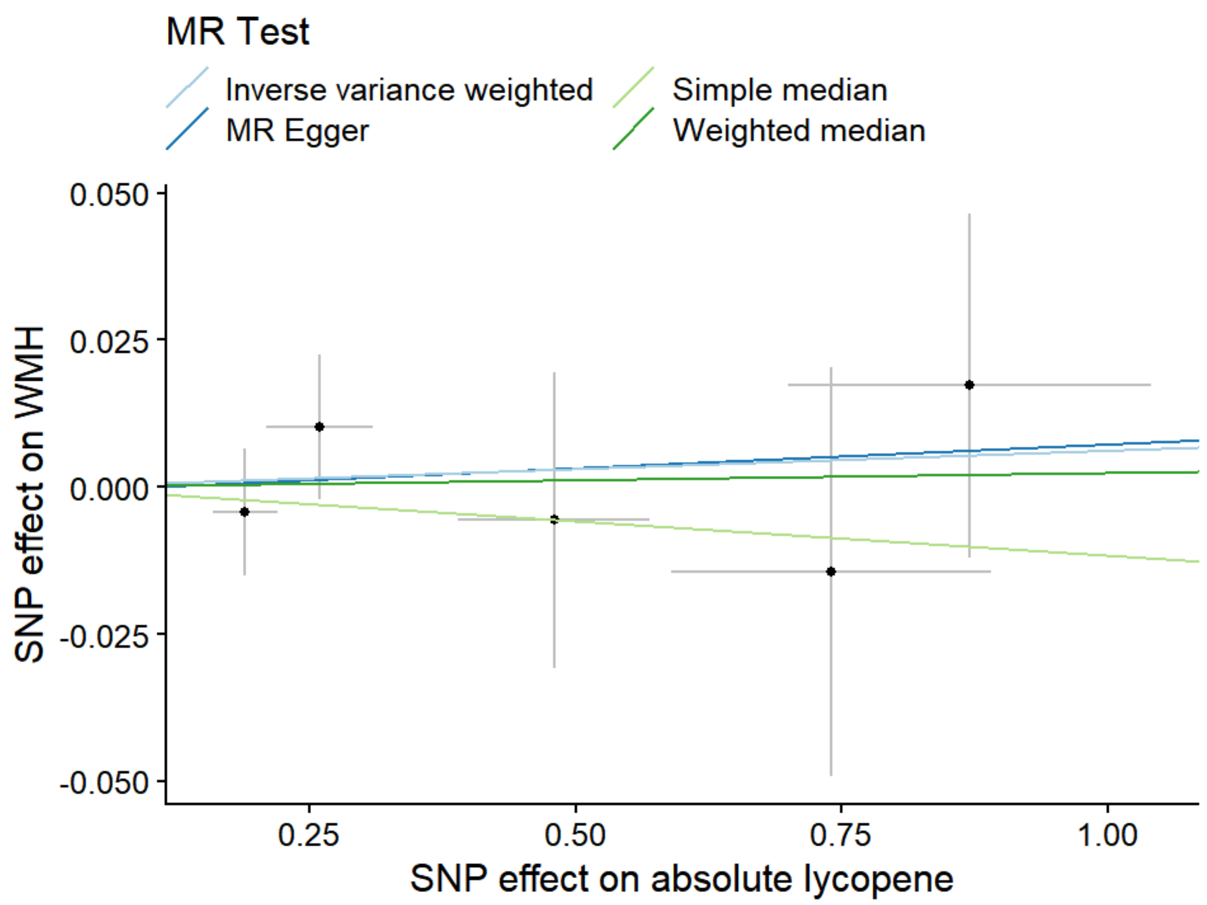

Figure S1.5 Scatterplot of absolute lycopene and WMH

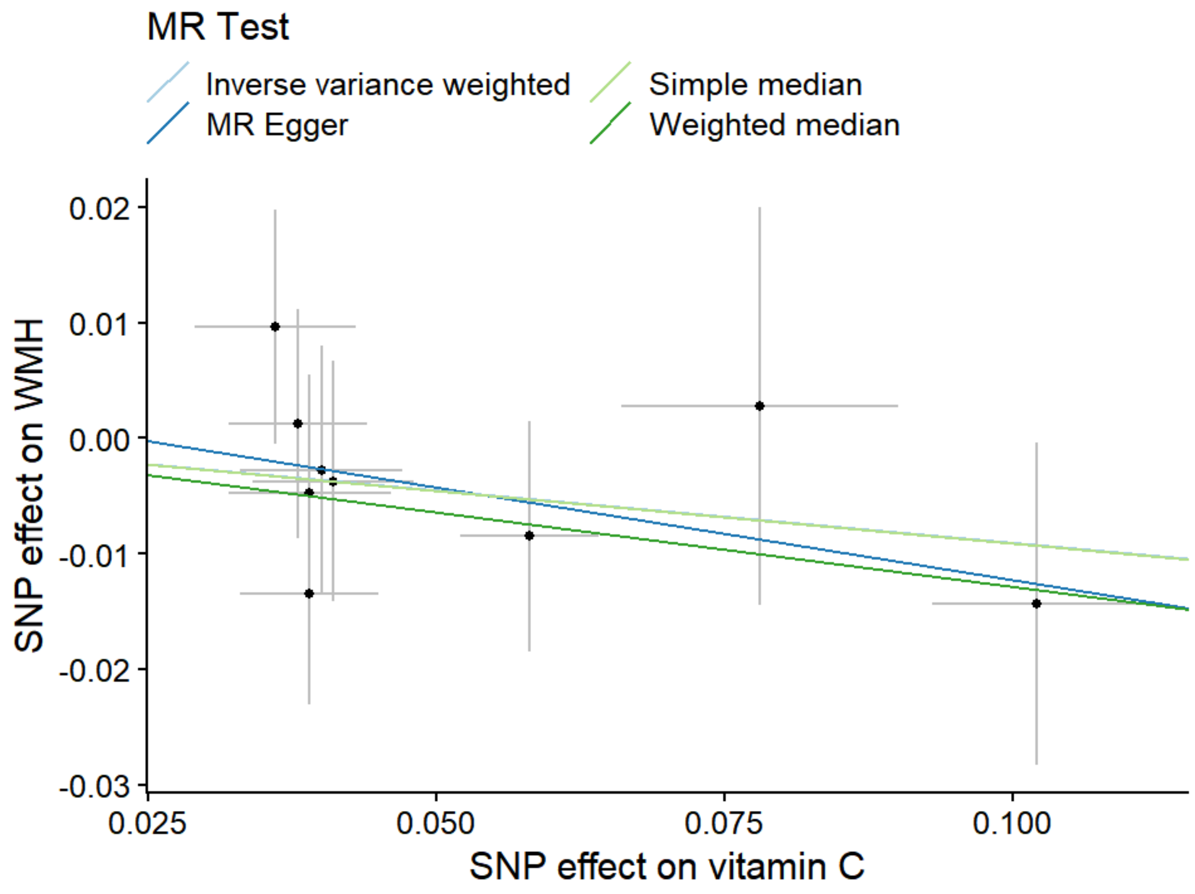

Figure S1.6 Scatterplot of vitamin C and WMH

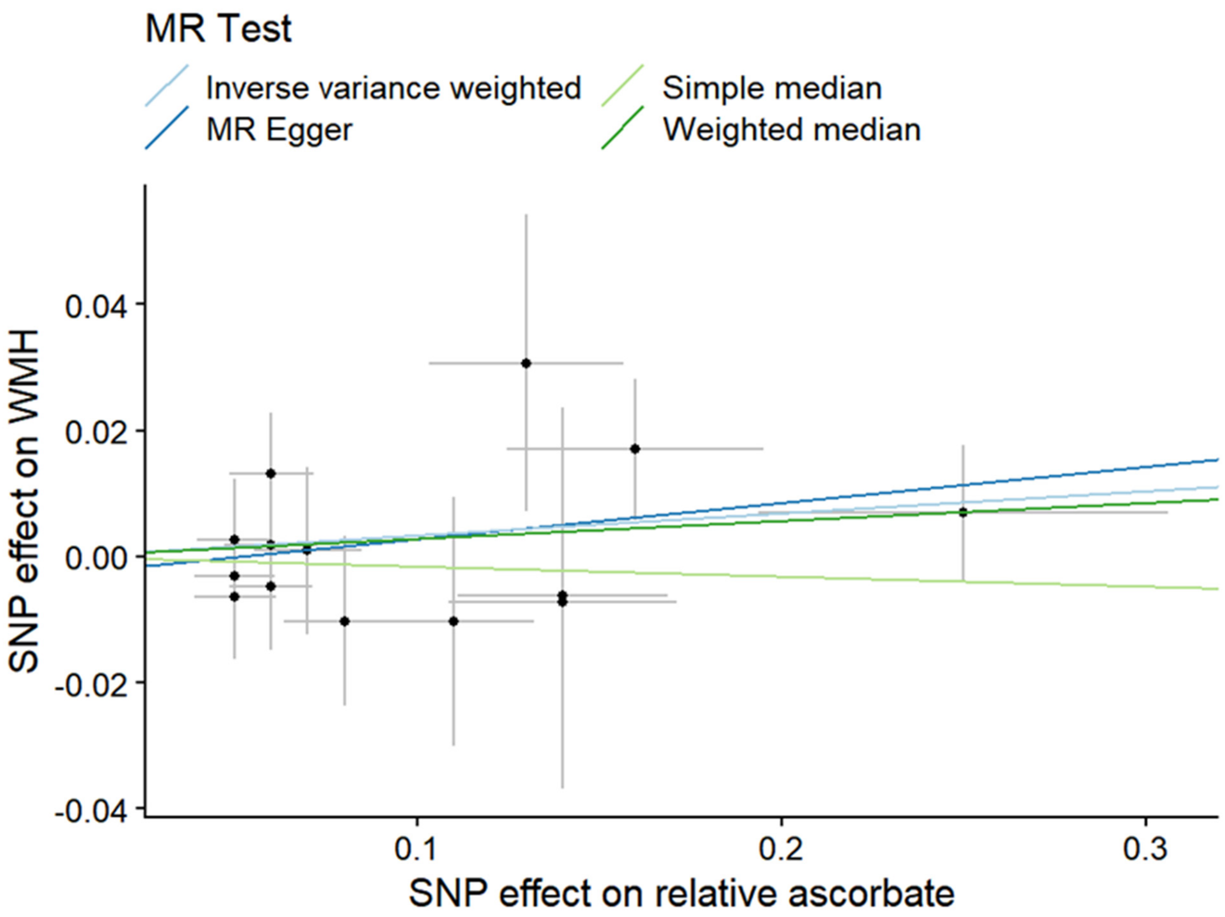

Figure S1.7 Scatterplot of relative ascorbate and WMH

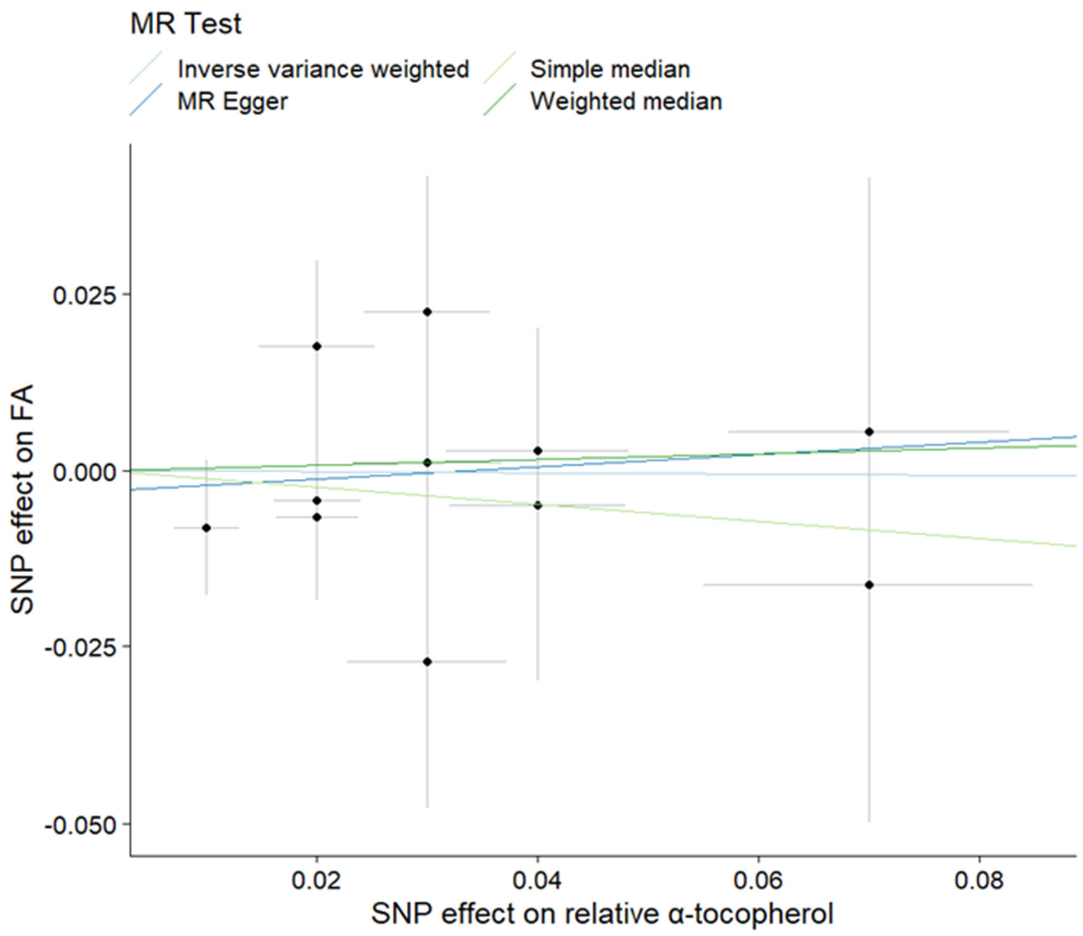

Figure S1.8 Scatterplot of relative α-tocopherol and WMH

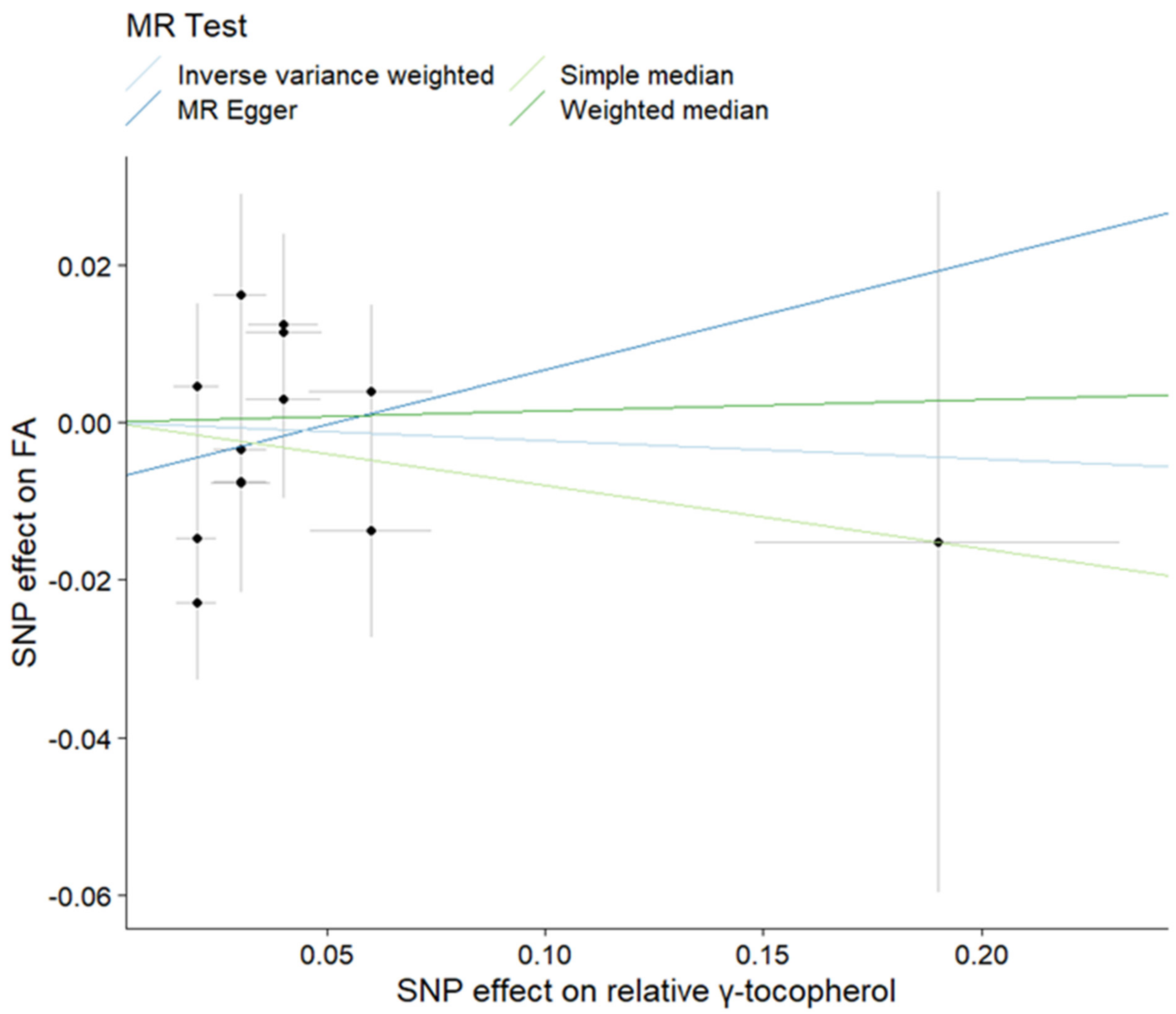

Figure S1.9 Scatterplot of relative γ -tocopherol and WMH

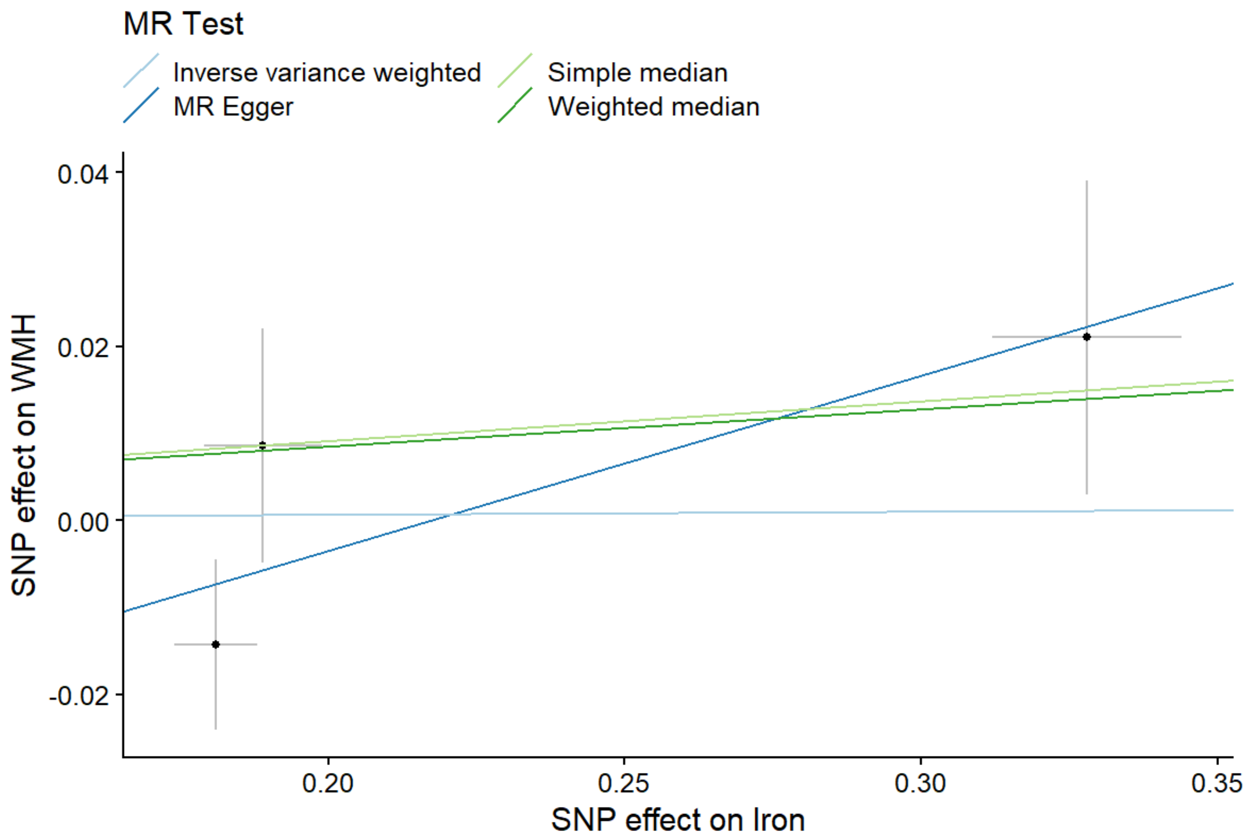

Figure S1.10 Scatterplot of iron and WMH

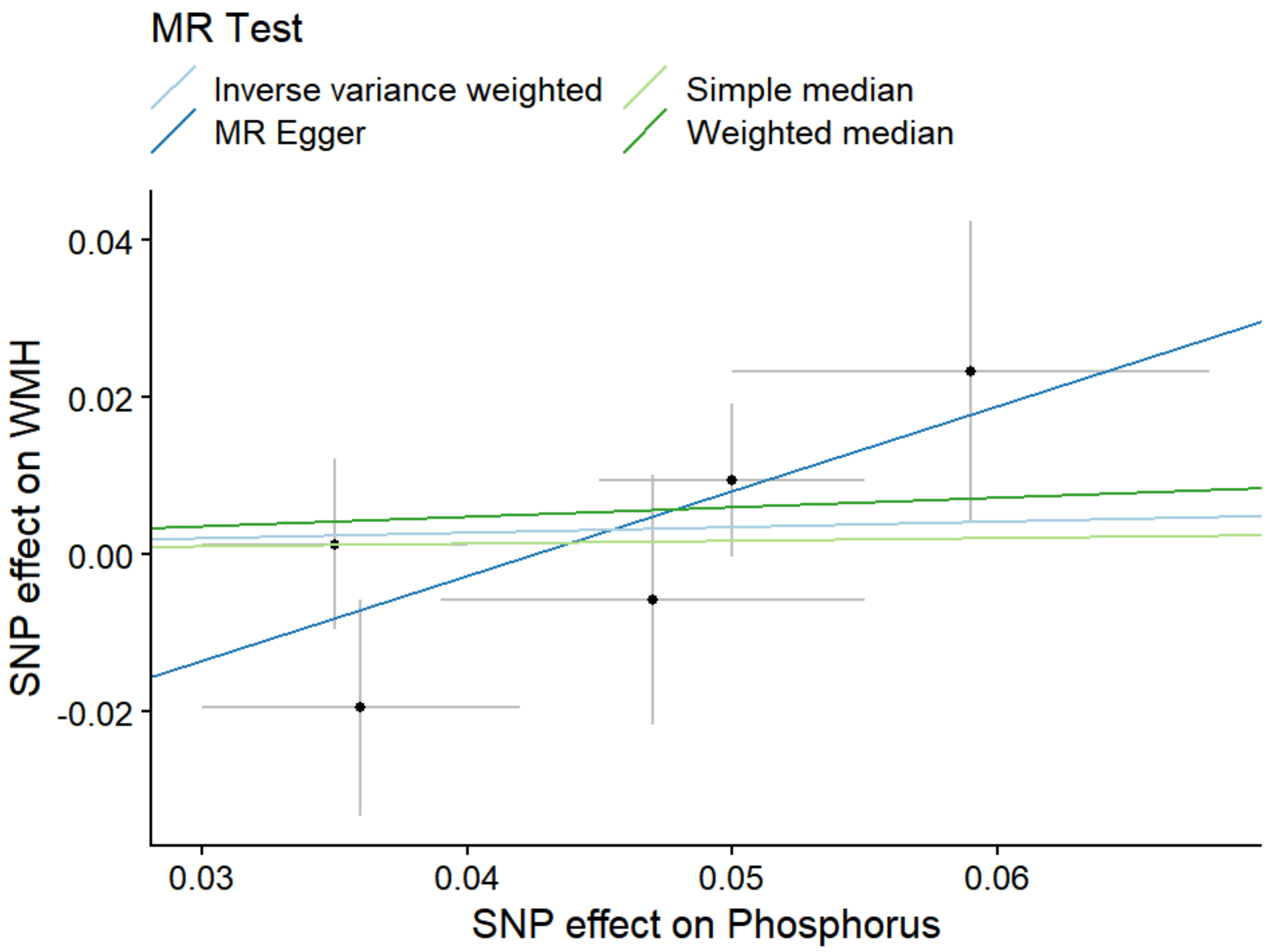

Figure S1.11 Scatterplot of phosphorus and WMH

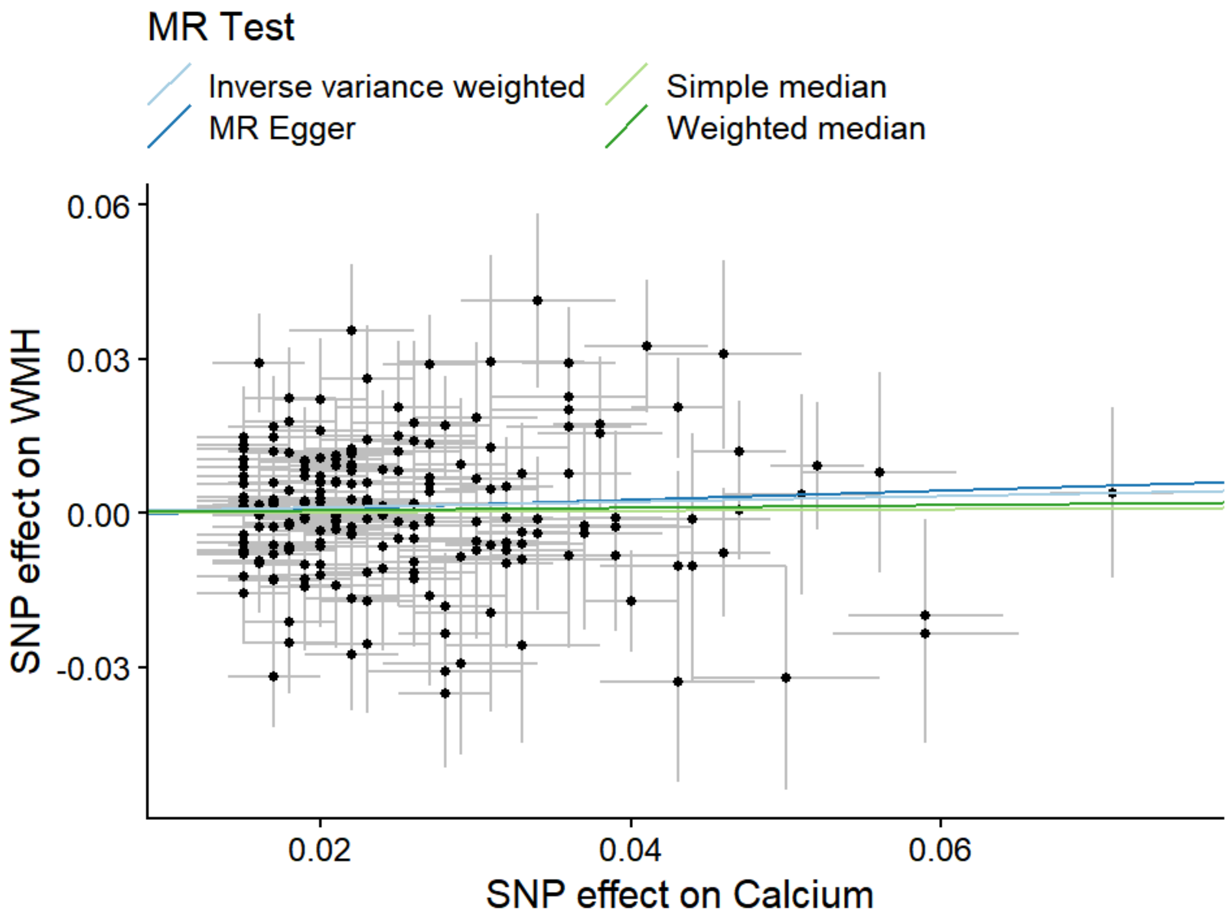

Figure S1.12 Scatterplot of calcium and WMH

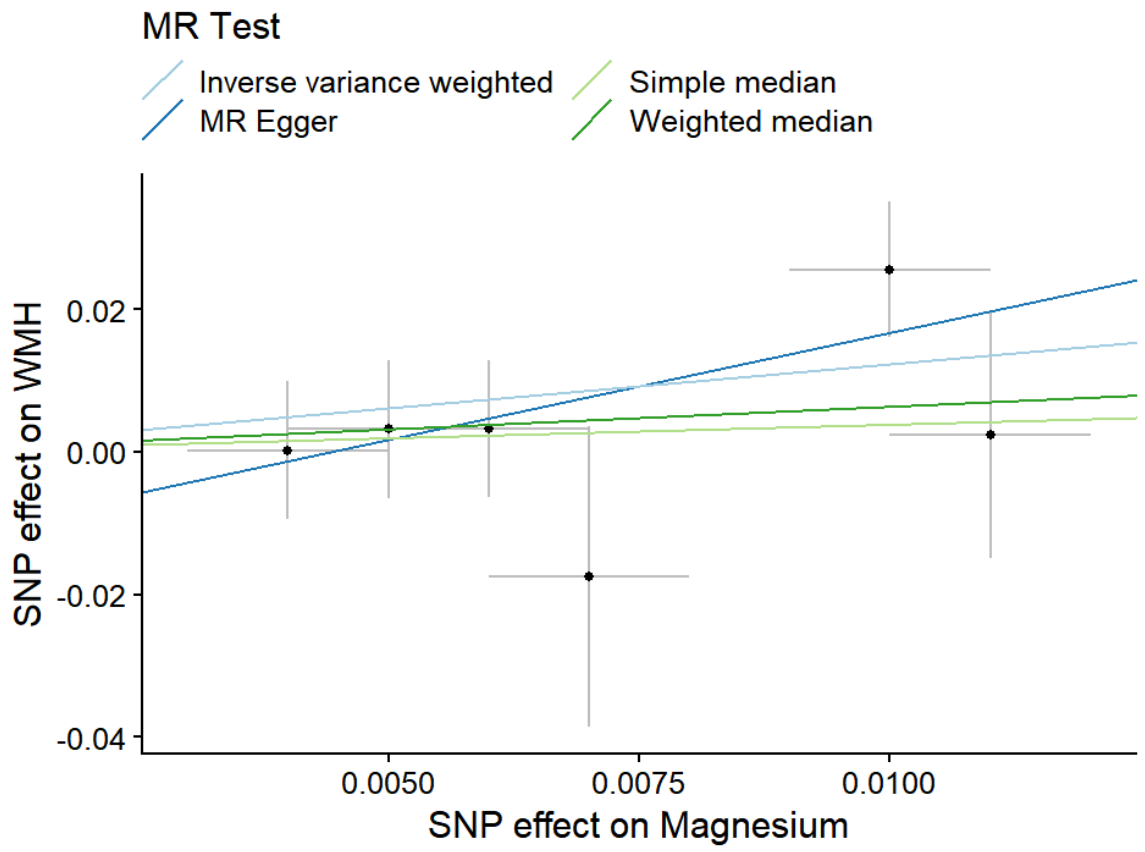

Figure S1.13 Scatterplot of magnesium and WMH

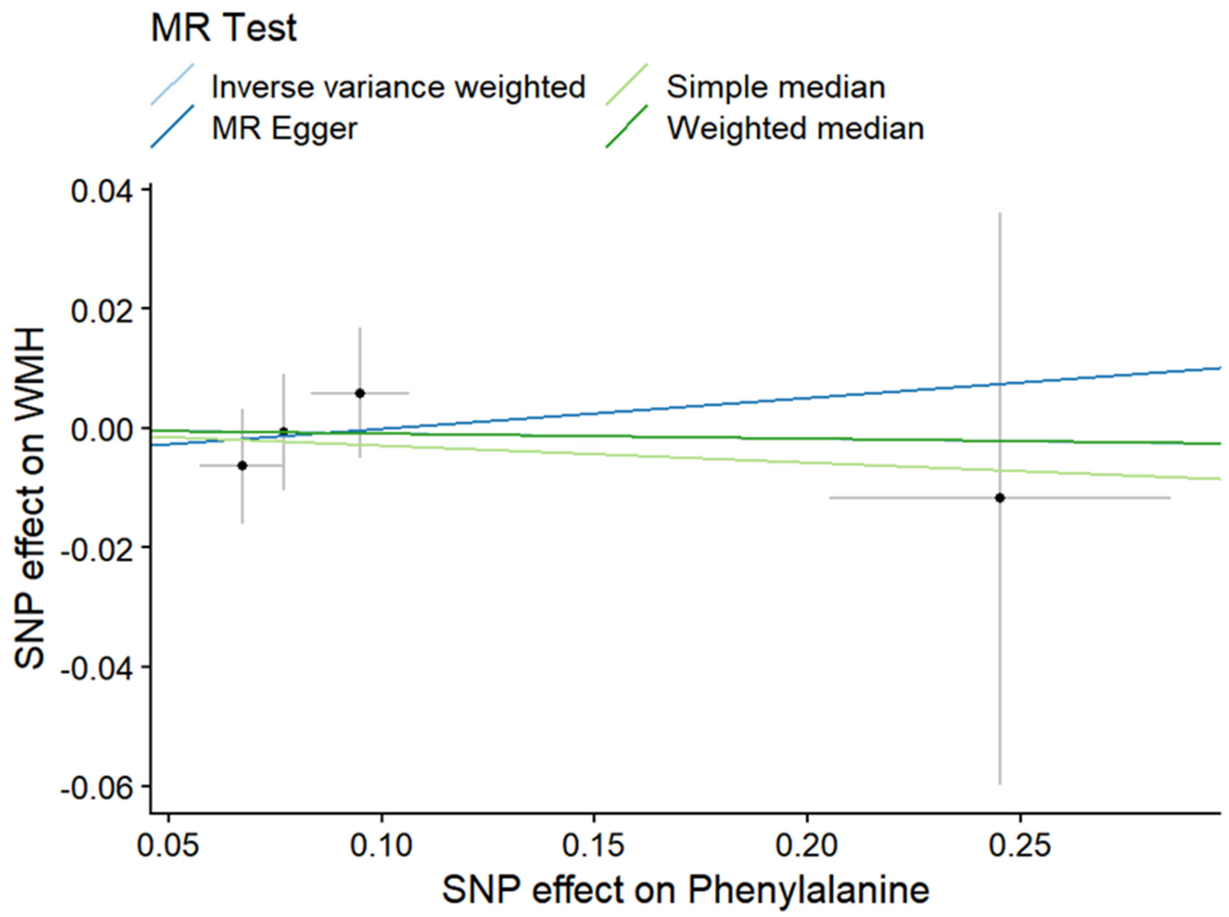

Figure S1.14 Scatterplot of phenylalanine and WMH

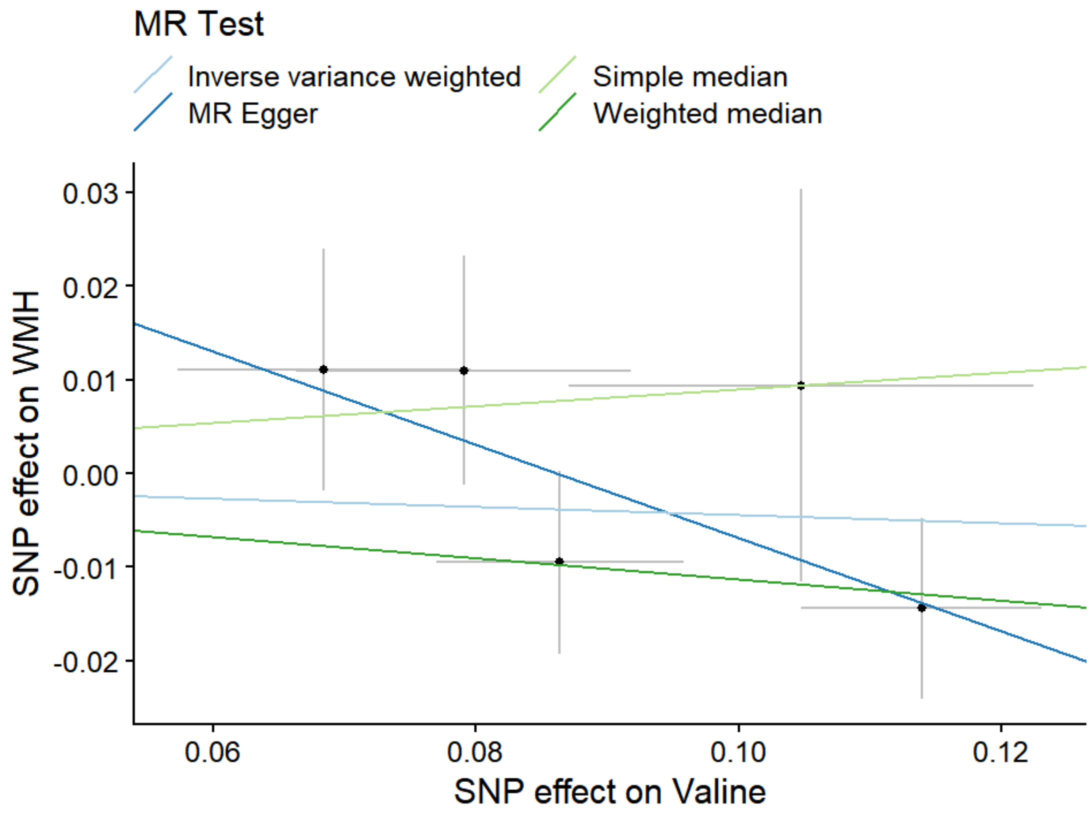

Figure S1.15 Scatterplot of valine and WMH

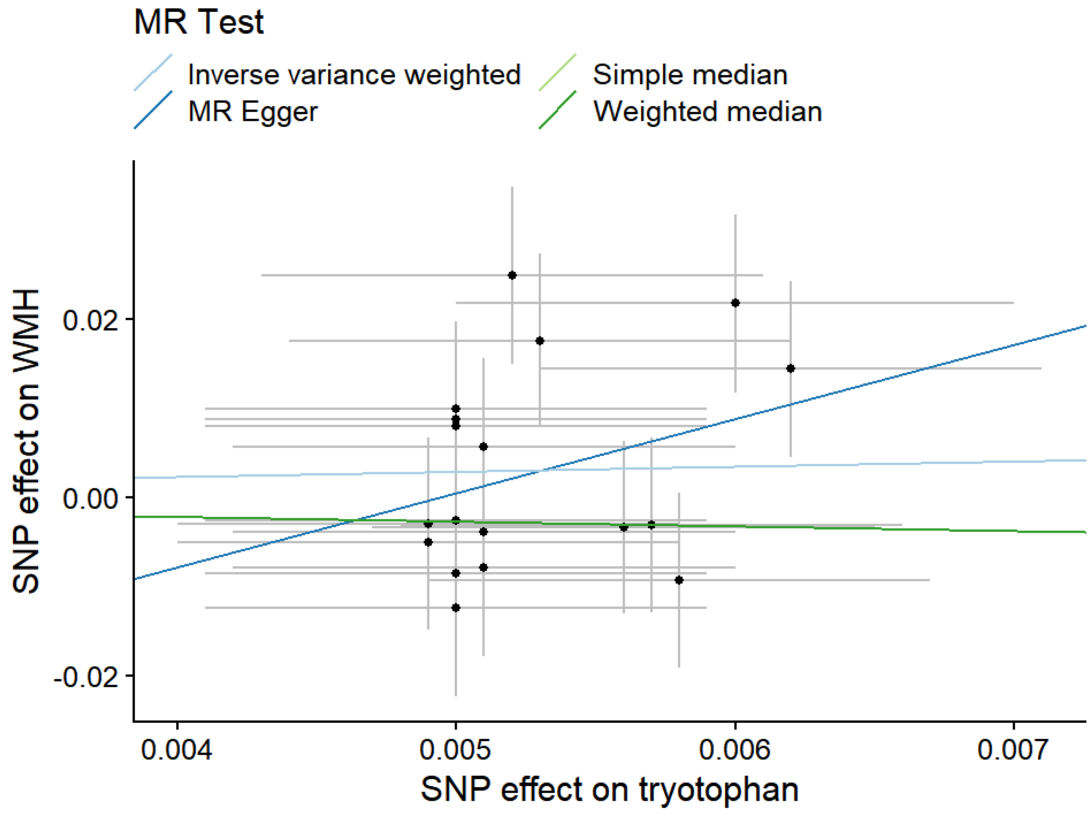

Figure S1.16 Scatterplot of tryotophan and WMH

Figure S1. Scatterplots of essential nutrients exposures and WMH. The x-axis represents the previously published β-estimate for the association between each SNP and essential nutrients. The y-axis represents the β-estimate for the association between each SNP and risk of cerebral hemorrhage. The slope of each line corresponds to the estimated MR effect per method.

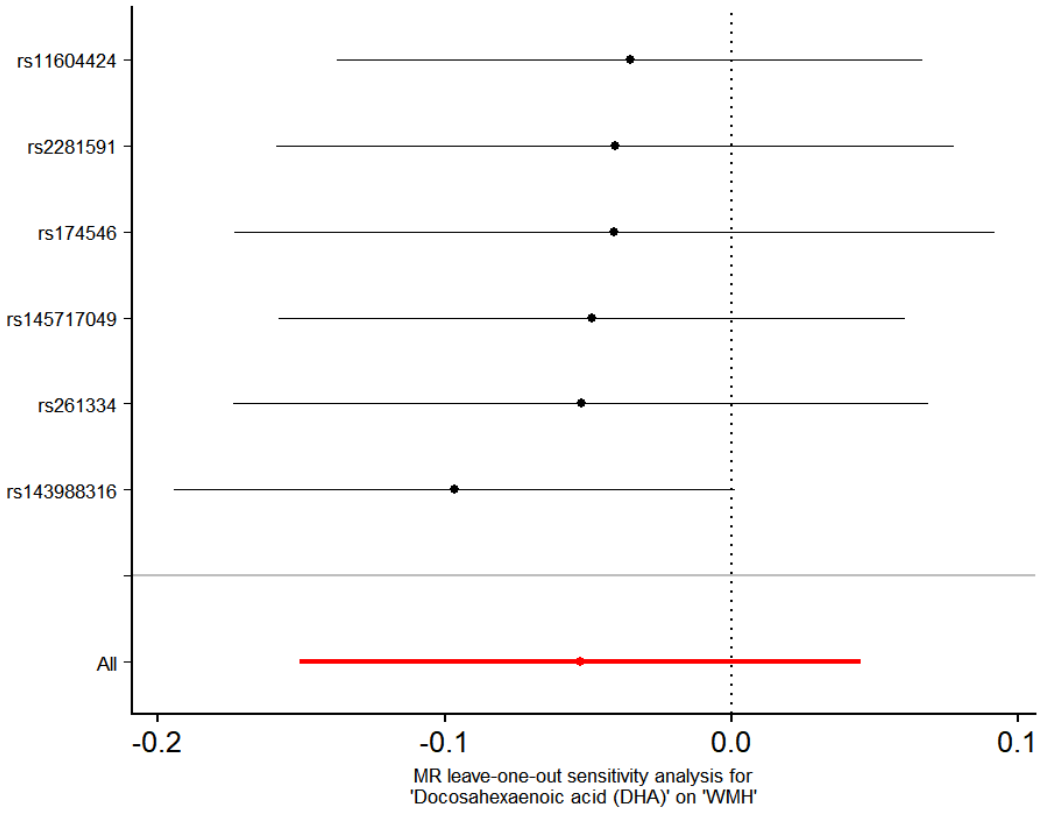

Figure S2.1 Leave-one-out plot of docosahexaenoic acid(DHA)and WMH

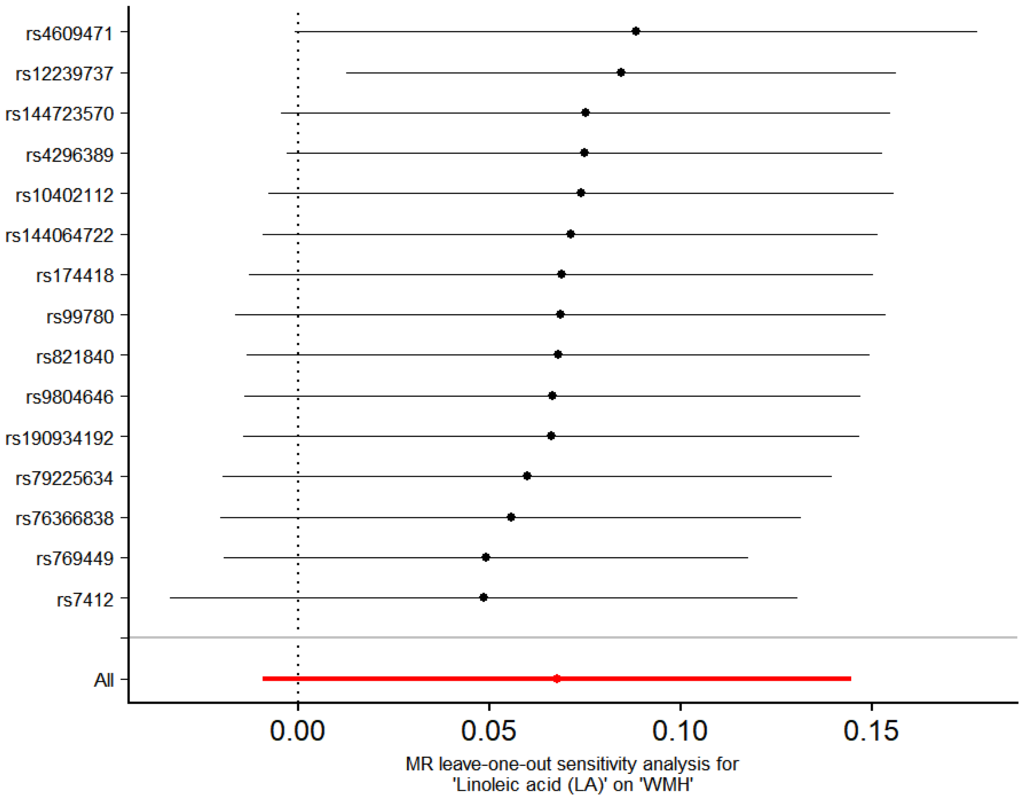

Figure S2.2 Leave-one-out plot of linolenic acid and WMH

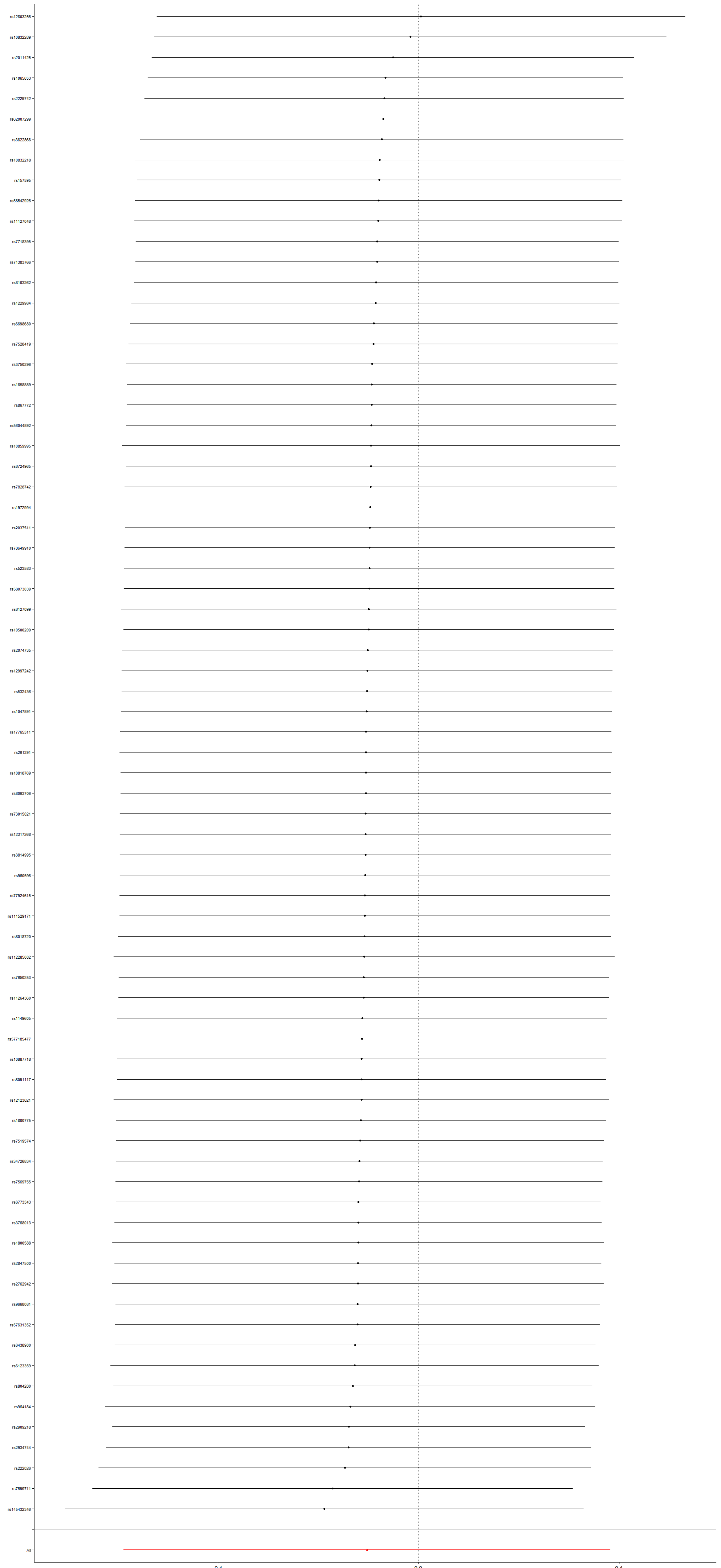

Figure S2.3 Leave-one-out plot of 25OHD and WMH

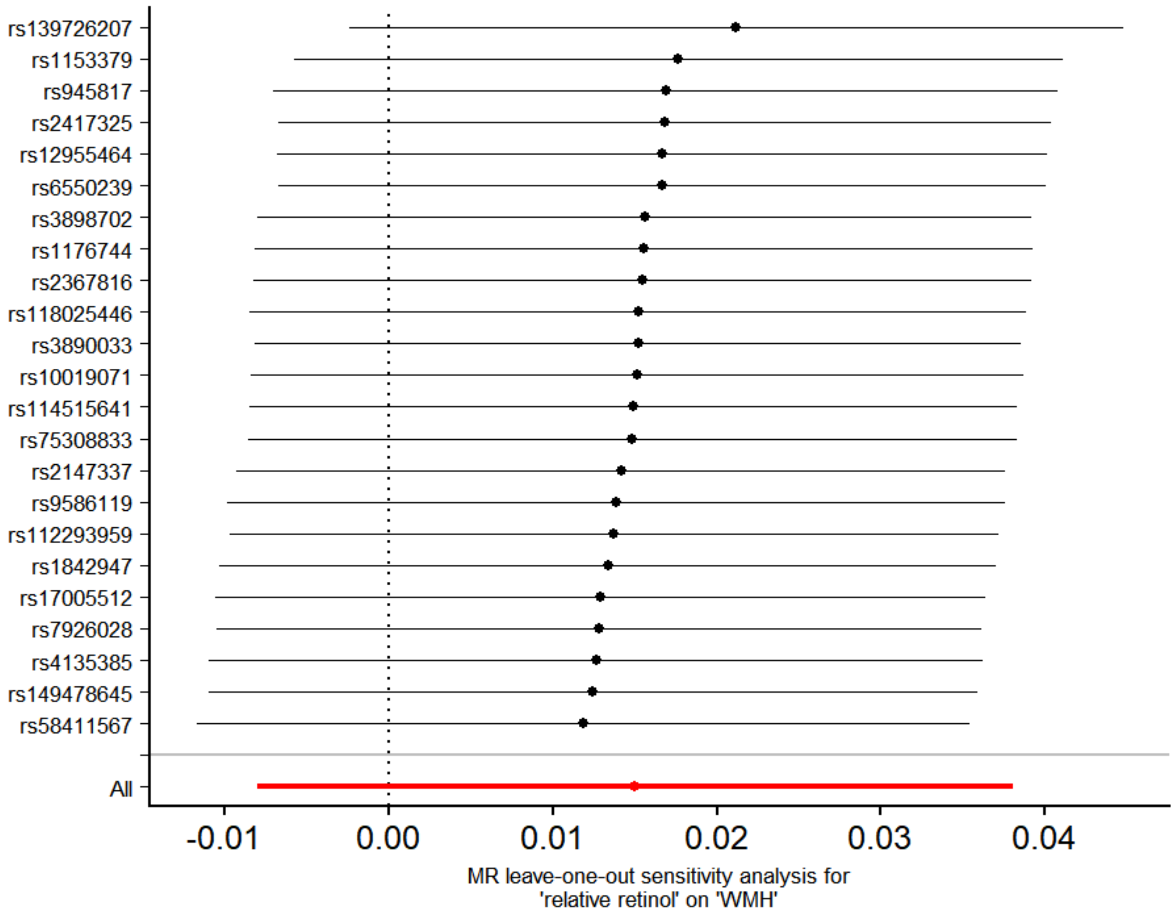

Figure S2.4 Leave-one-out plot of relative retinol and WMH

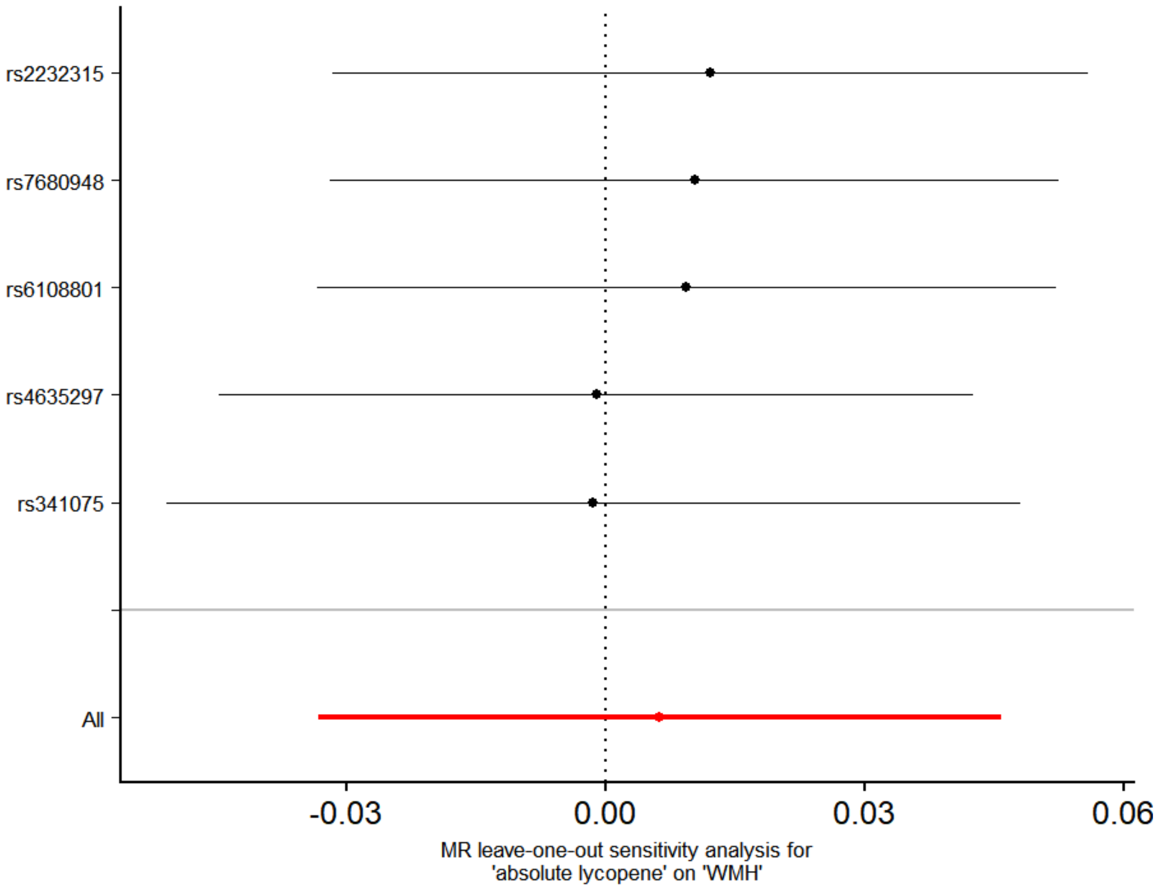

Figure S2.5 Leave-one-out plot of absolute lycopene and WMH

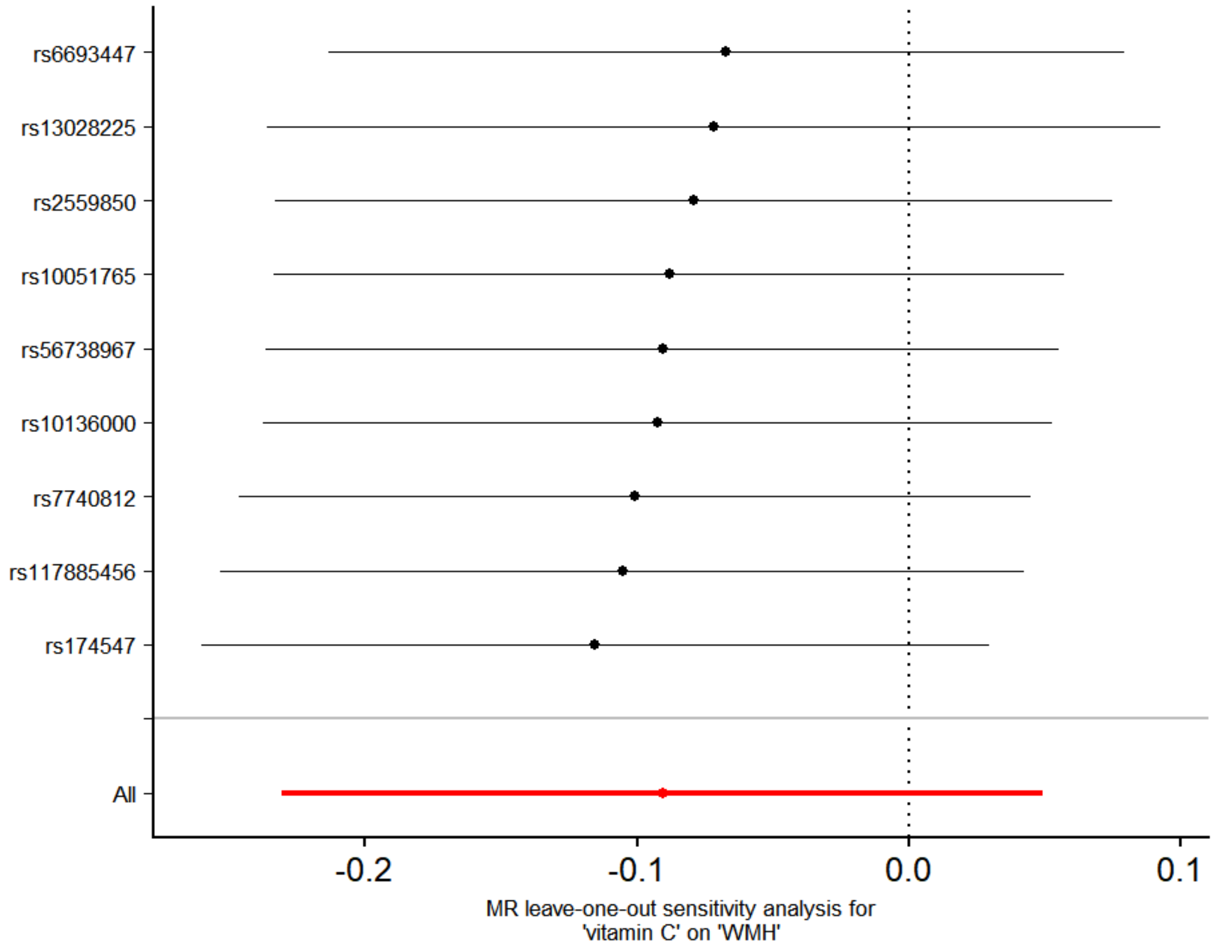

Figure S2.6 Leave-one-out plot OF vitamin C and WMH

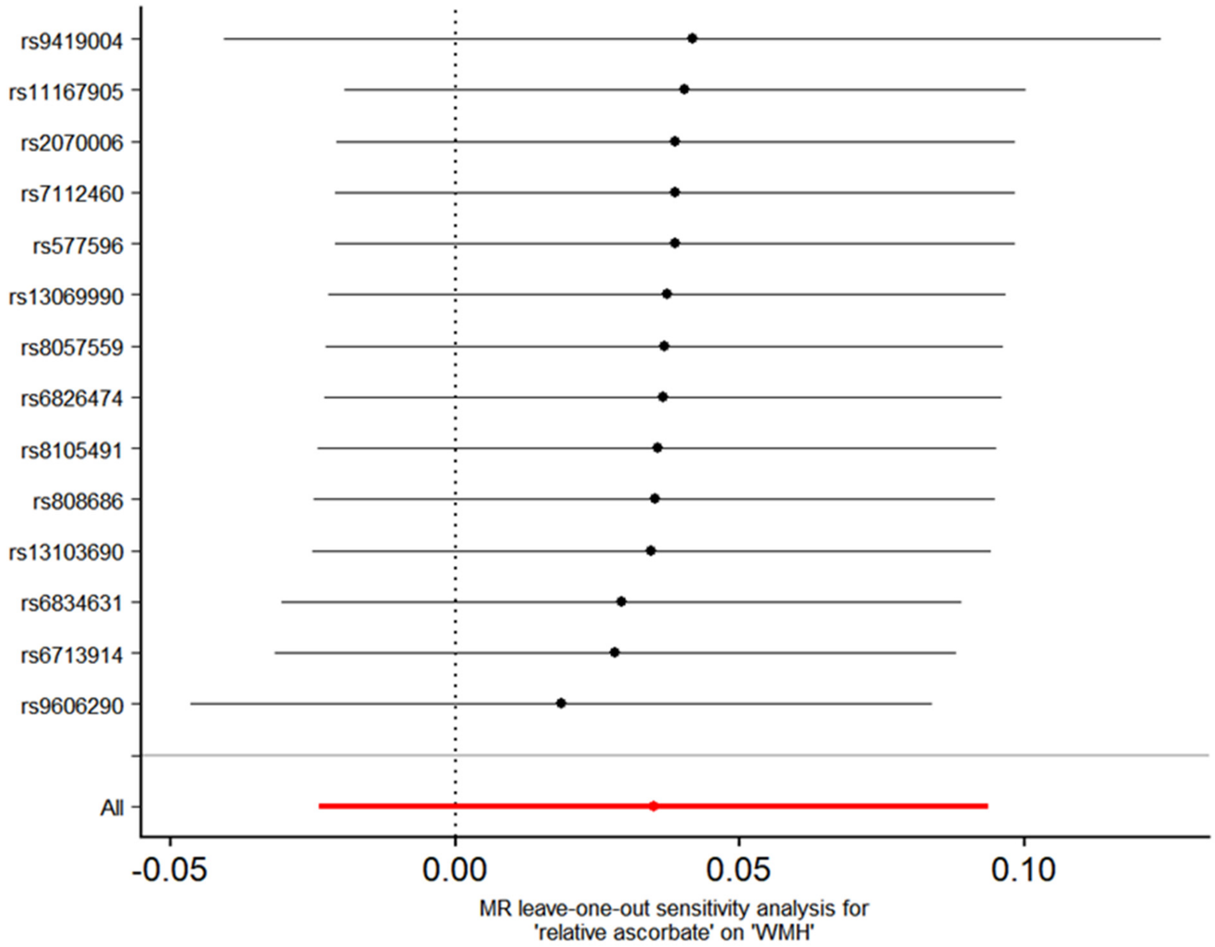

Figure S2.7 Leave-one-out plot OF relative ascorbate and WMH

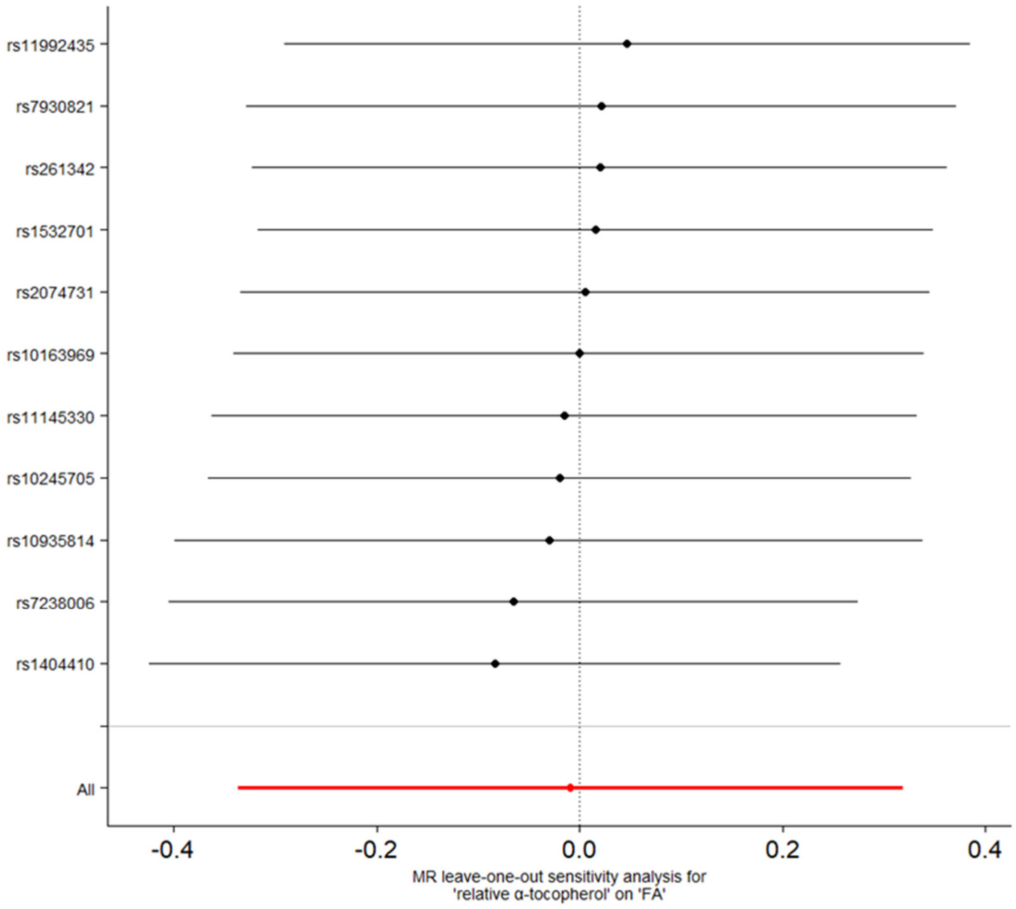

Figure S2.8 Leave-one-out plot of relative α -tocopherol and WMH

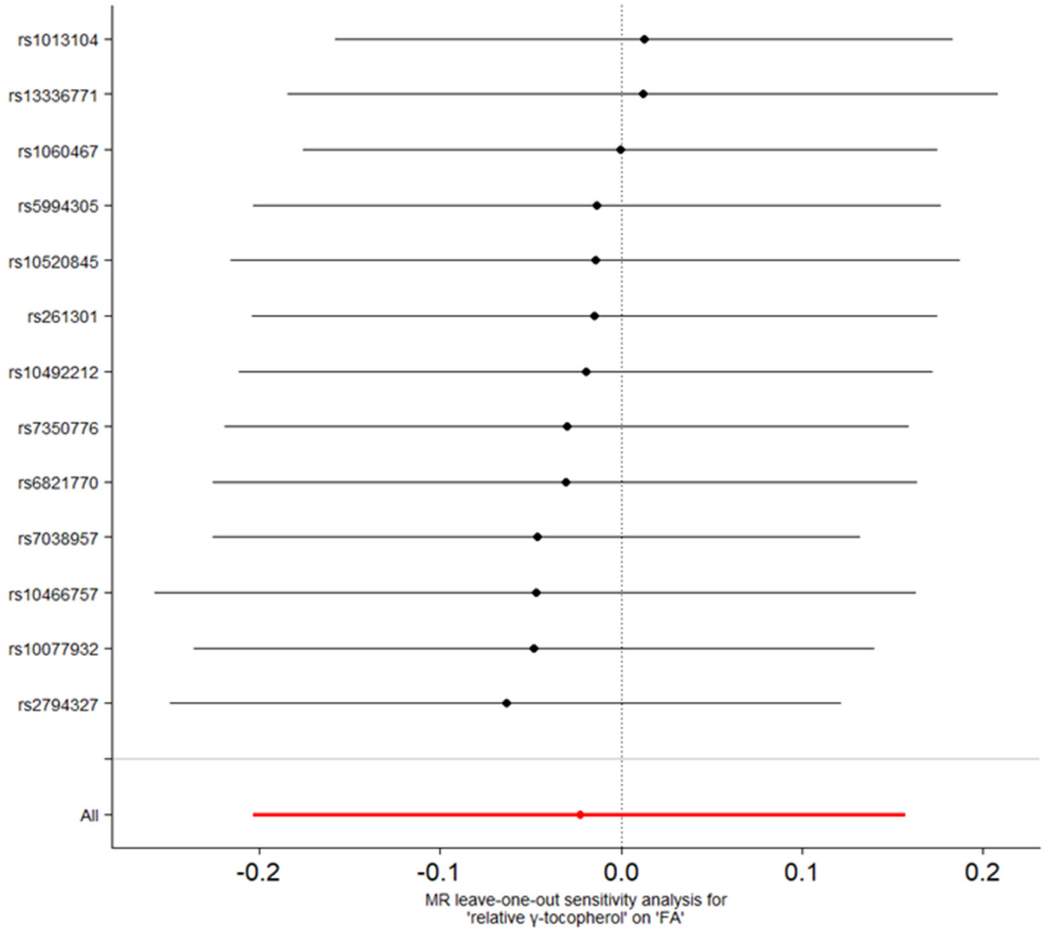

Figure S2.9 Leave-one-out plot of relative γ -tocopherol and WMH

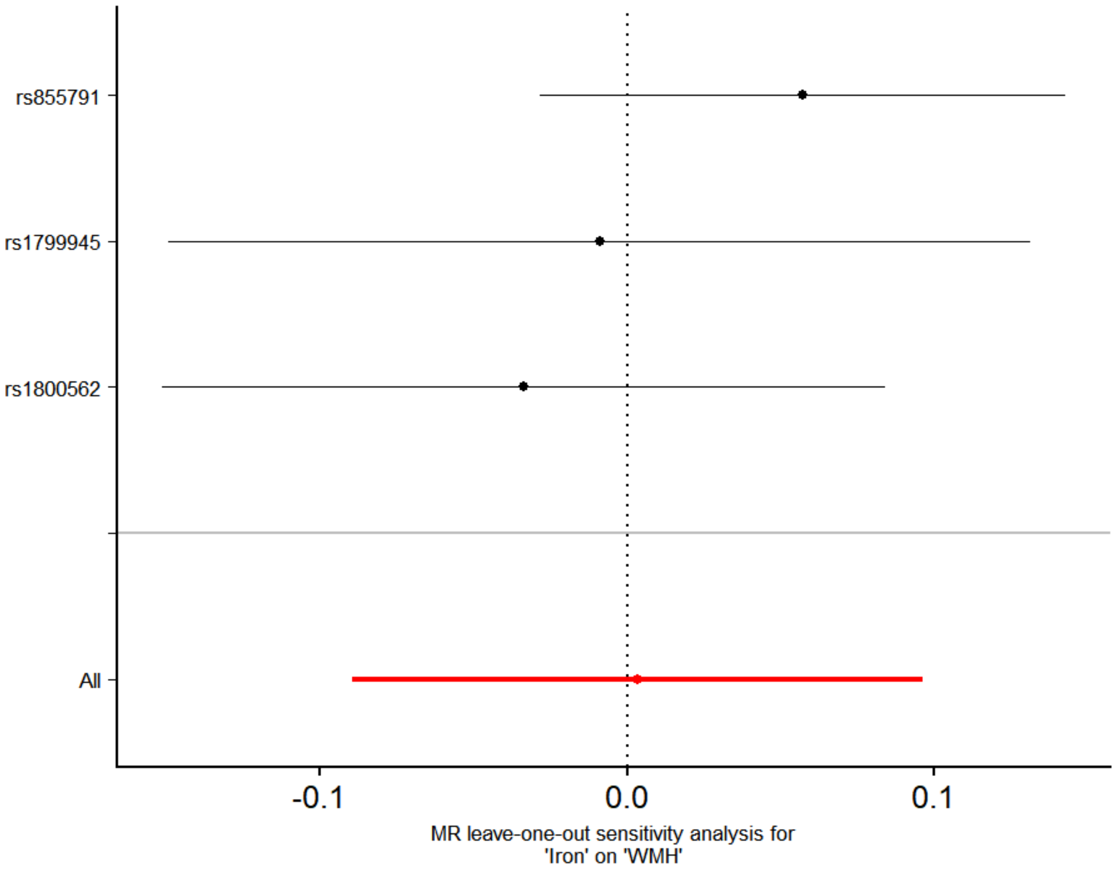

Figure S2.10 Leave-one-out plot of iron and WMH

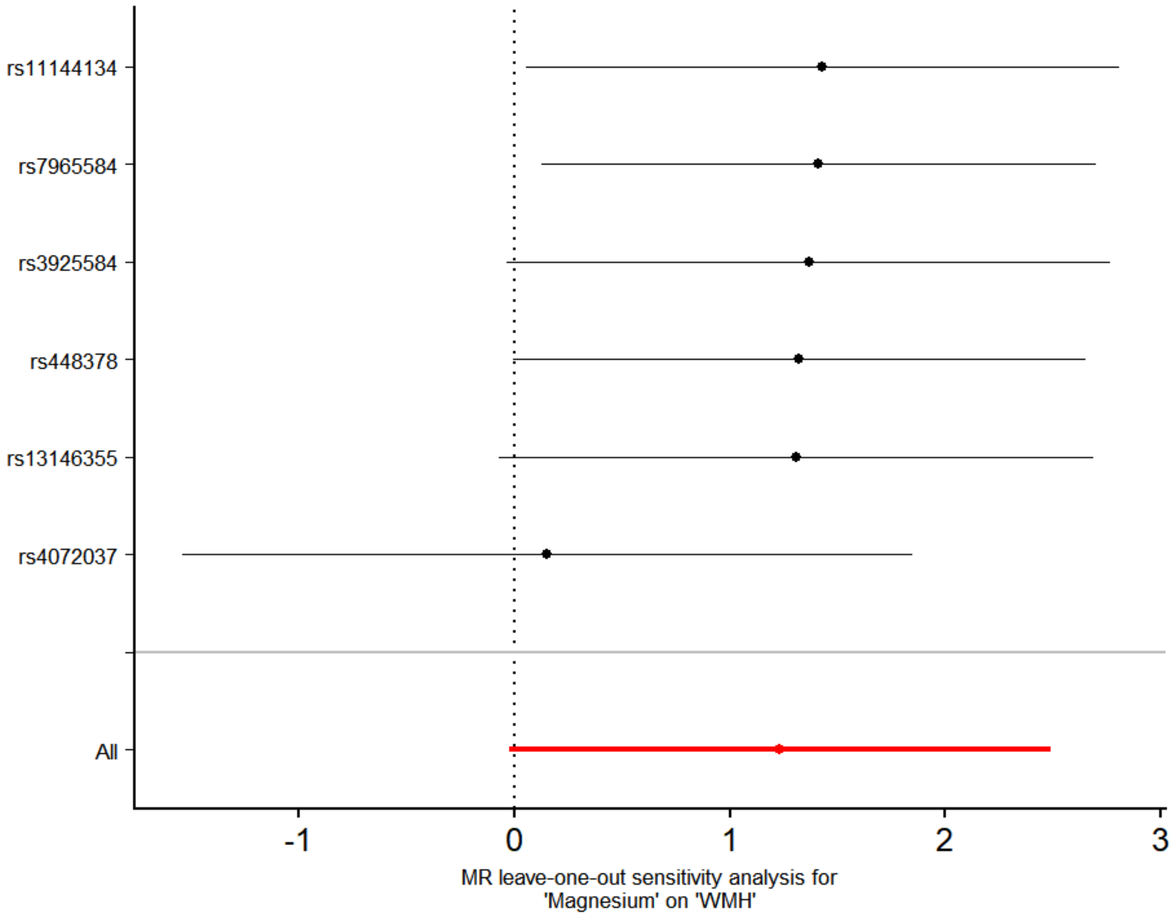

Figure S2.11 Leave-one-out plot of magnesium and WMH

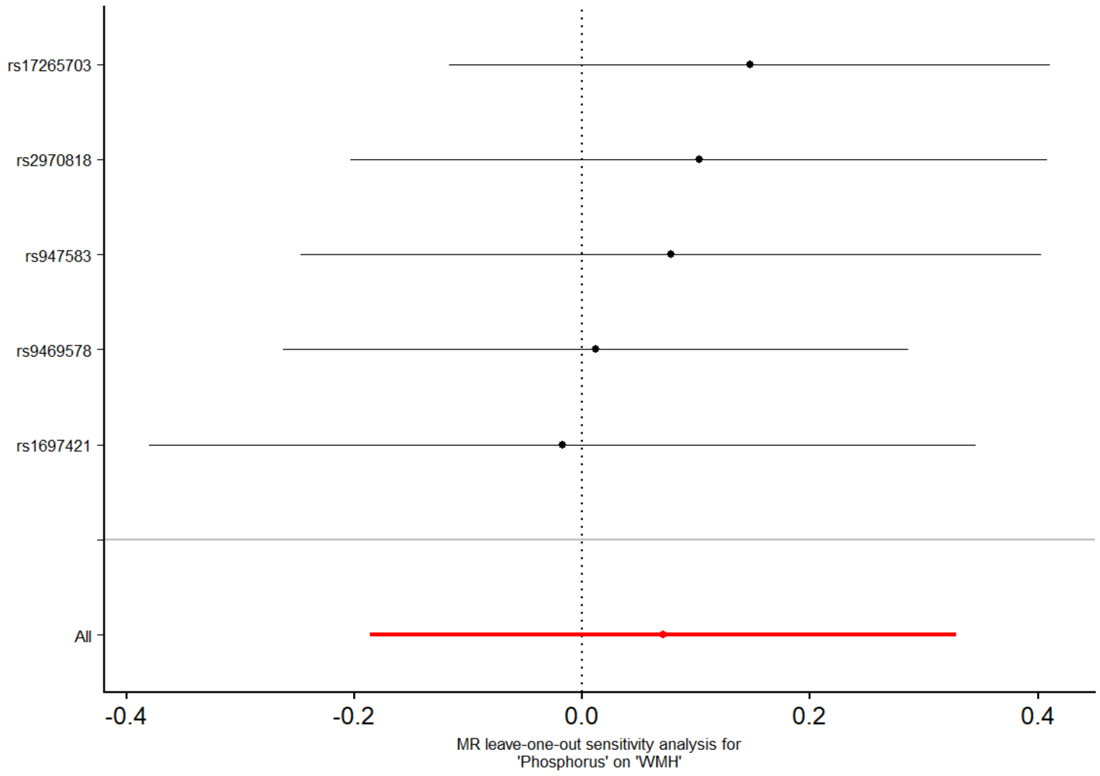

Figure S2.12 Leave-one-out plot of phosphorus and WMH

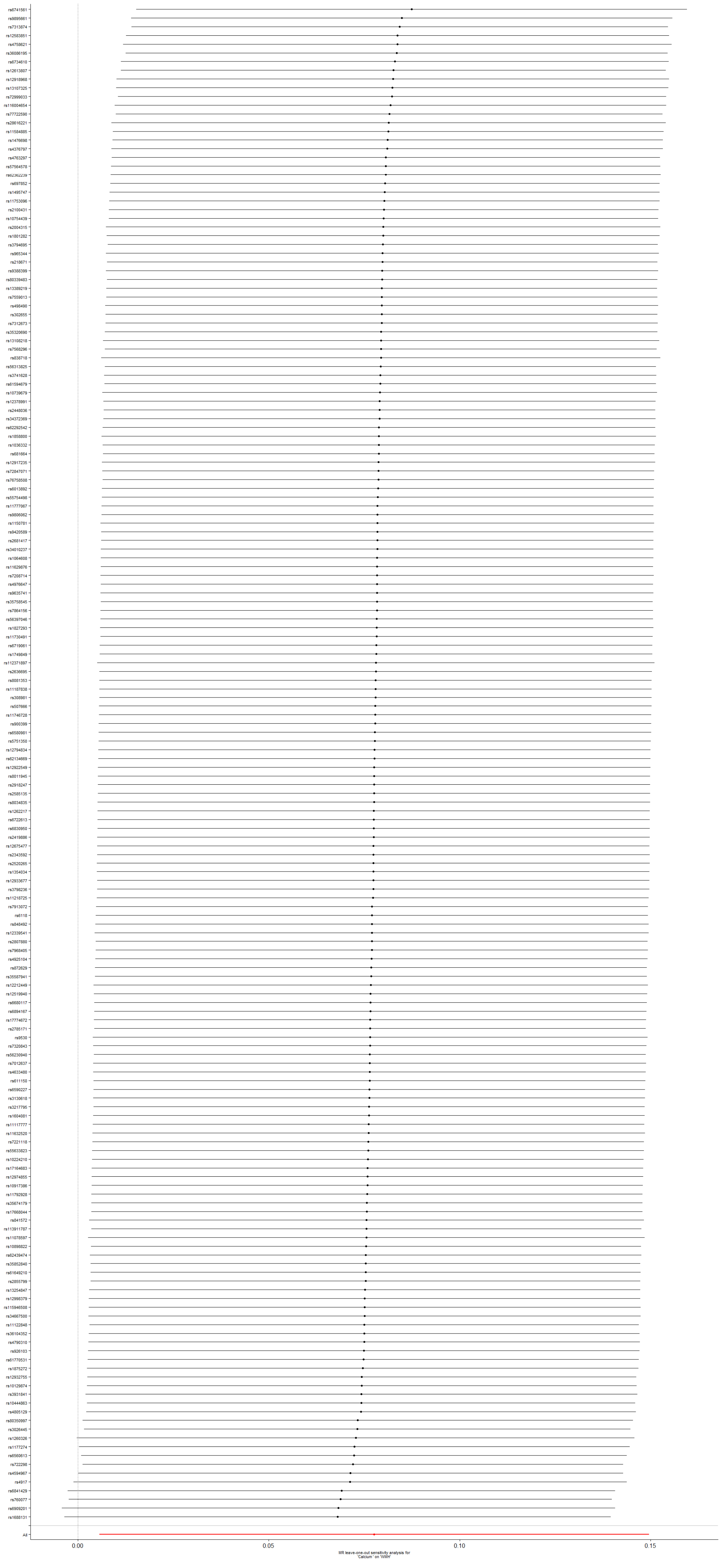

Figure S2.13 Leave-one-out plot of calcium and WMH

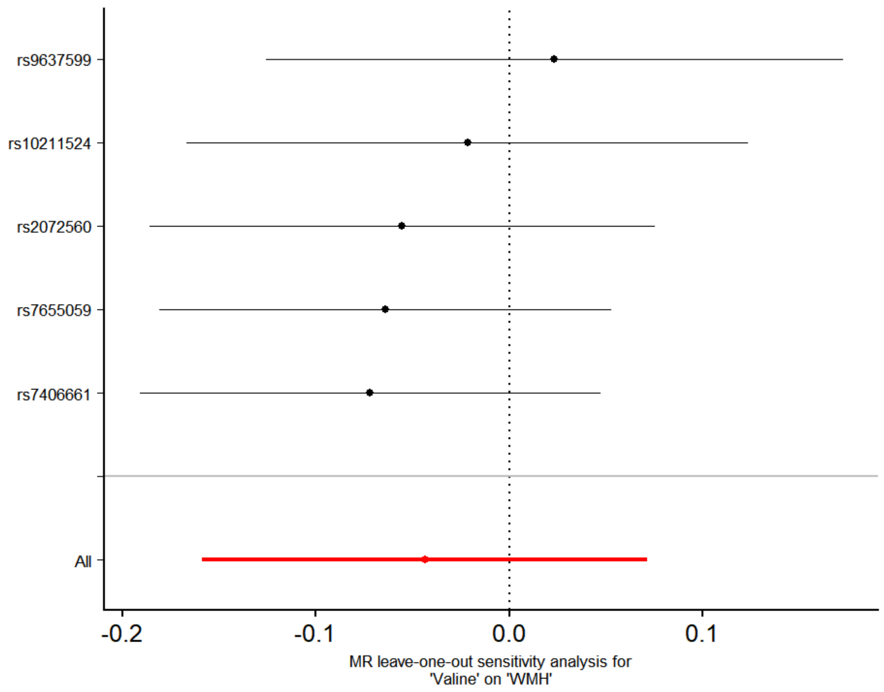

Figure S2.14 Leave-one-out plot of valine and WMH

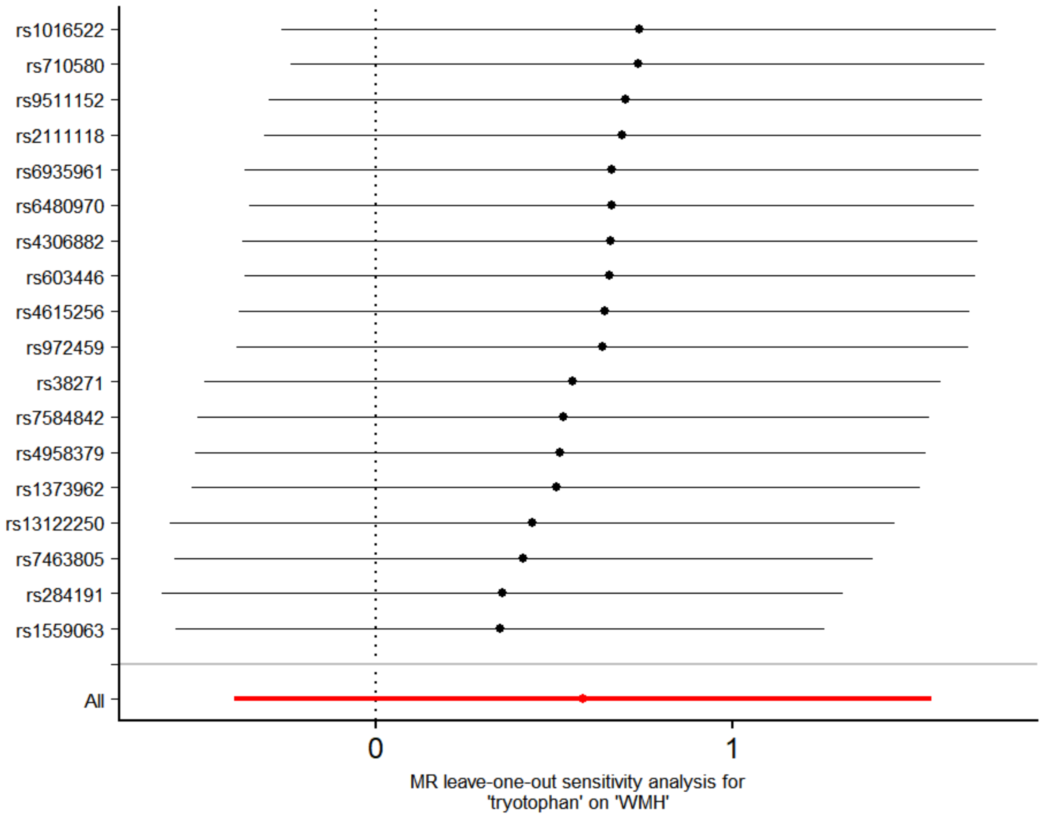

Figure S2.15 Leave-one-out plot of tryptophan and WMH

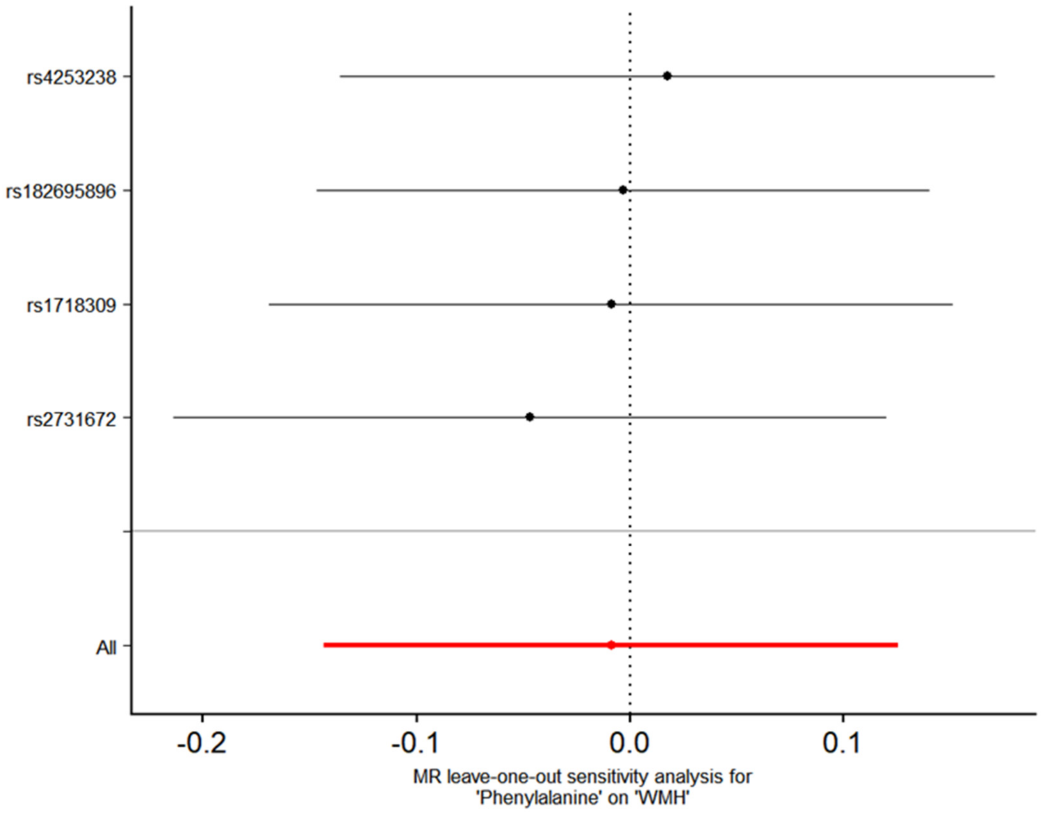

Figure S2.16 Leave-one-out plot of phenylalanine and WMH

Figure S2. Leave-one-out plots of essential nutrients exposures and WMH. The x-axis represents the beta value for the outcome obtained by removing the left SNP from the IVW analysis (i.e., the dots on each solid line). The y-axis represents the SNP removed for each analysis. Each solid line represents the 95% CI for the beta value. The bottom red line is the overall result obtained by all the SNPs of exposure.

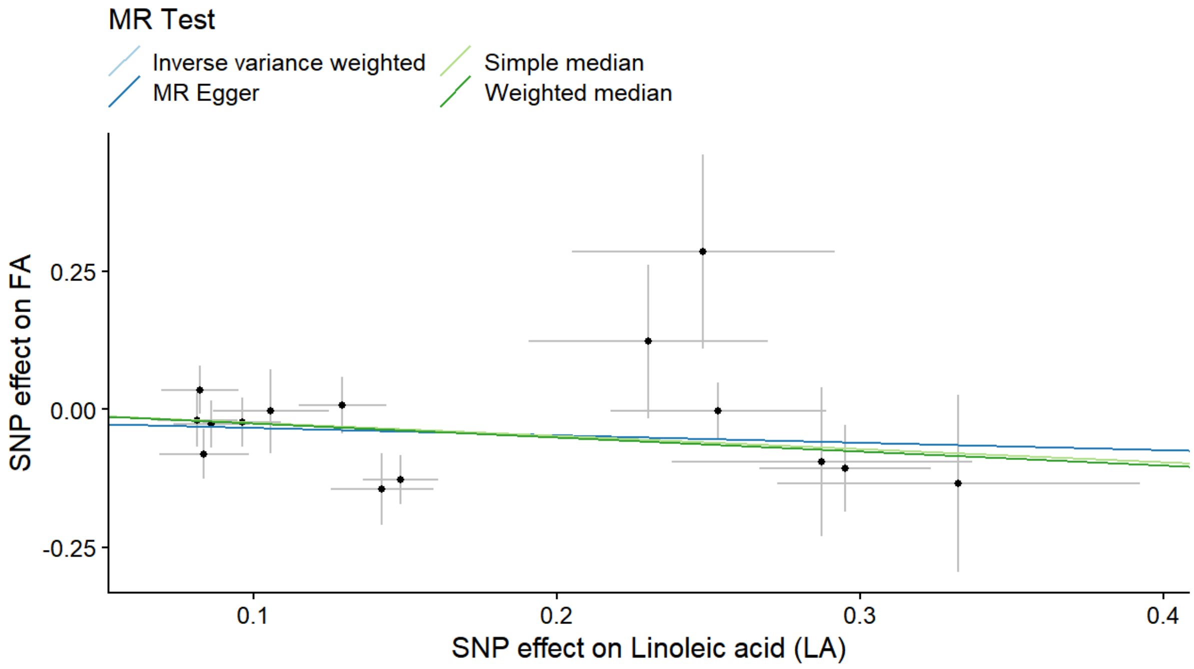

Figure S3.1 Scatterplot of linolenic acid(LA) and FA

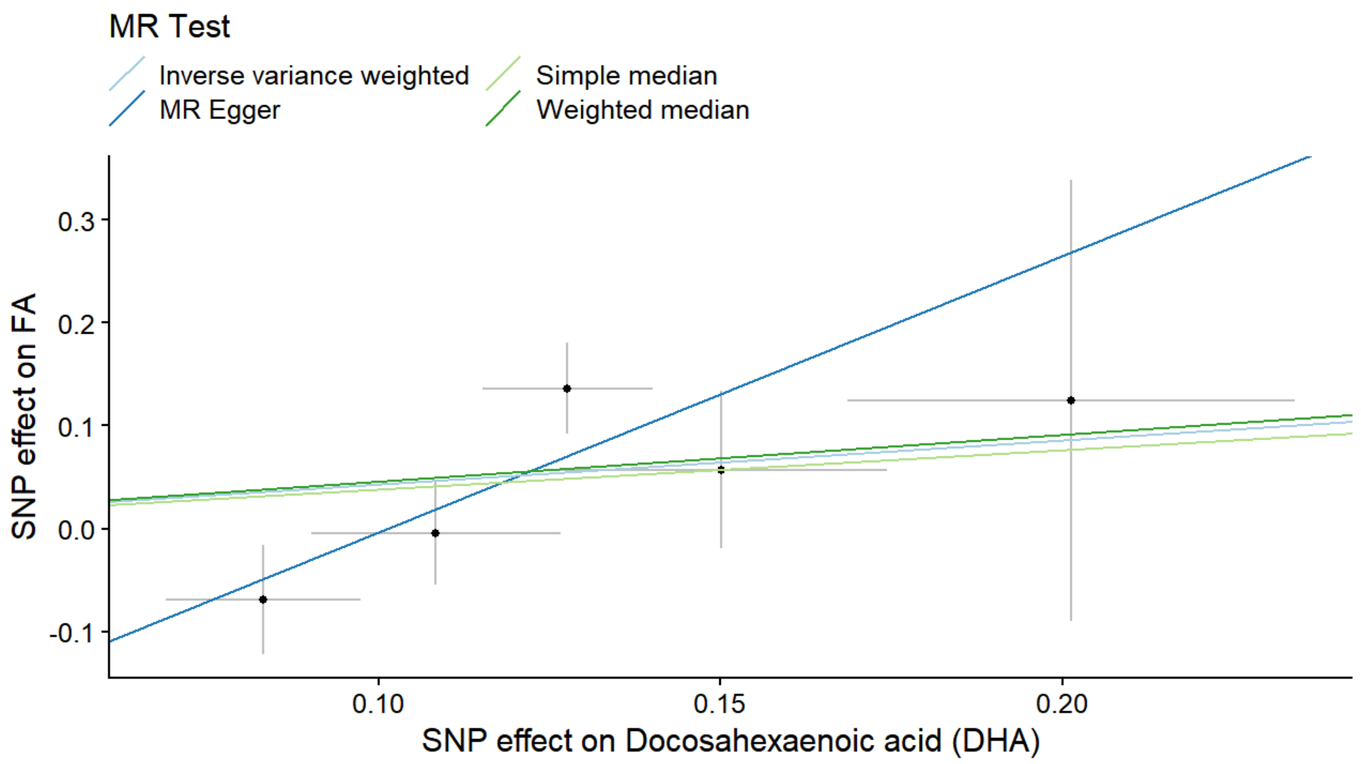

Figure S3.2 Scatterplot of docosahexaenoic acid (DHA)and FA

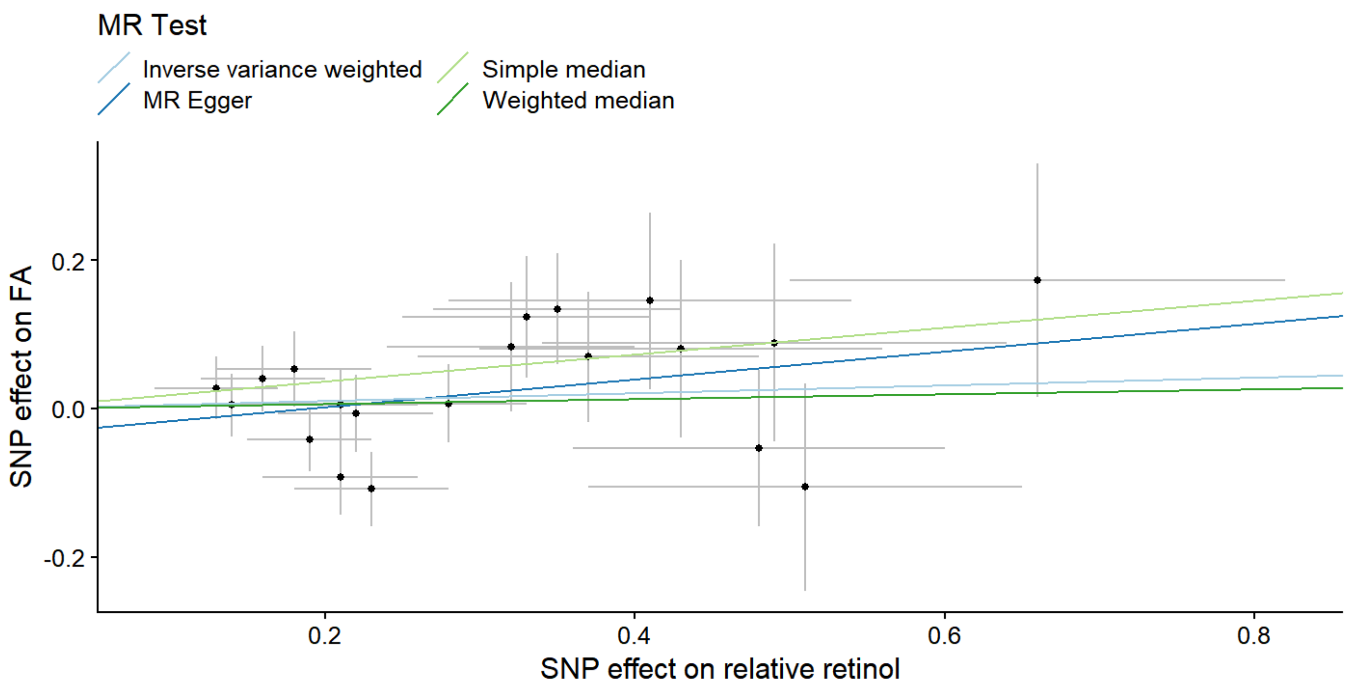

Figure S3.3 Scatterplot of relative retinol and FA

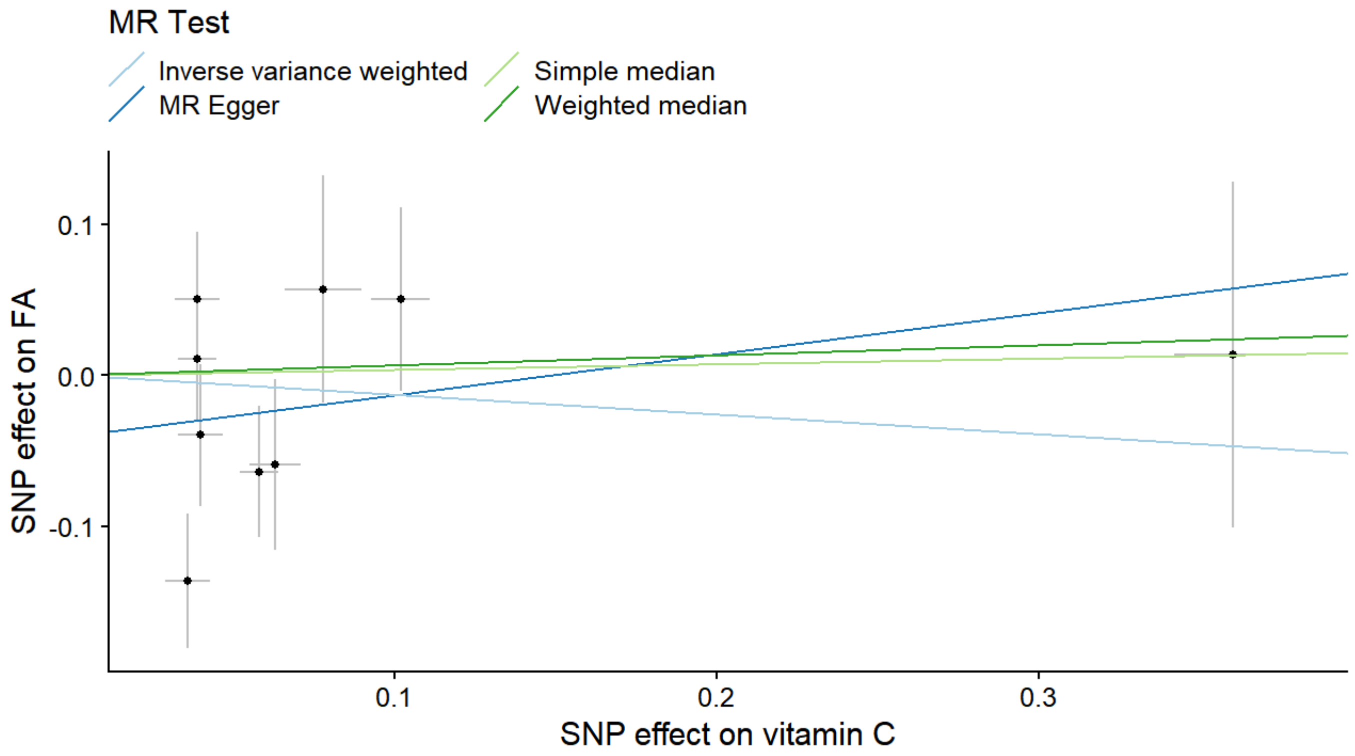

Figure S3.4 Scatterplot of vitamin C and FA

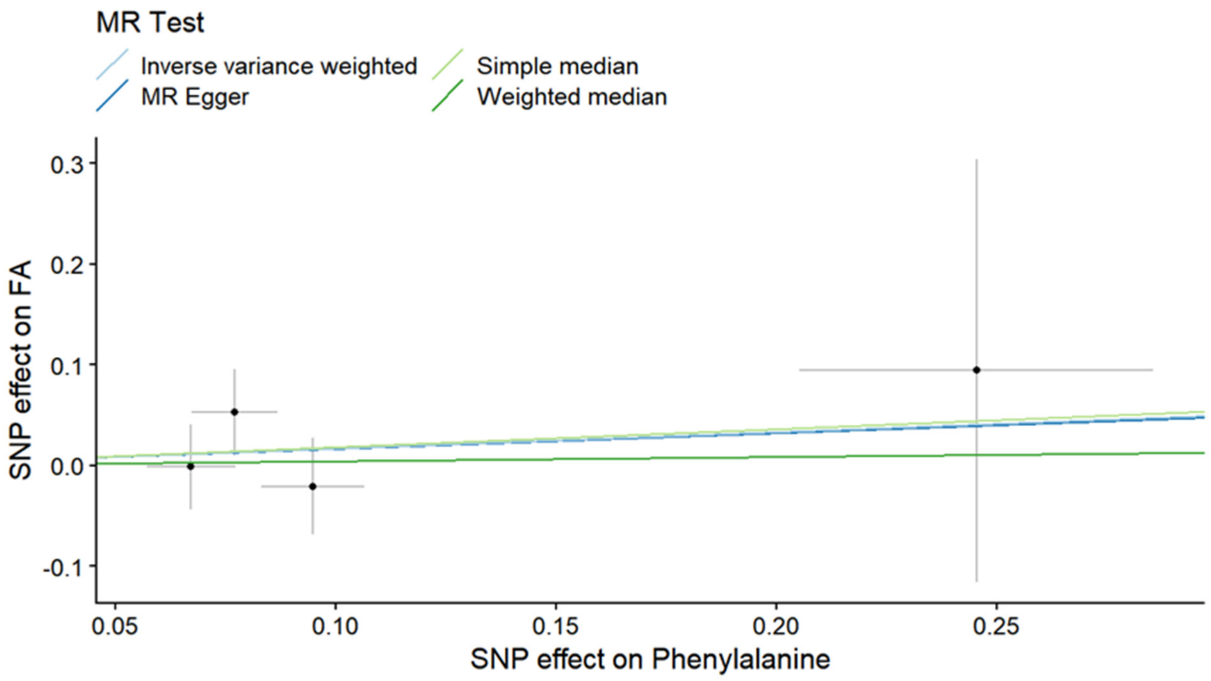

Figure S3.5 Scatterplot of absolute phenylalanine and FA

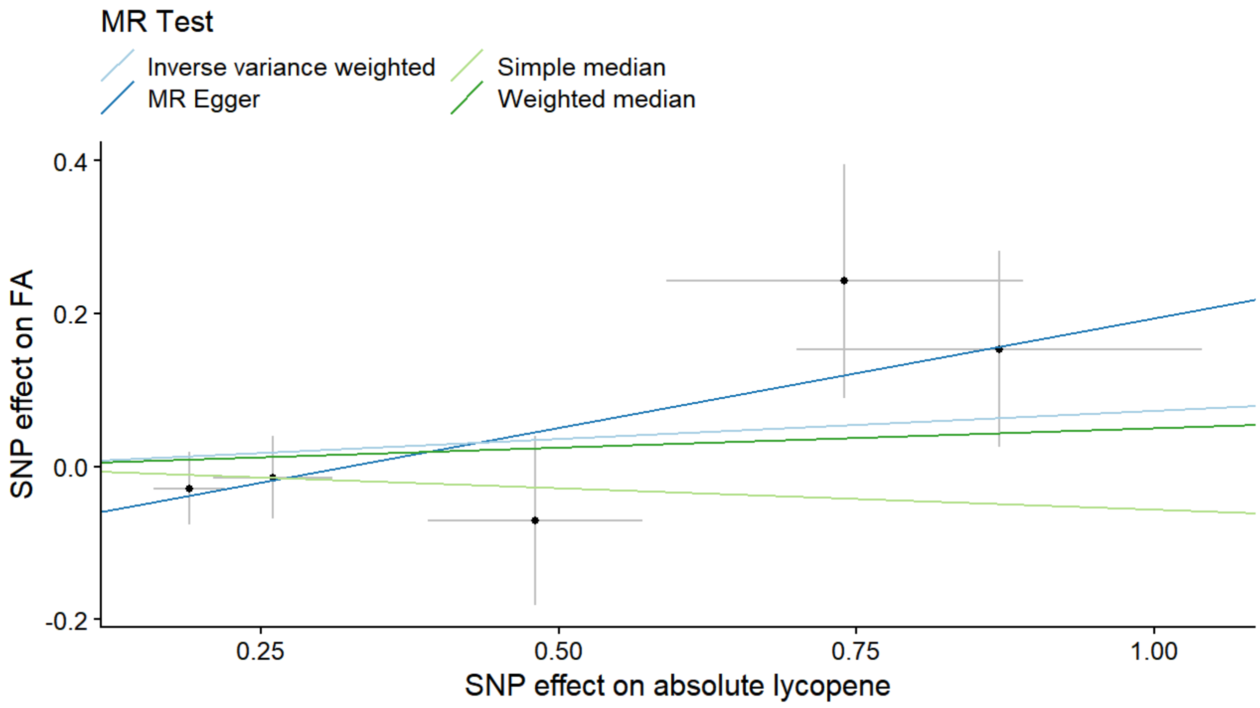

Figure S3.6 Scatterplot of absolute lycopene and FA

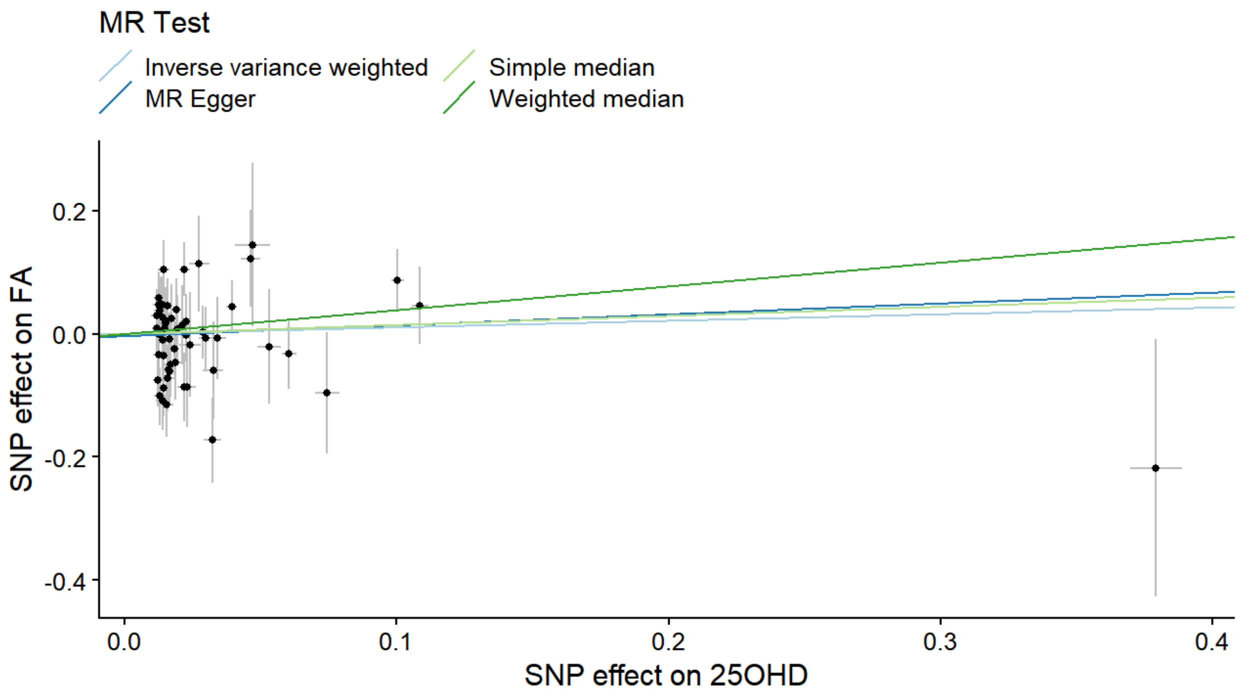

Figure S3.7 Scatterplot of 25OHD and FA

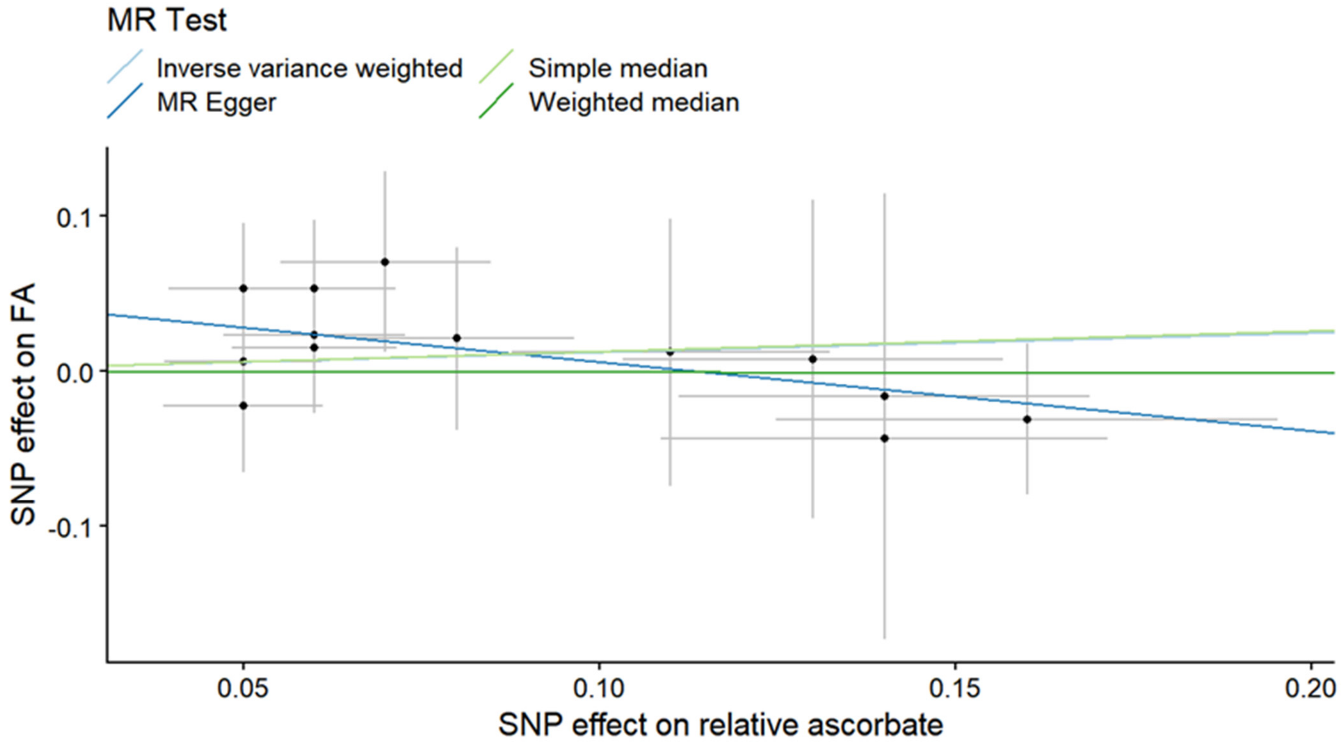

Figure S3.8 Scatterplot of relative ascorbate and FA

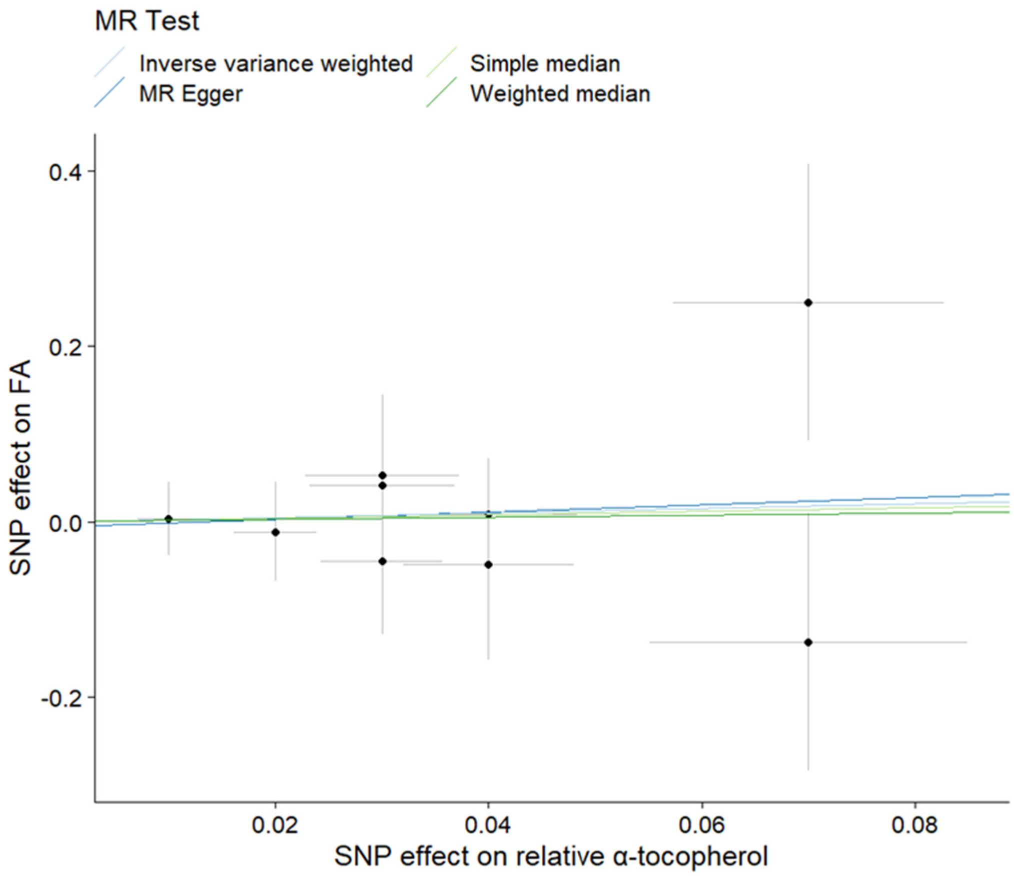

Figure S3.9 Scatterplot of relative α -tocopherol and FA

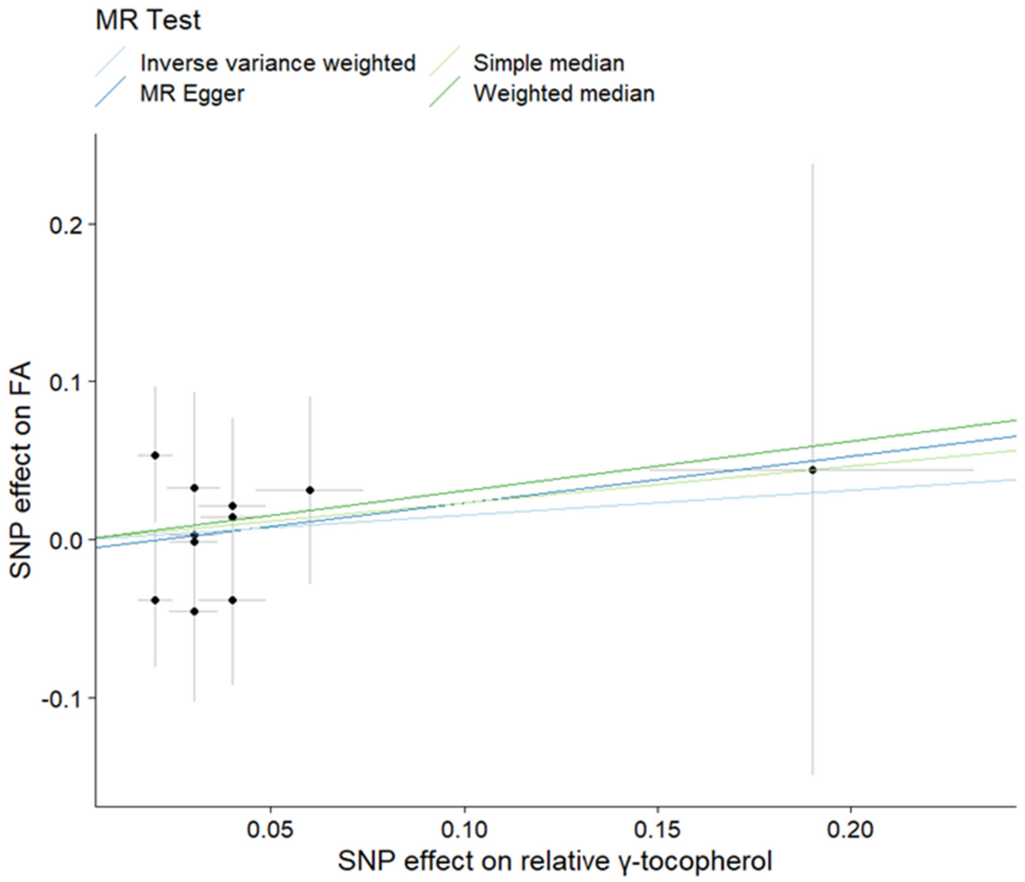

Figure S3.10 Scatterplot of relative γ -tocopherol and FA

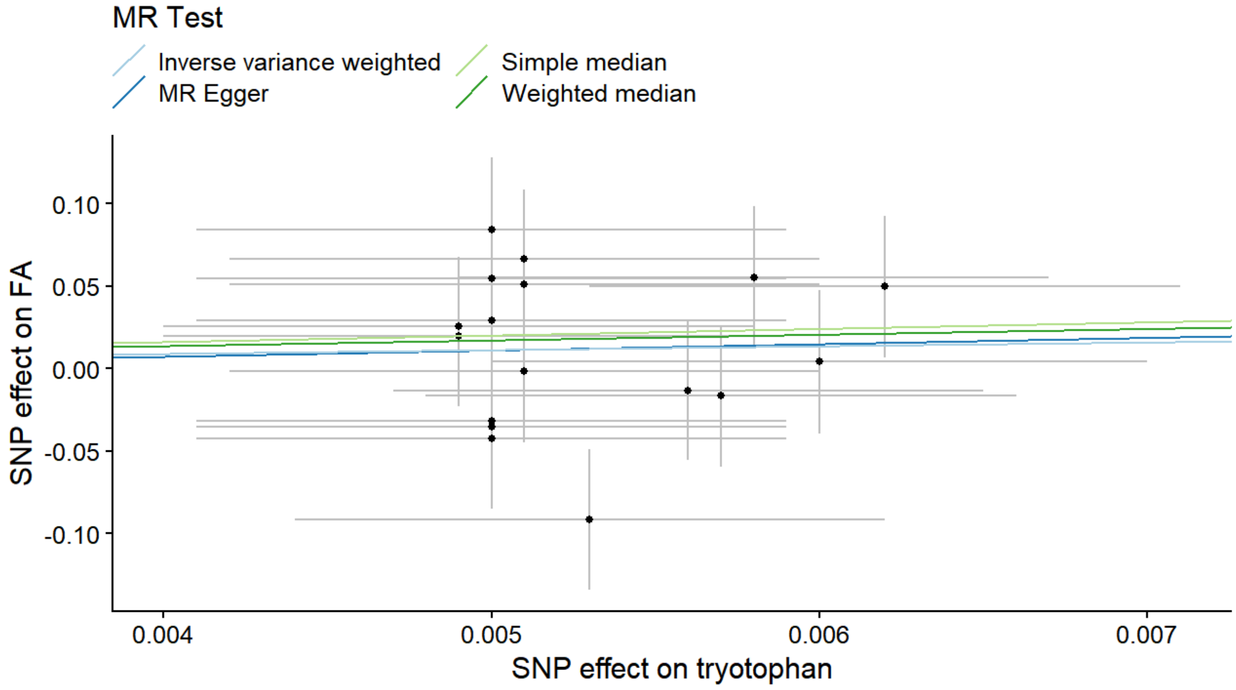

Figure S3.11 Scatterplot of tryptophan and FA

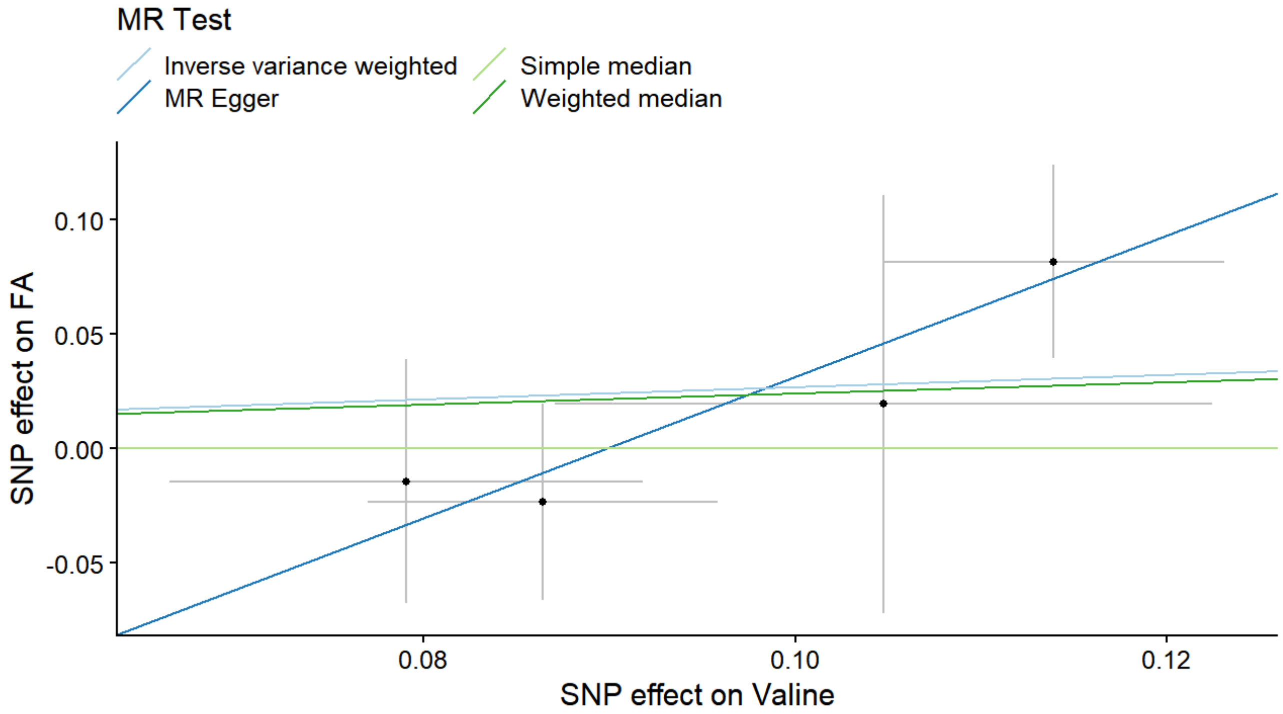

Figure S3.12 Scatterplot of valine and FA

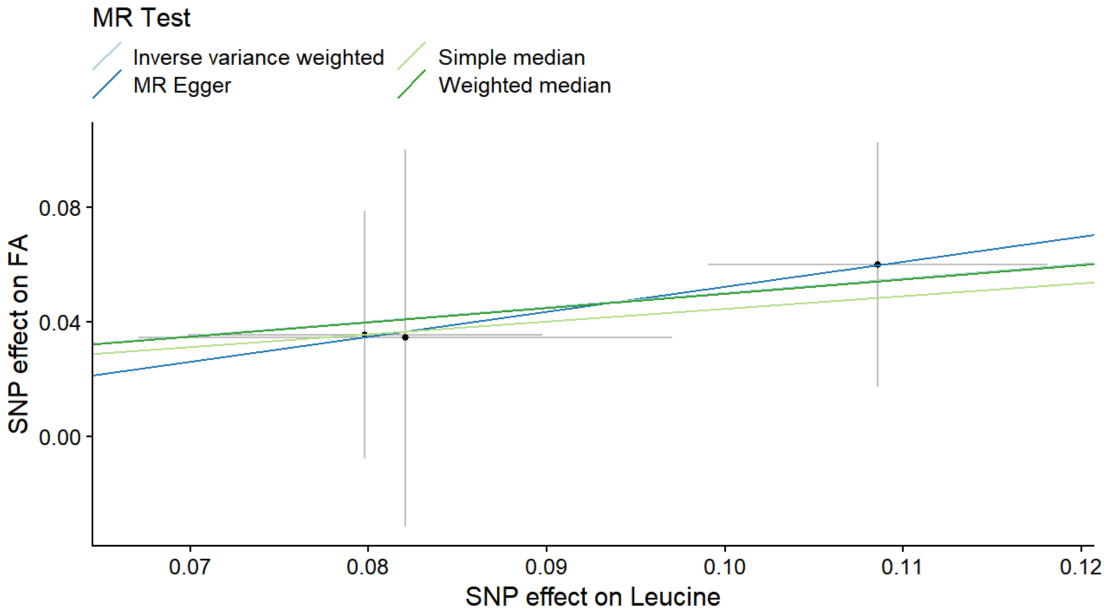

Figure S3.13 Scatterplot of leucine and FA

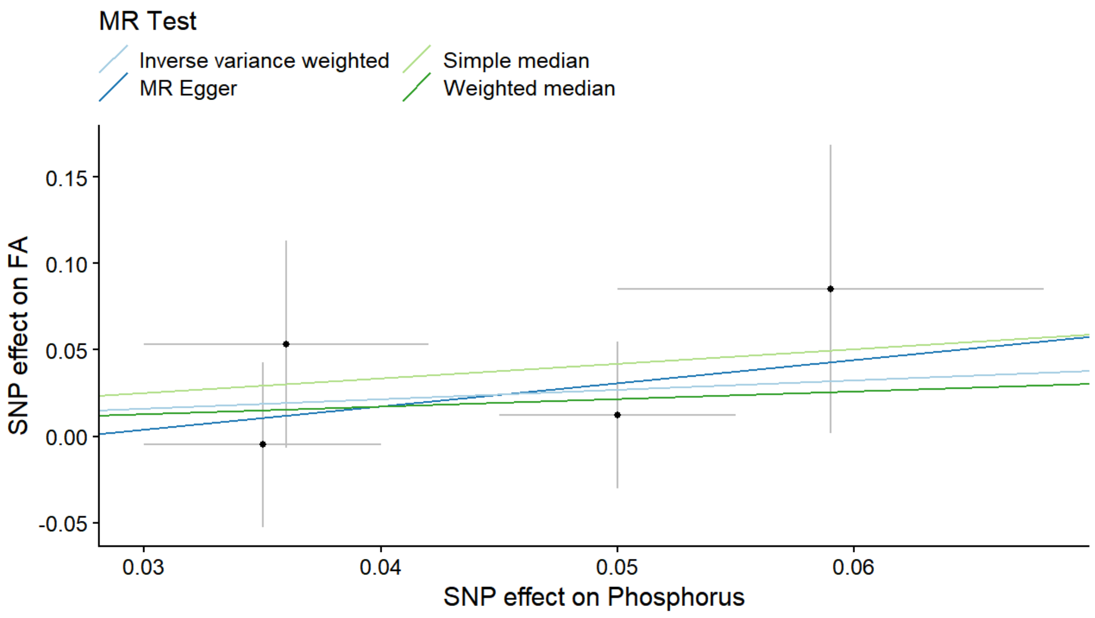

Figure S3.14 Scatterplot of phosphorus and FA

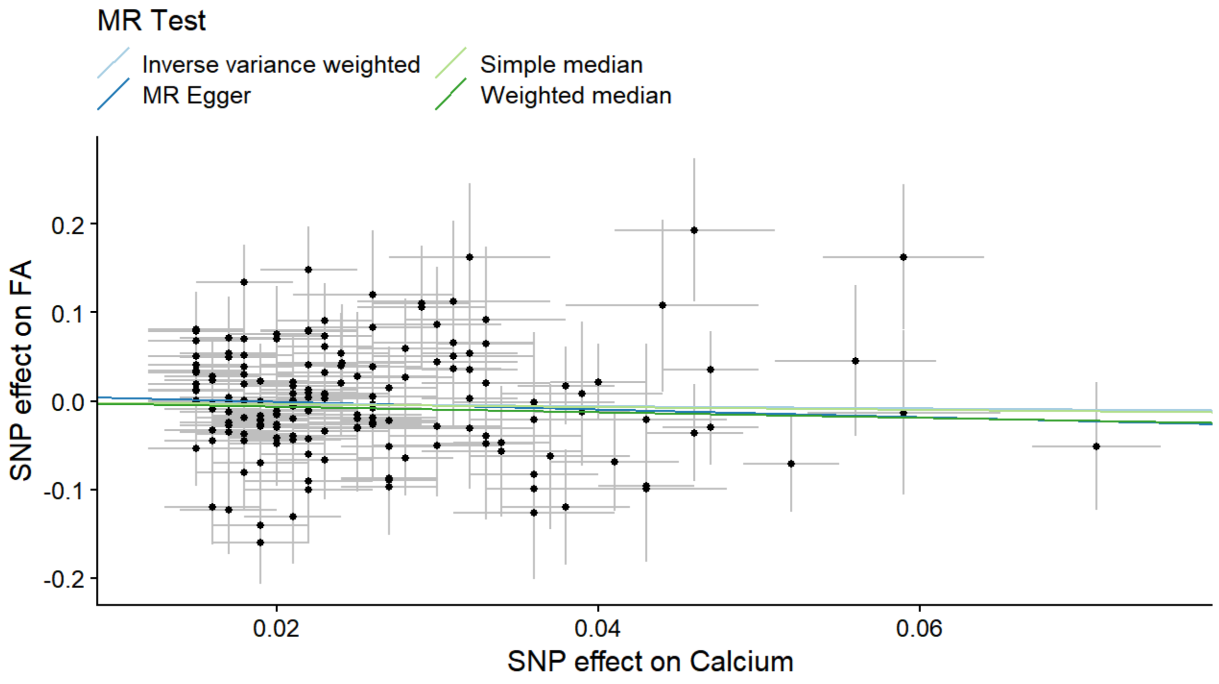

Figure S3.15 Scatterplot of calcium and FA

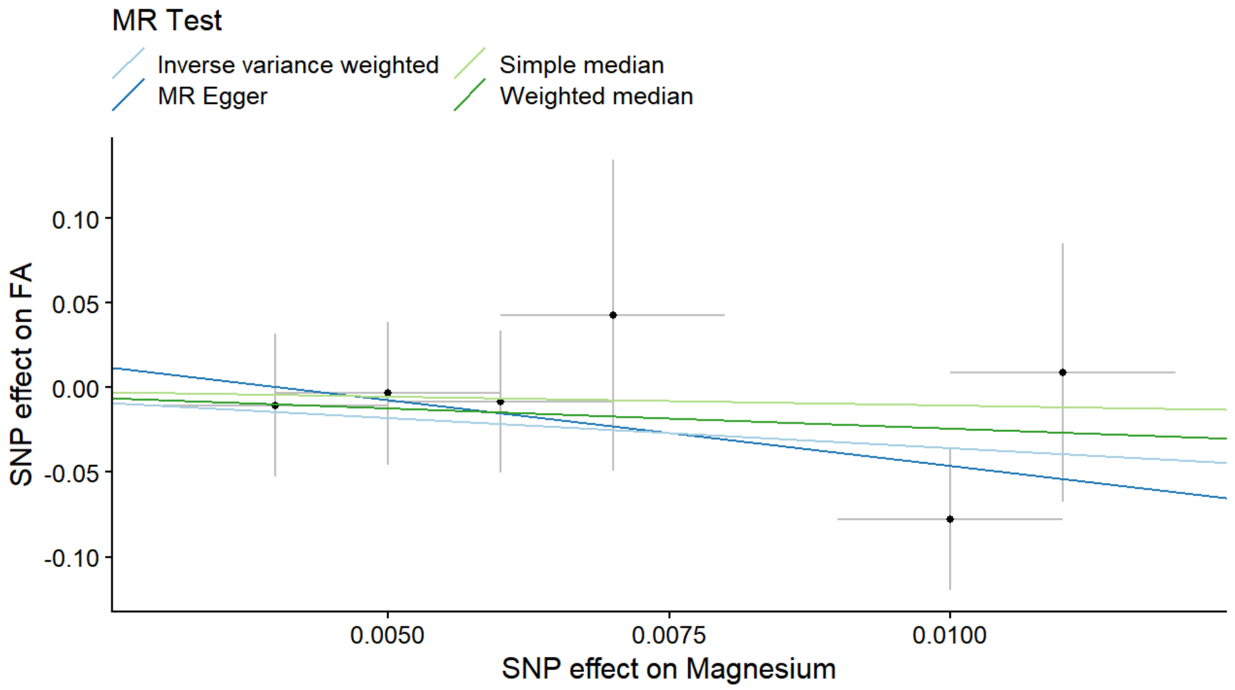

Figure S3.16 Scatterplot of magnesium and FA

Figure S3. Scatterplots of essential nutrients exposures and FA. The x-axis represents the previously published β-estimate for the association between each SNP and essential nutrients. The y-axis represents the β-estimate for the association between each SNP and risk of cerebral hemorrhage. The slope of each line corresponds to the estimated MR effect per method.

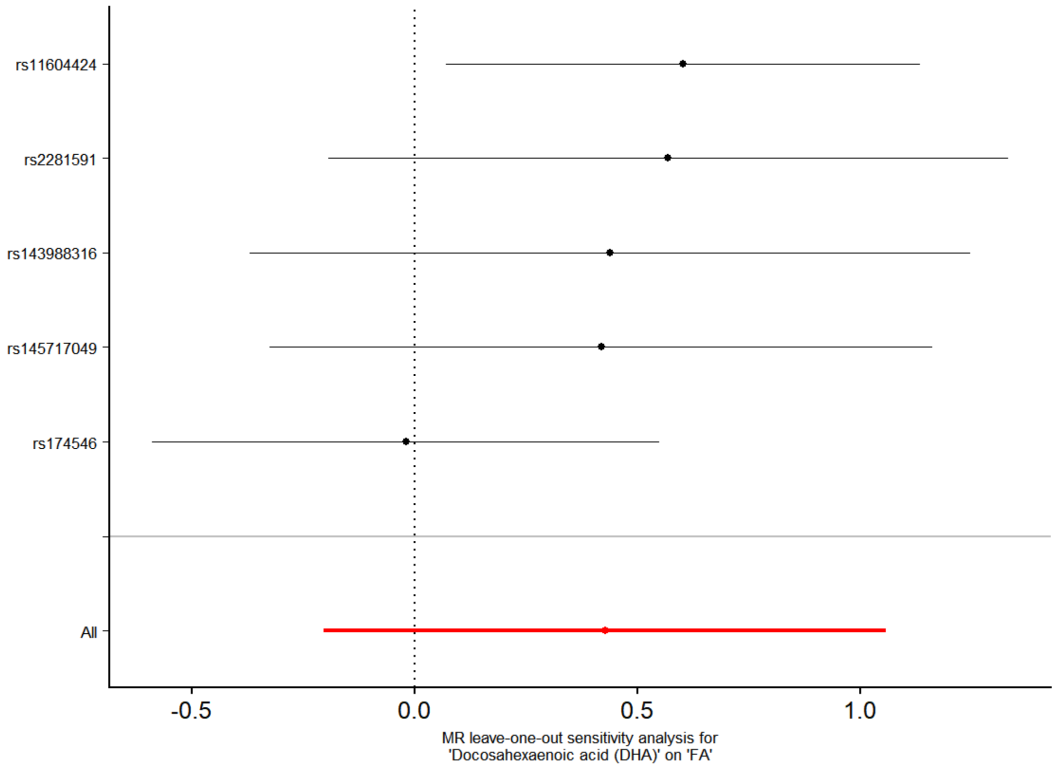

Figure S4.1 Leave-one-out plot of docosahexaenoic acid (DHA) and FA

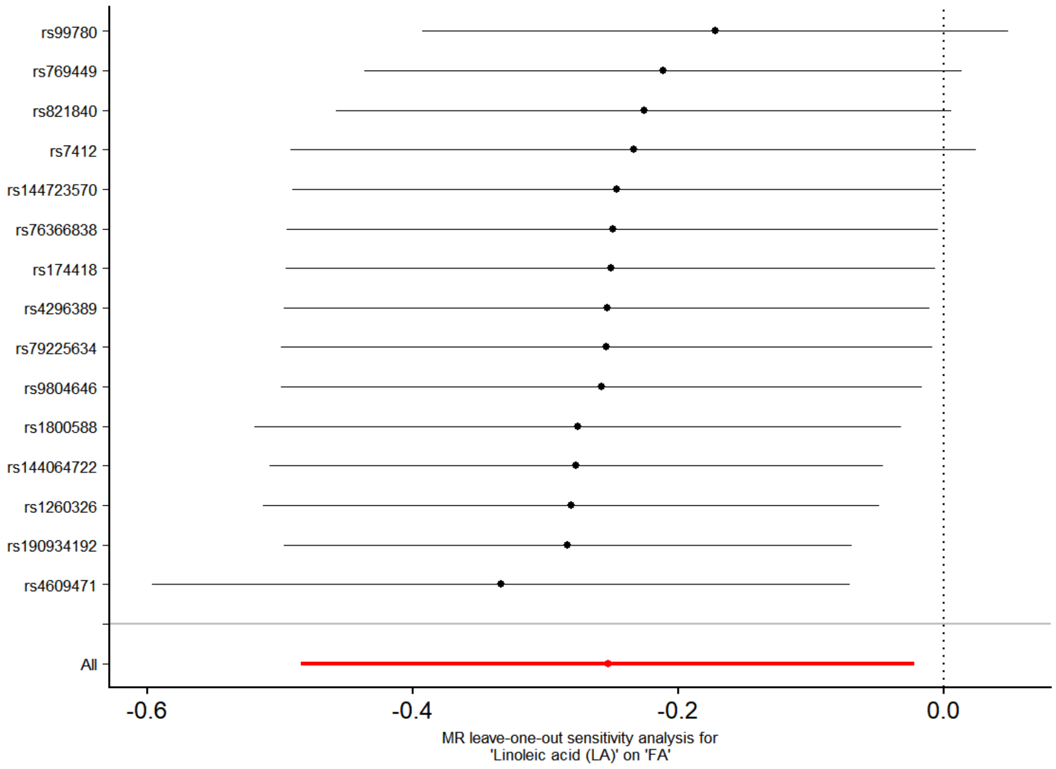

Figure S4.2 Leave-one-out plot of linolenic acid(LA) and FA

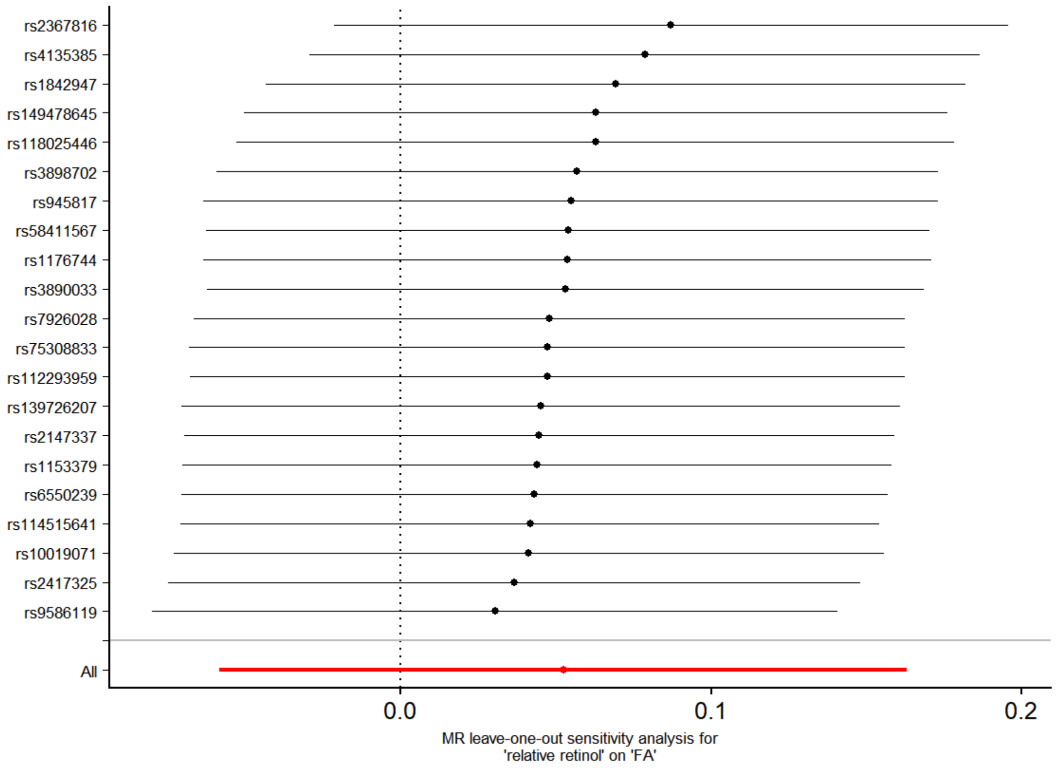

Figure S4.3 Leave-one-out plot of relative retinol and FA

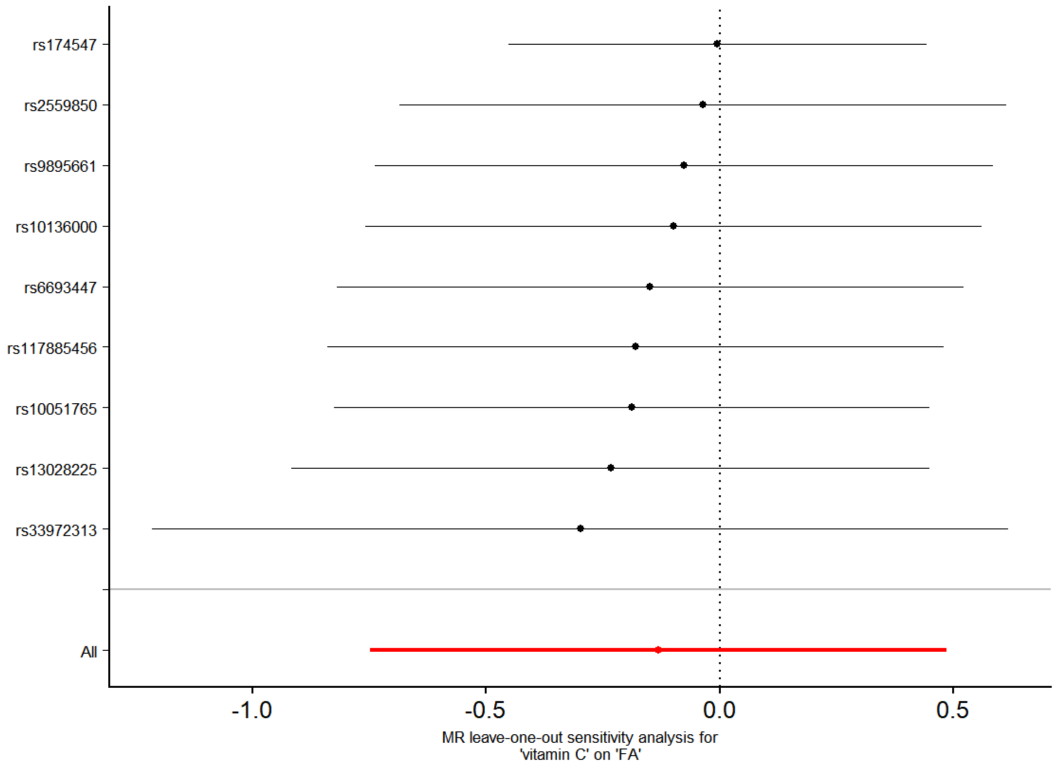

Figure S4.4 Leave-one-out plot of vitamin C and FA

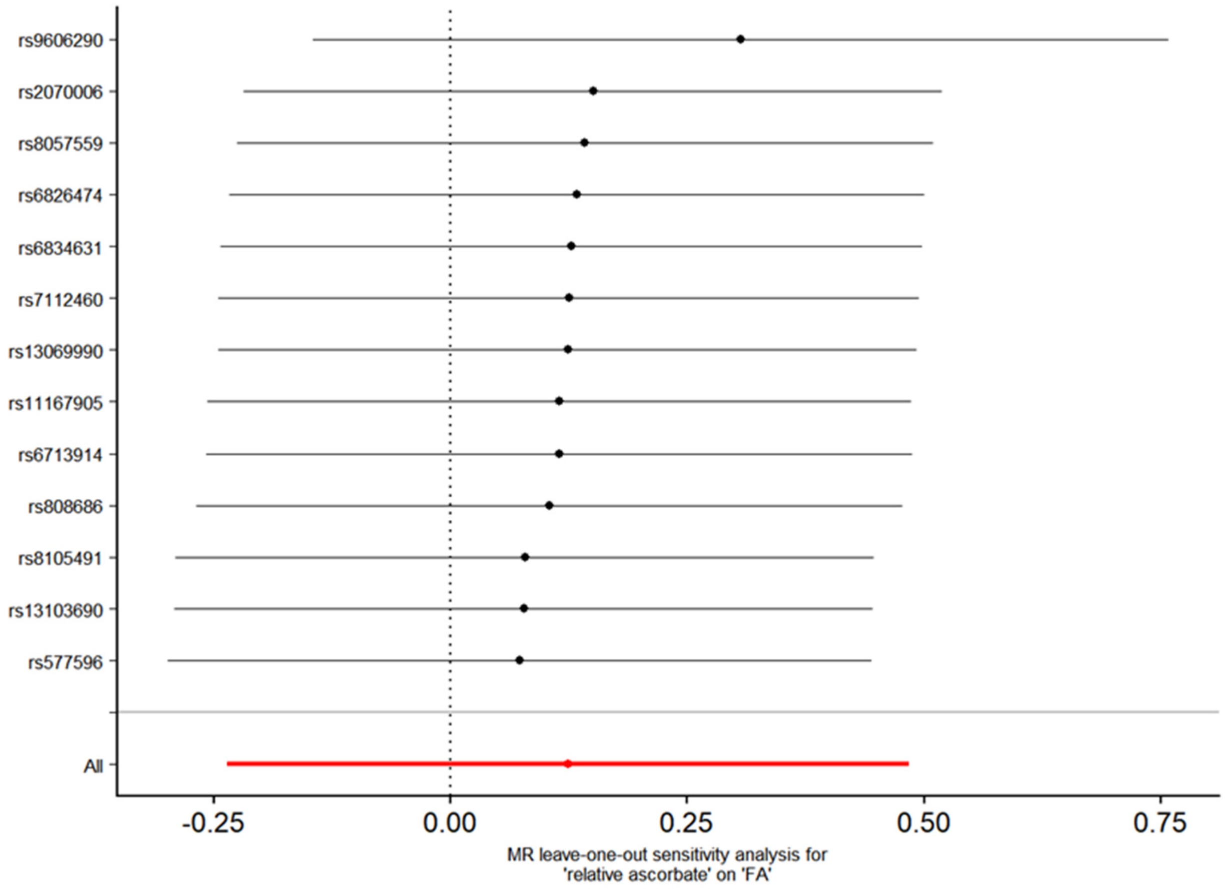

Figure S4.5 Leave-one-out plot of relative ascorbate and FA

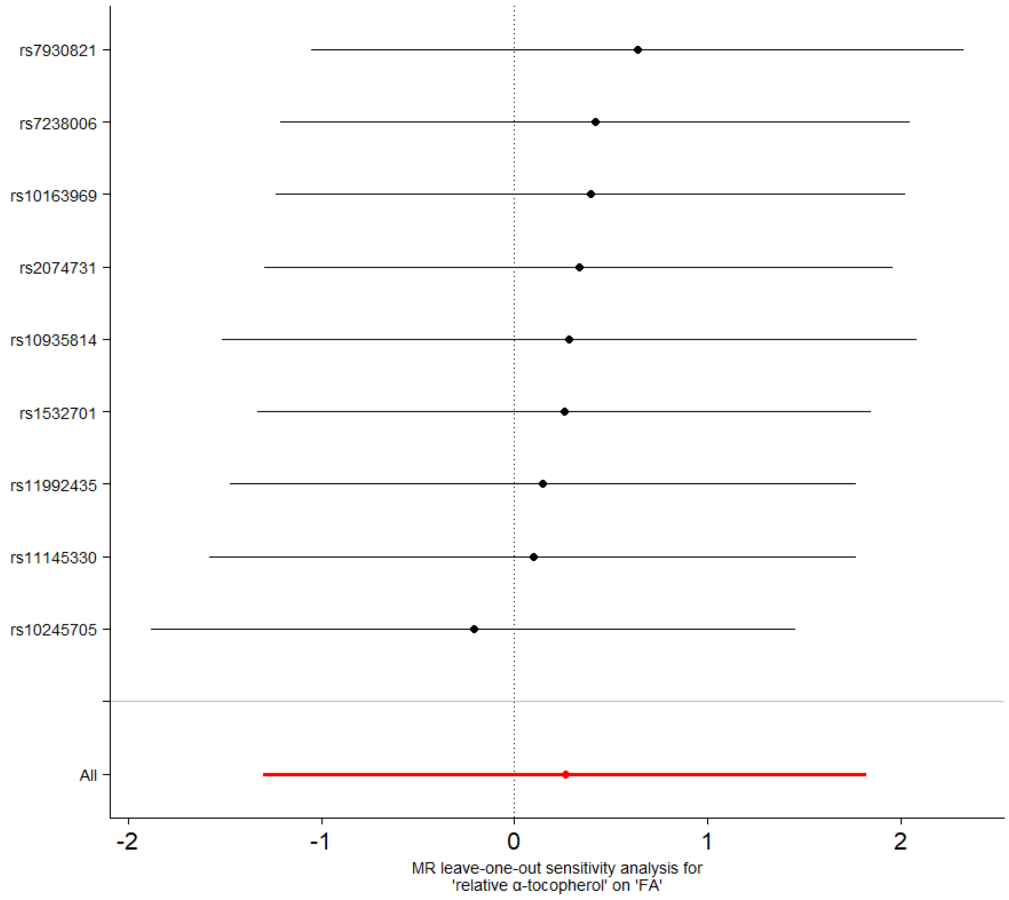

Figure S4.6 Leave-one-out plot of relative α -tocopherol and FA

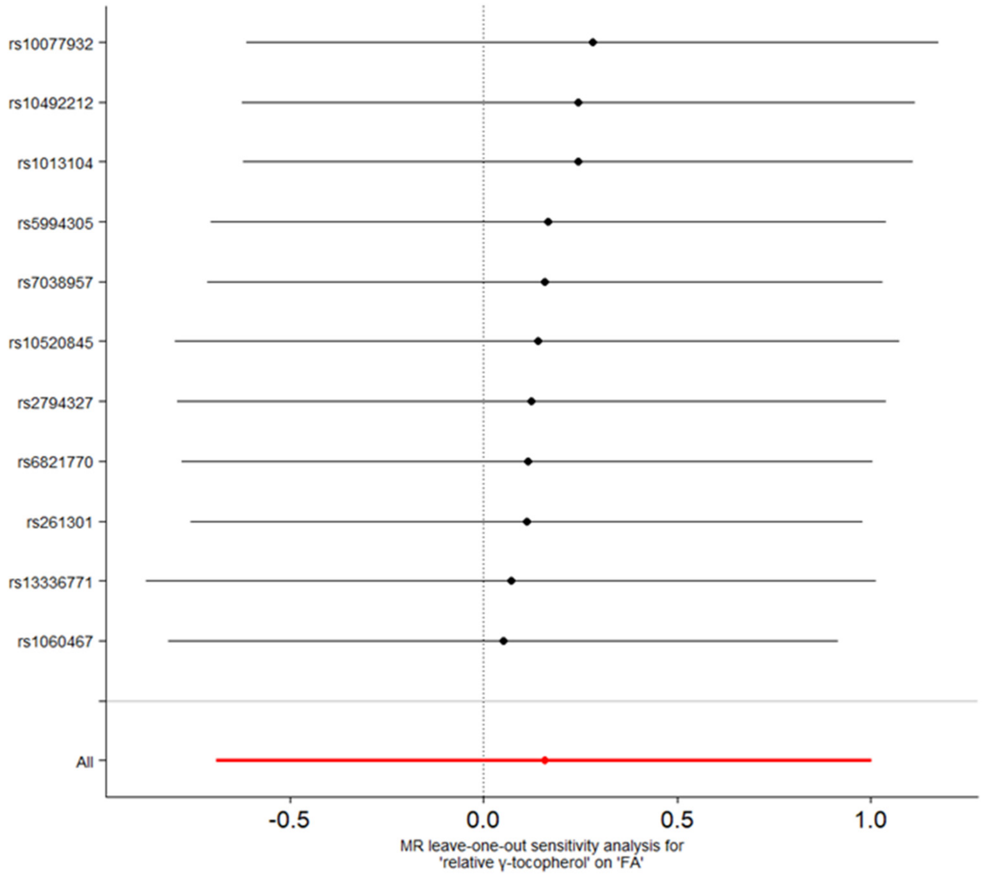

Figure S4.7 Leave-one-out plot of relative γ -tocopherol and FA

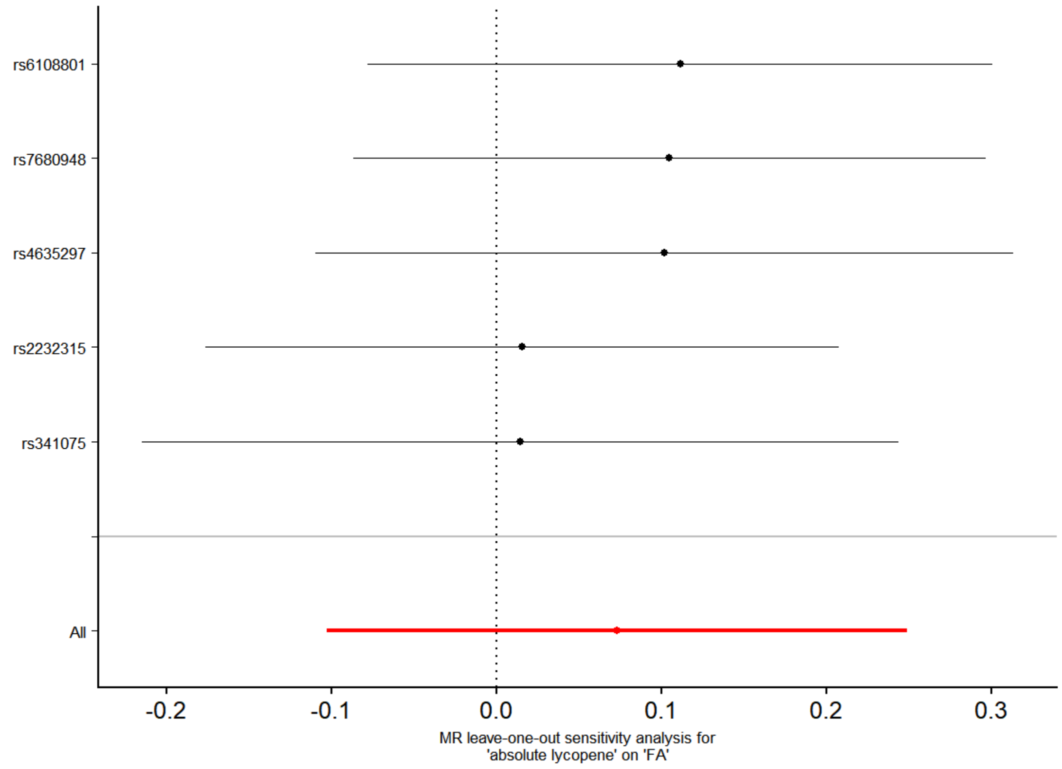

Figure S4.8 Leave-one-out plot of absolute lycopene and FA

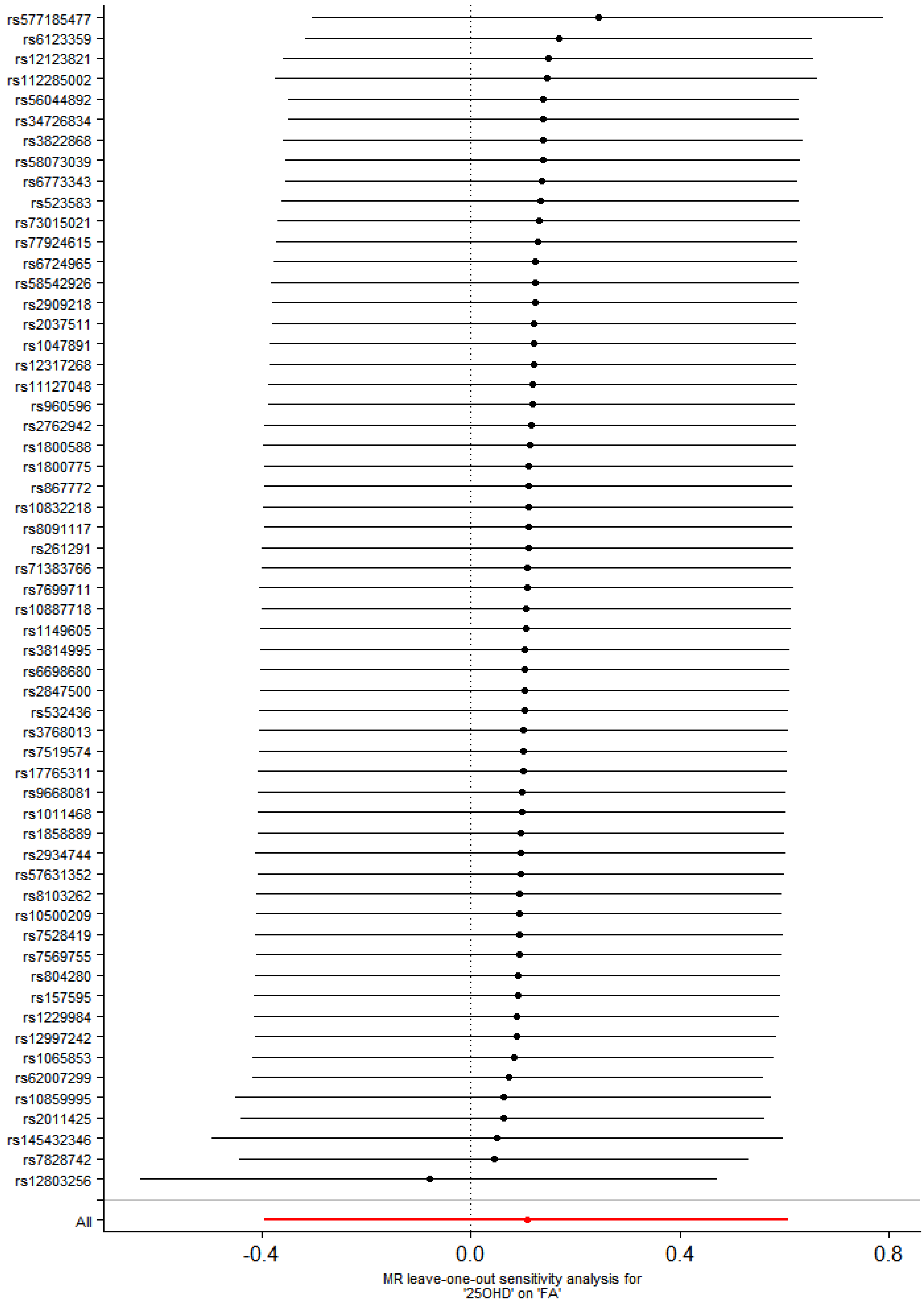

Figure S4.9 Leave-one-out plot of 25OHD and FA

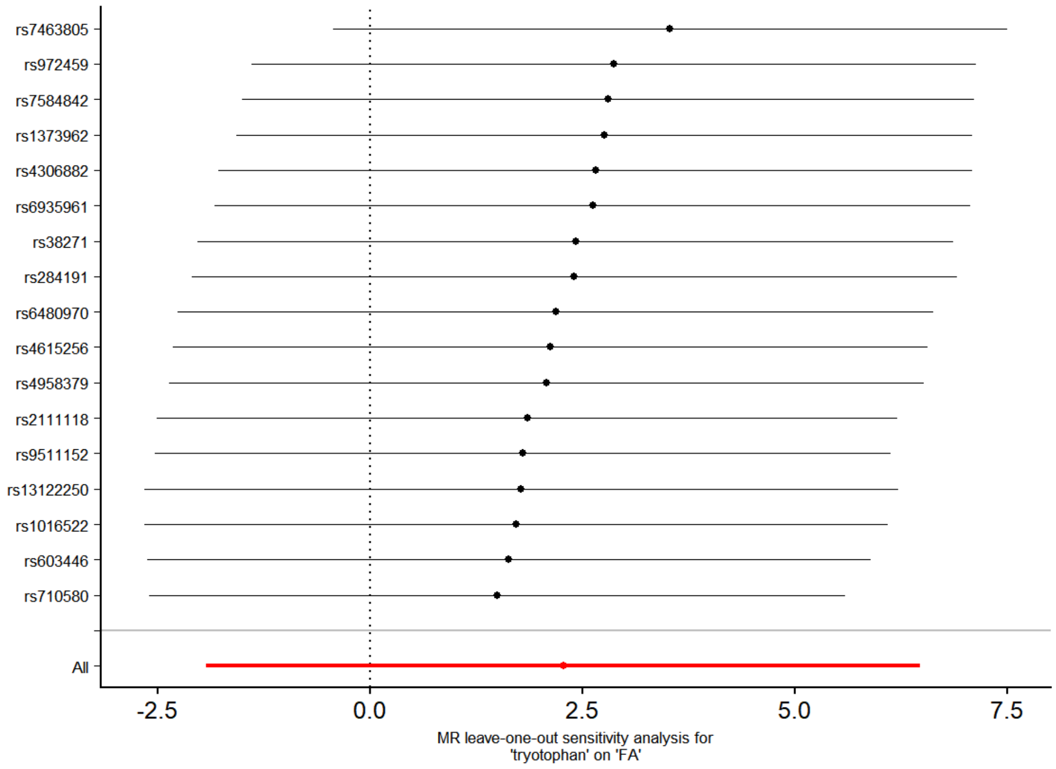

Figure S4.10 Leave-one-out plot of tryptophan and FA

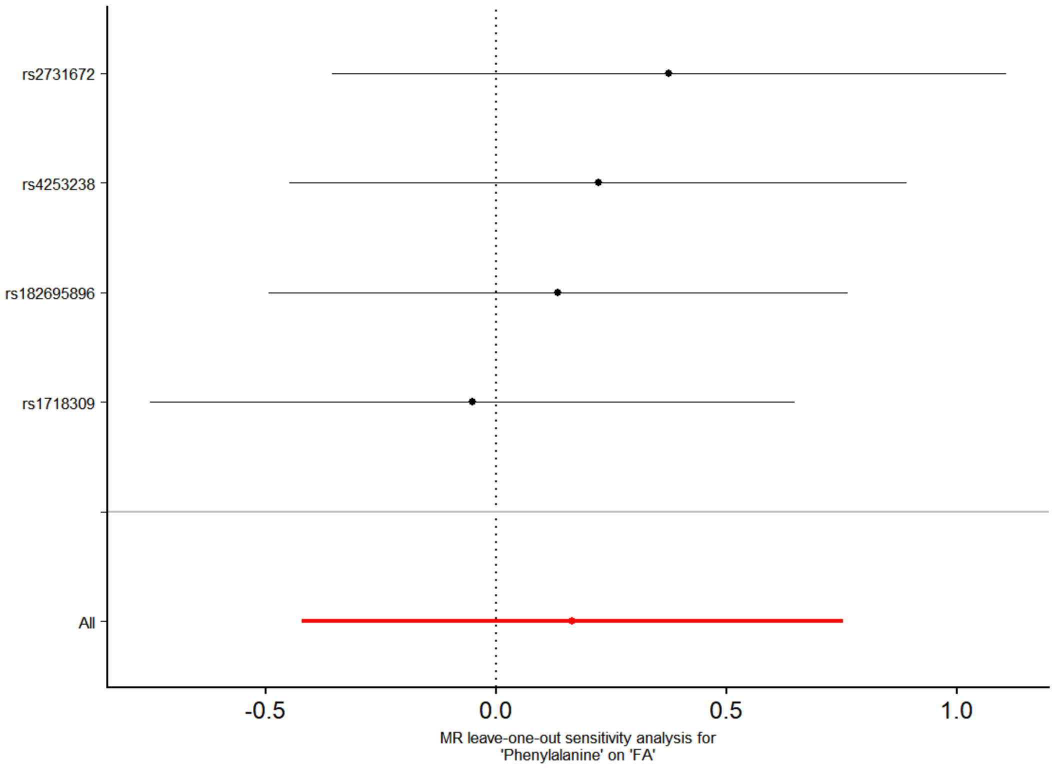

Figure S4.11 Leave-one-out plot of phenylalanine and FA

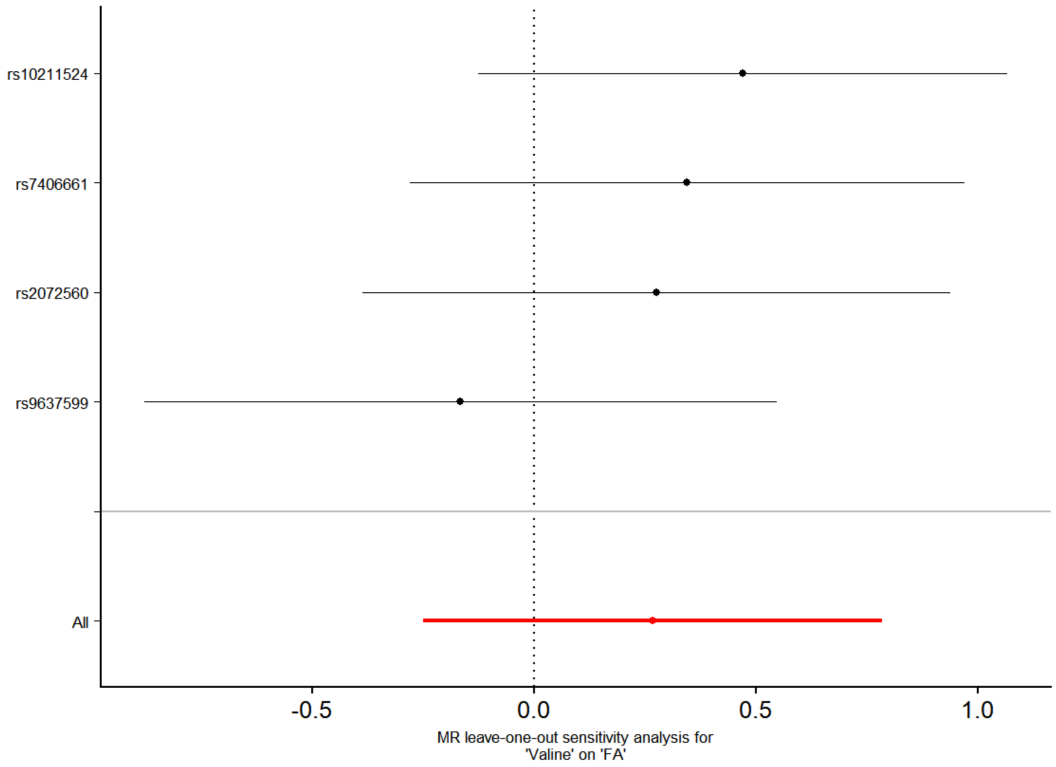

Figure S4.12 Leave-one-out plot of valine and FA

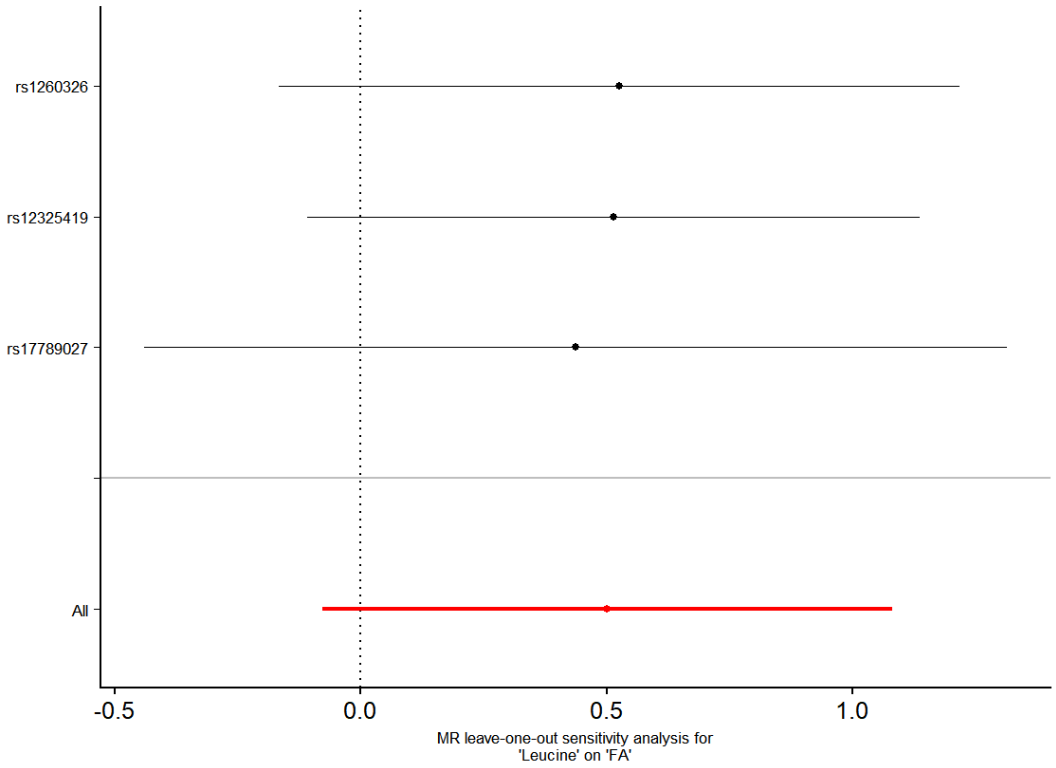

Figure S4.13 Leave-one-out plot of leucine and FA

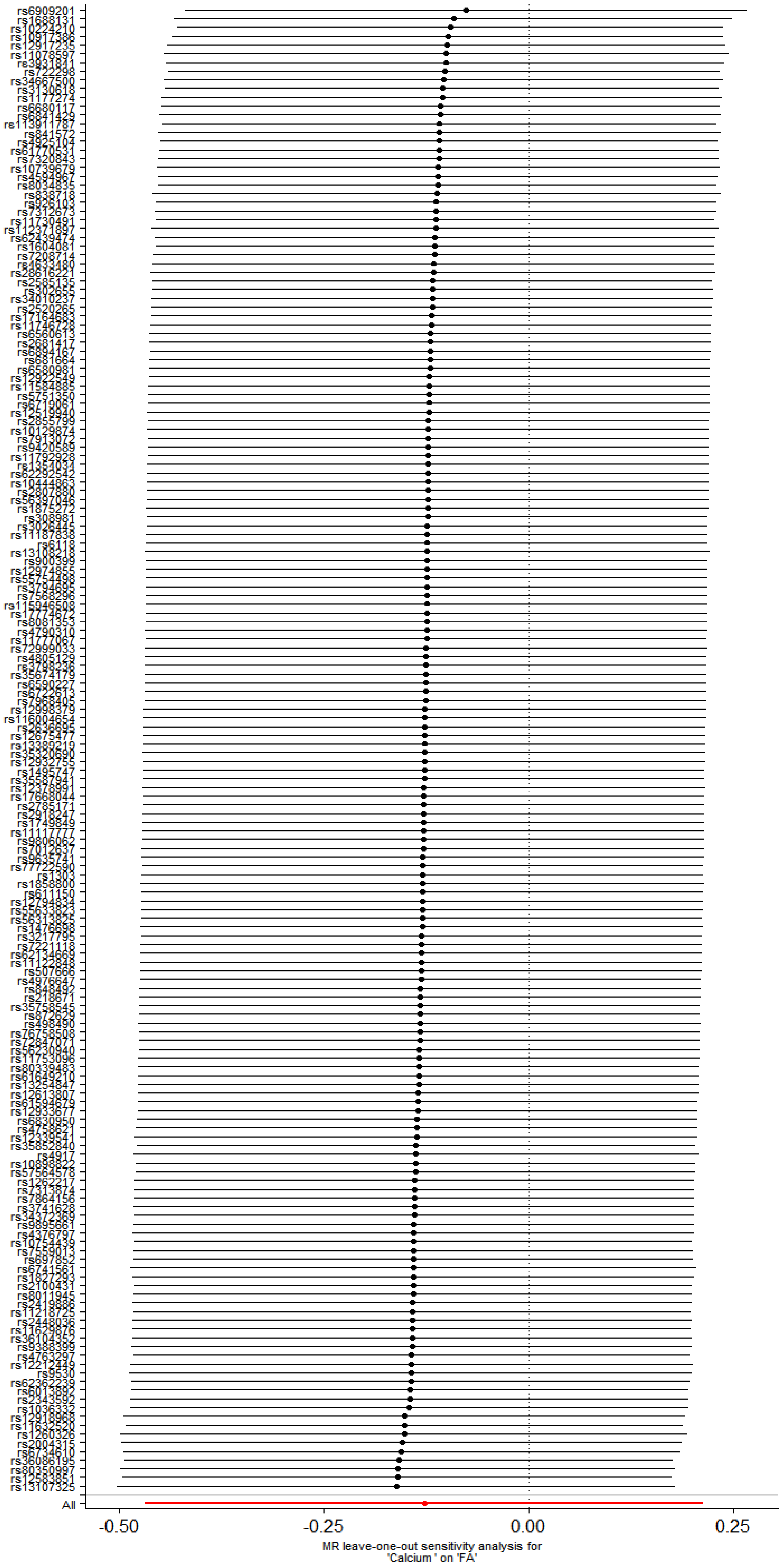

Figure S4.14 Leave-one-out plot of calcium and FA

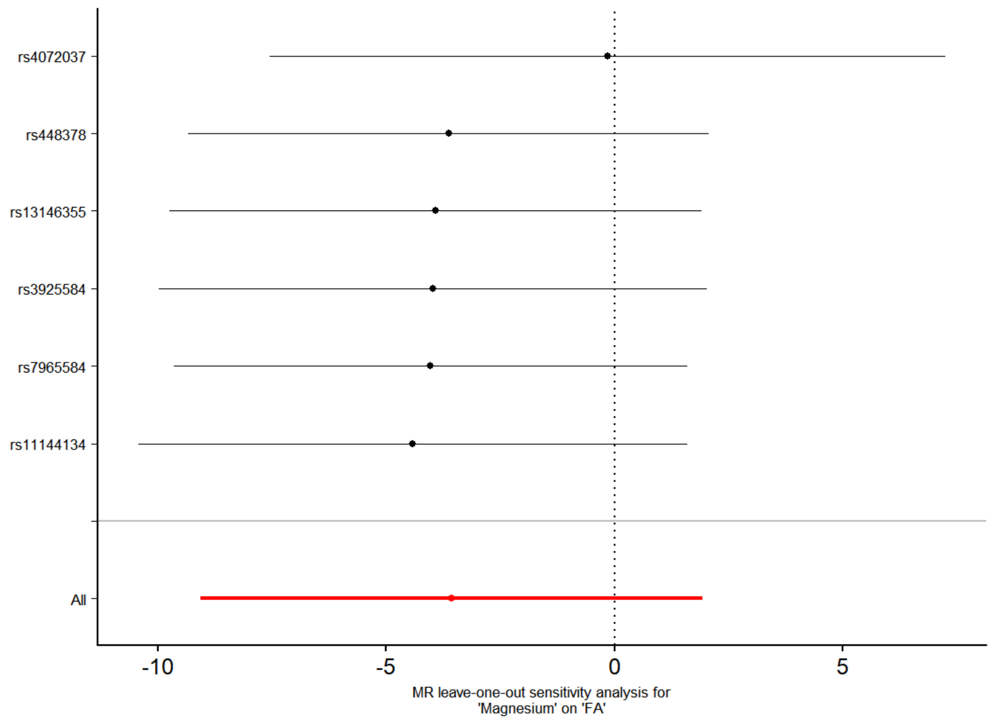

Figure S4.15 Leave-one-out plot of magnesium and FA

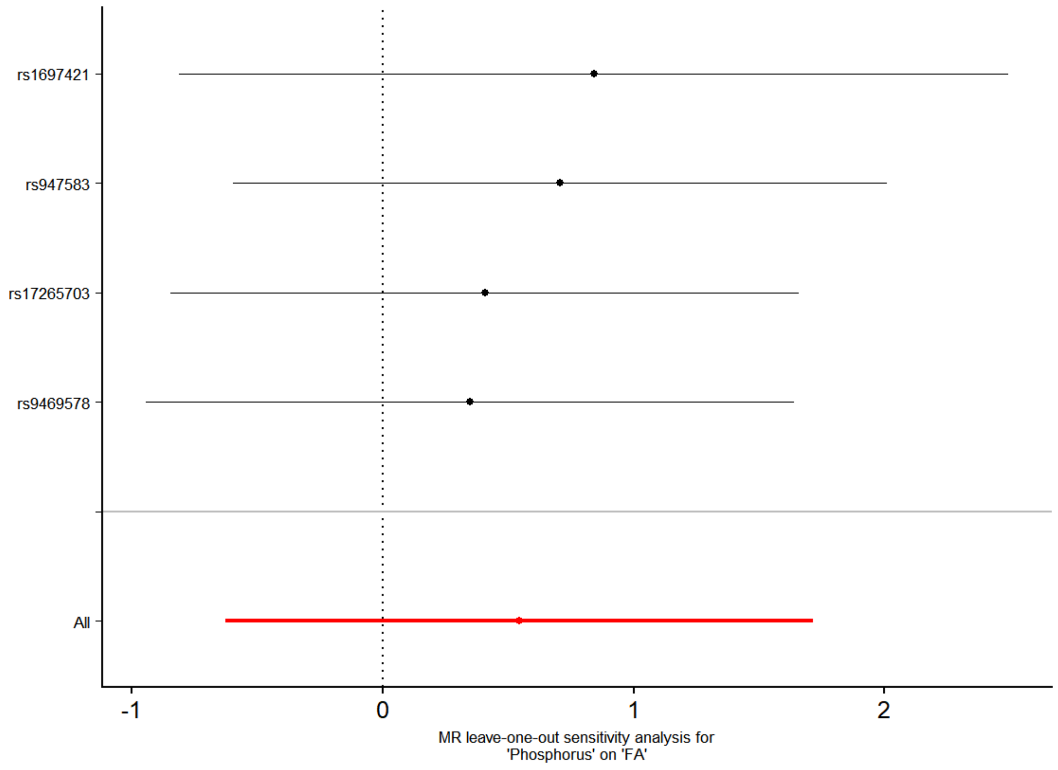

Figure S4.16 Leave-one-out plot of Phosphorus and FA

Figure S4 Leave-one-out plots of essential nutrients exposures and FA. The x-axis represents the beta value for the outcome obtained by removing the left SNP from the IVW analysis (i.e., the dots on each solid line). The y-axis represents the SNP removed for each analysis. Each solid line represents the 95% CI for the beta value. The bottom red line is the overall result obtained by all the SNPs of exposure.

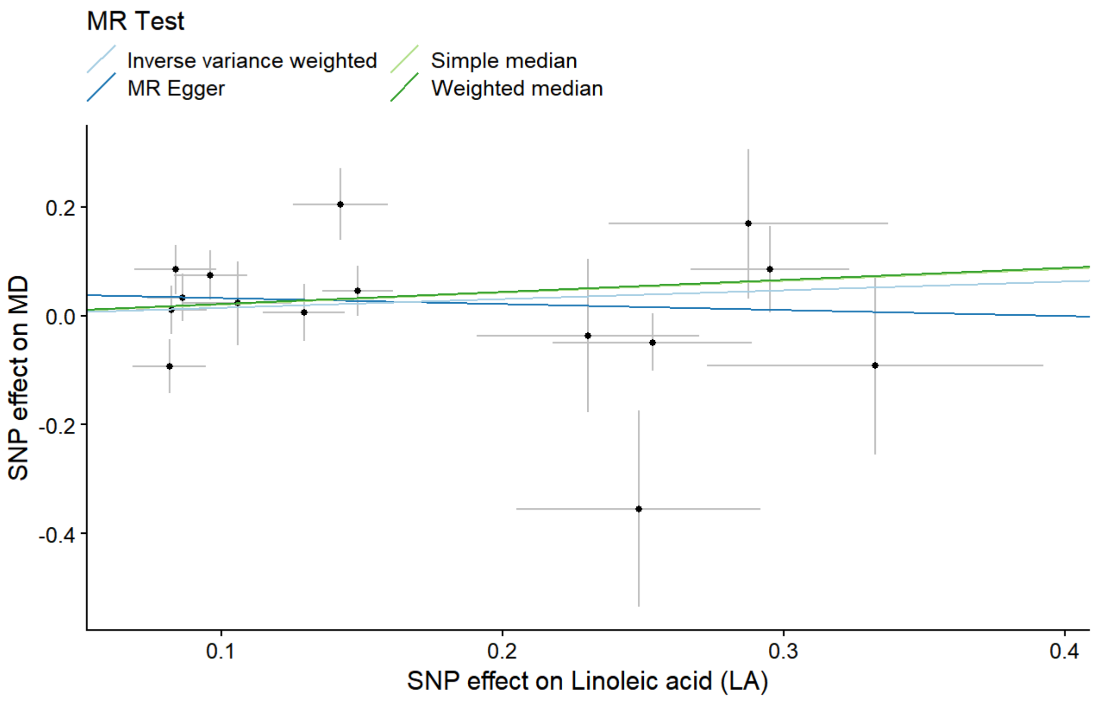

Figure S5.1 Scatterplot of linolenic acid and MD

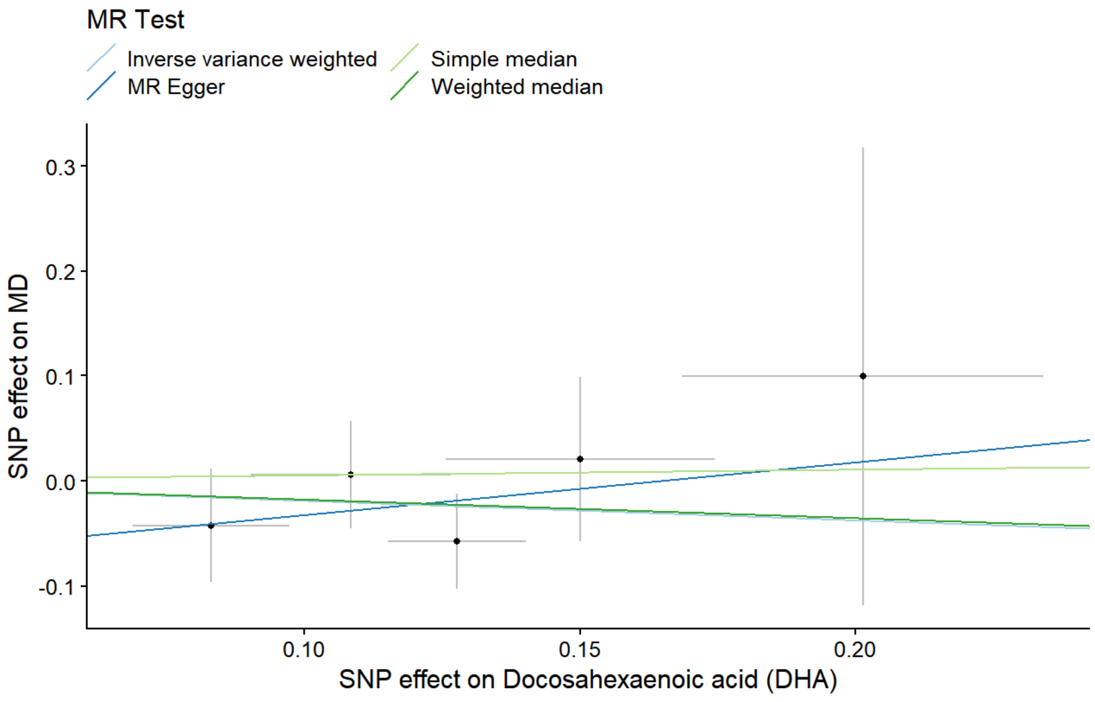

Figure S5.2 Scatterplot of docosahexaenoic acid (DHA) and MD

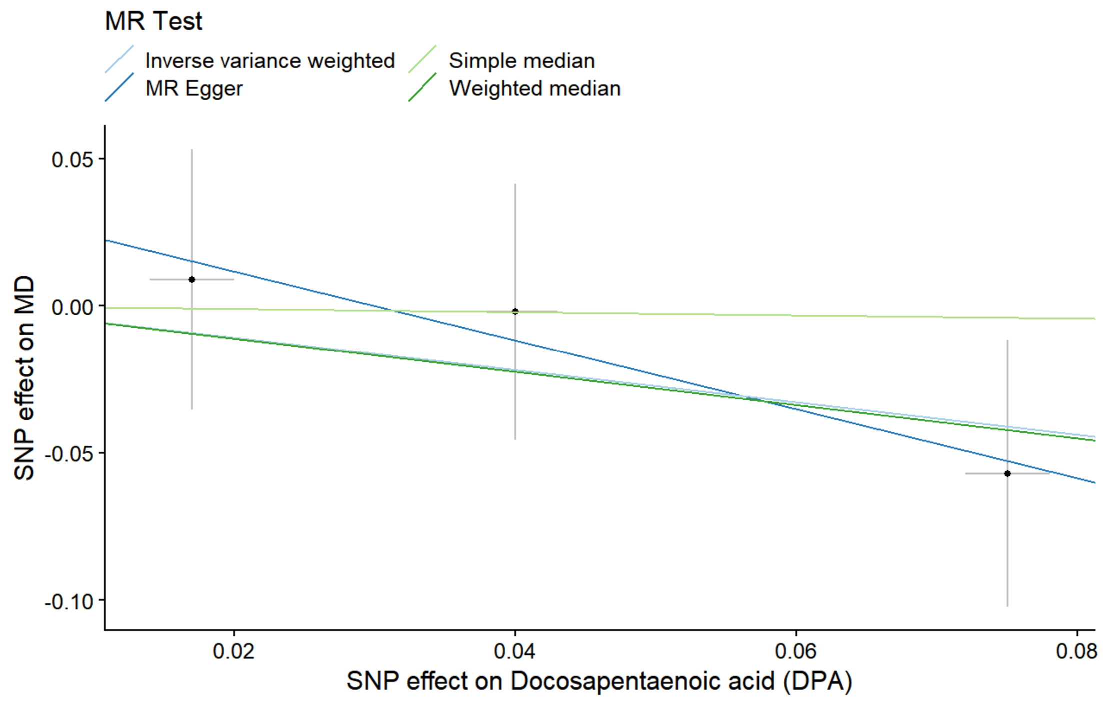

Figure S5.3 Scatterplot of docosapentaenoic acid (DPA) and MD

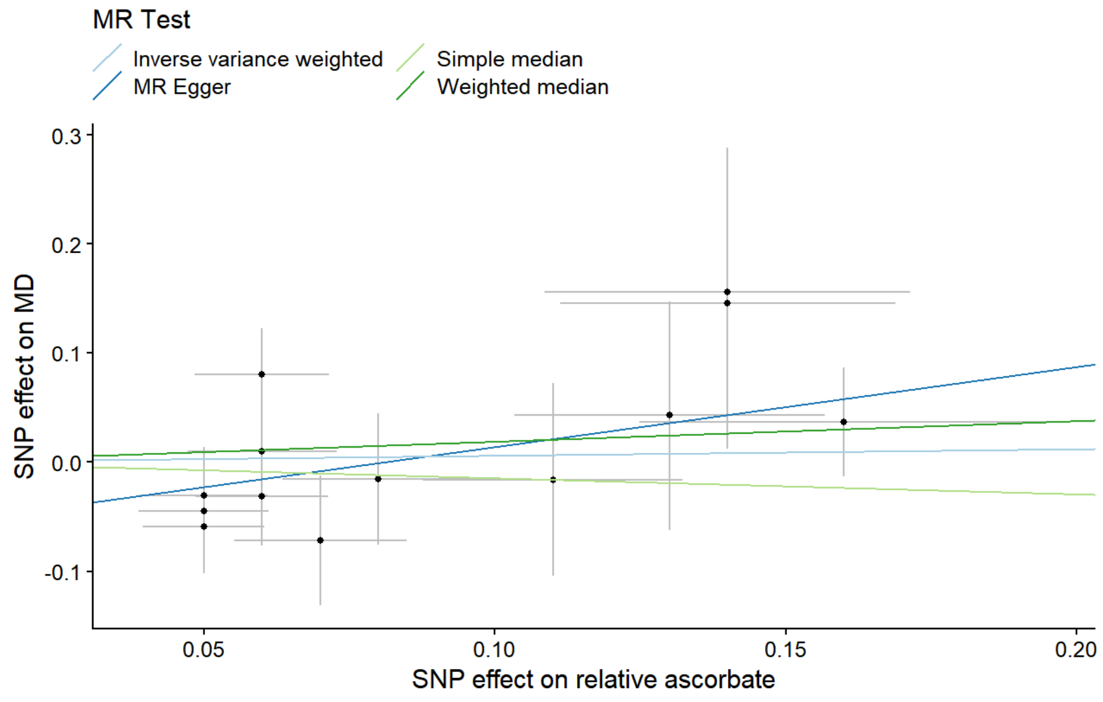

Figure S5.4 Scatterplot of relative ascorbate and MD

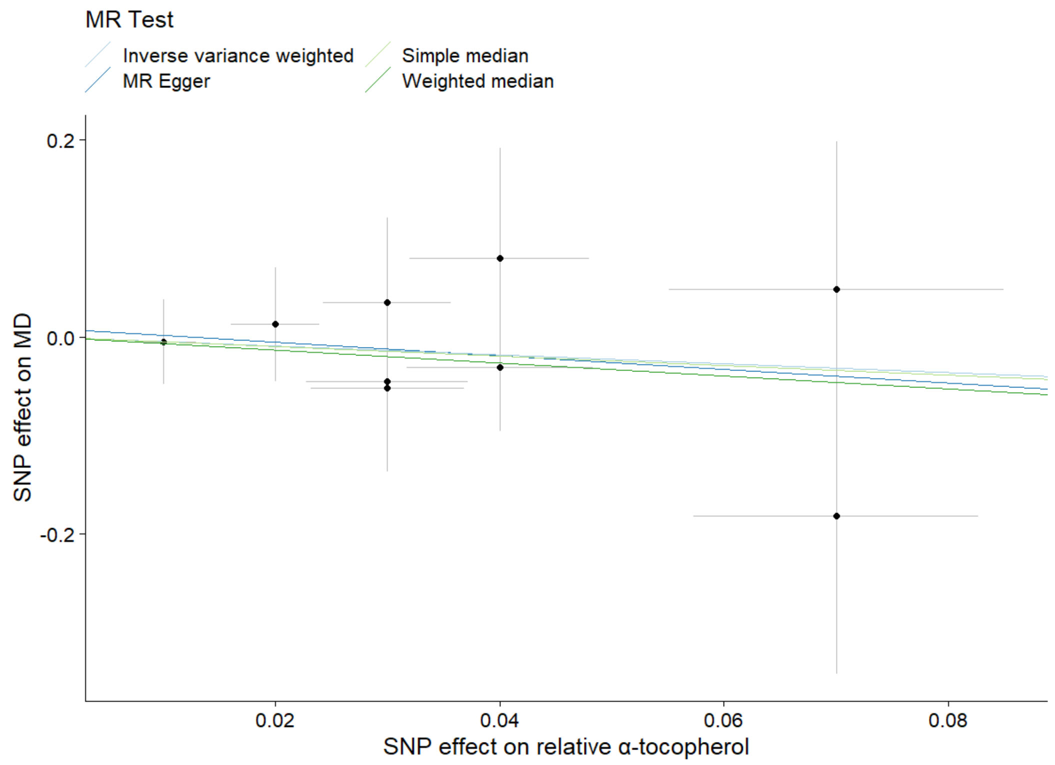

Figure S5.5 Scatterplot of relative α -tocopherol and MD

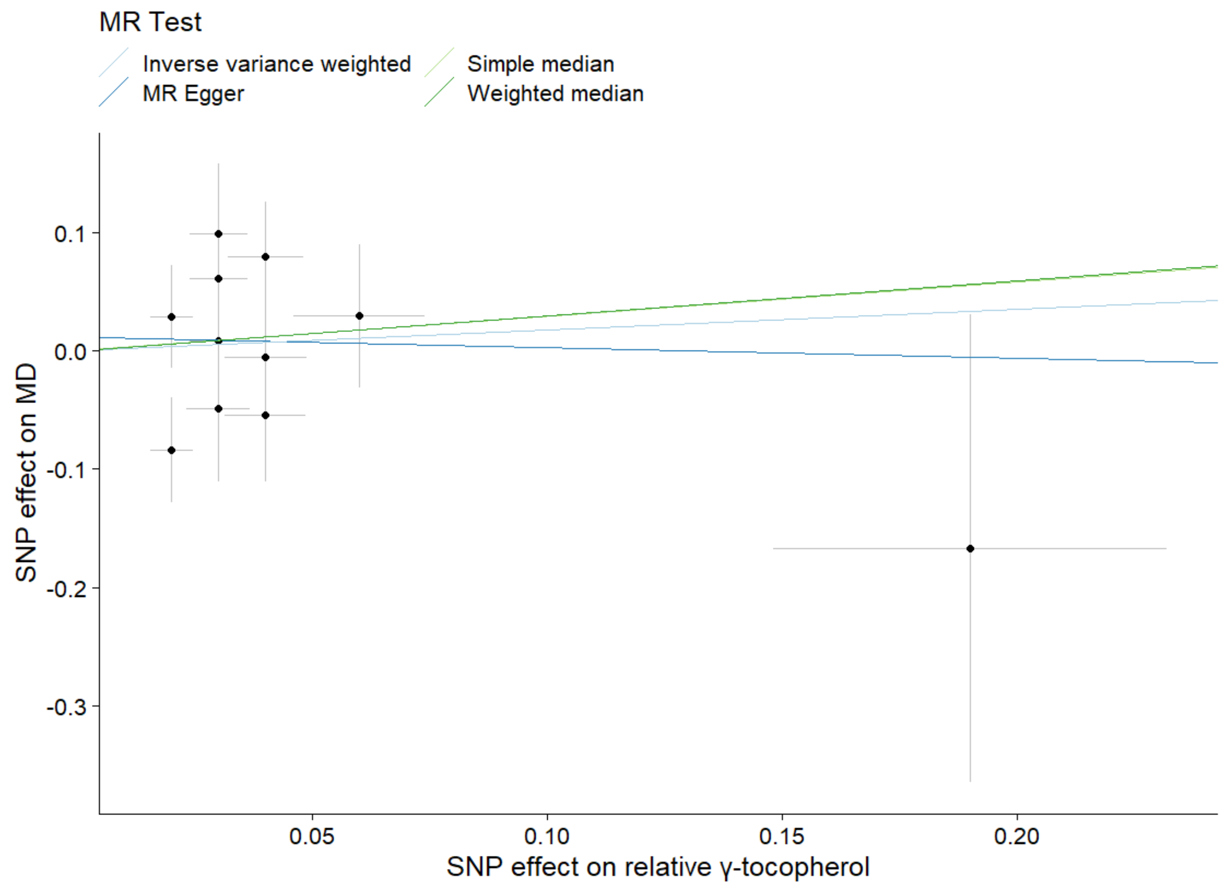

Figure S5.6 Scatterplot of relative γ -tocopherol and MD

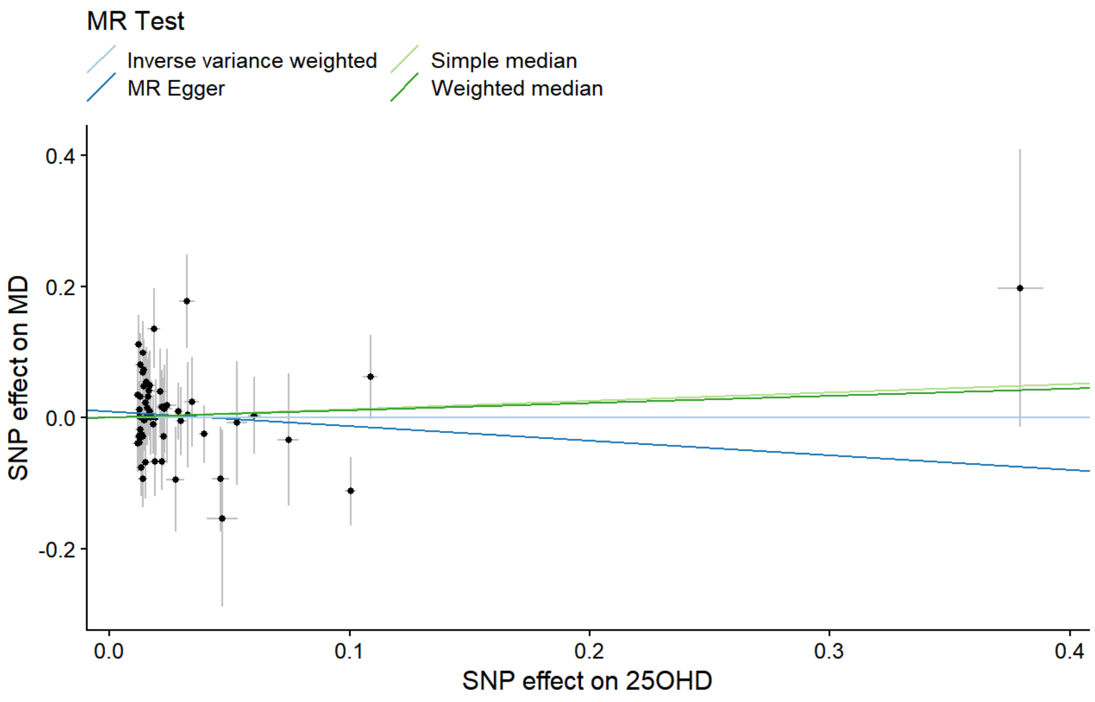

Figure S5.7 Scatterplot of 25OHD and MD

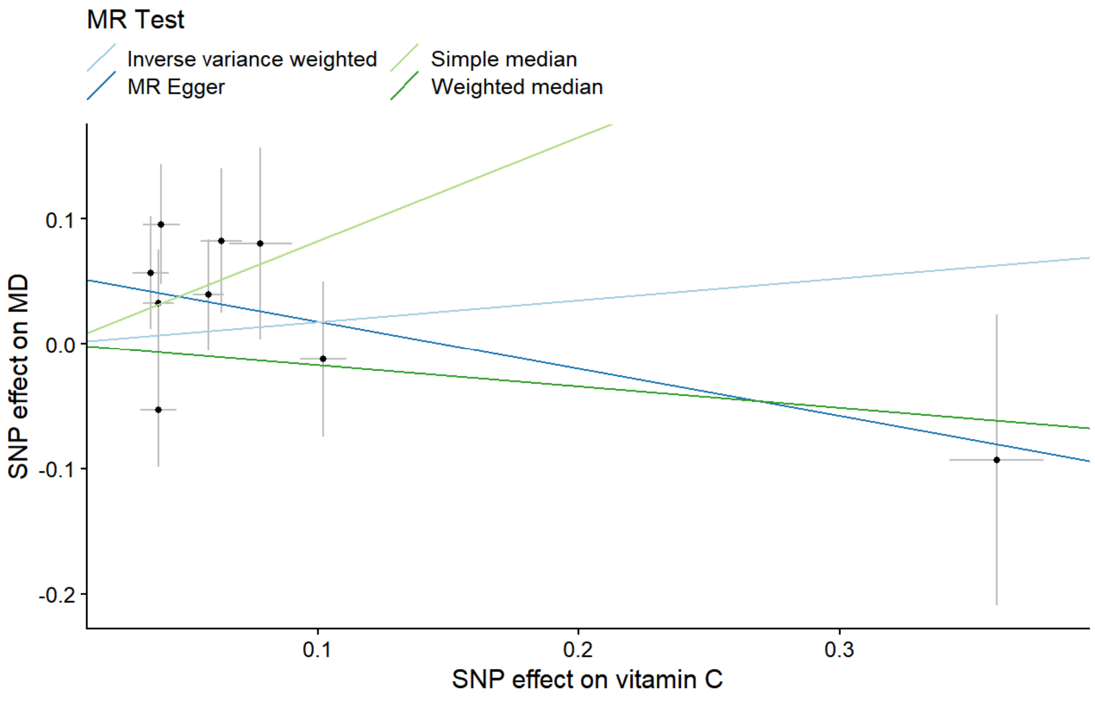

Figure S5.8 Scatterplot of vitamin C and MD

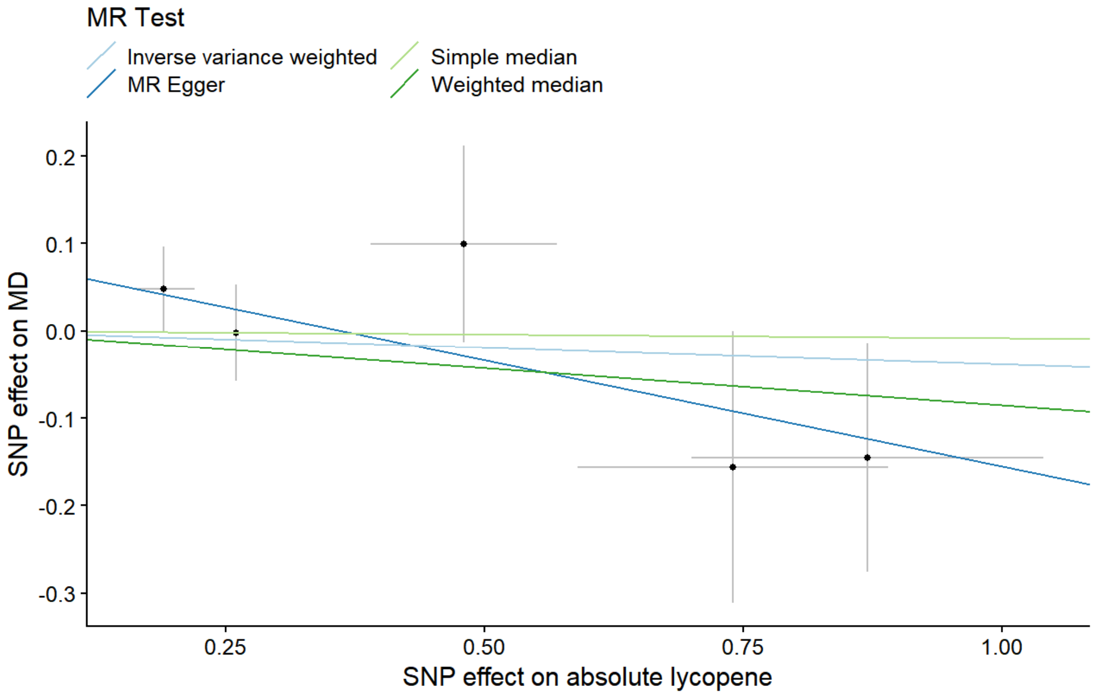

Figure S5.9 Scatterplot of lycopene and MD

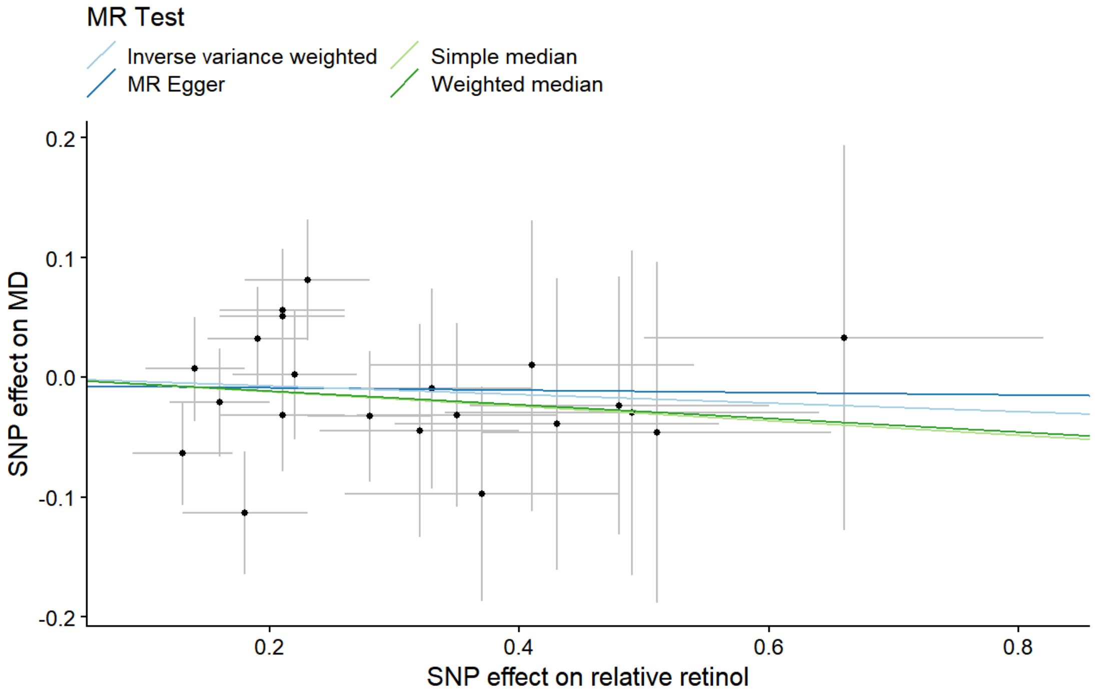

Figure S5.10 Scatterplot of relative retinol and MD

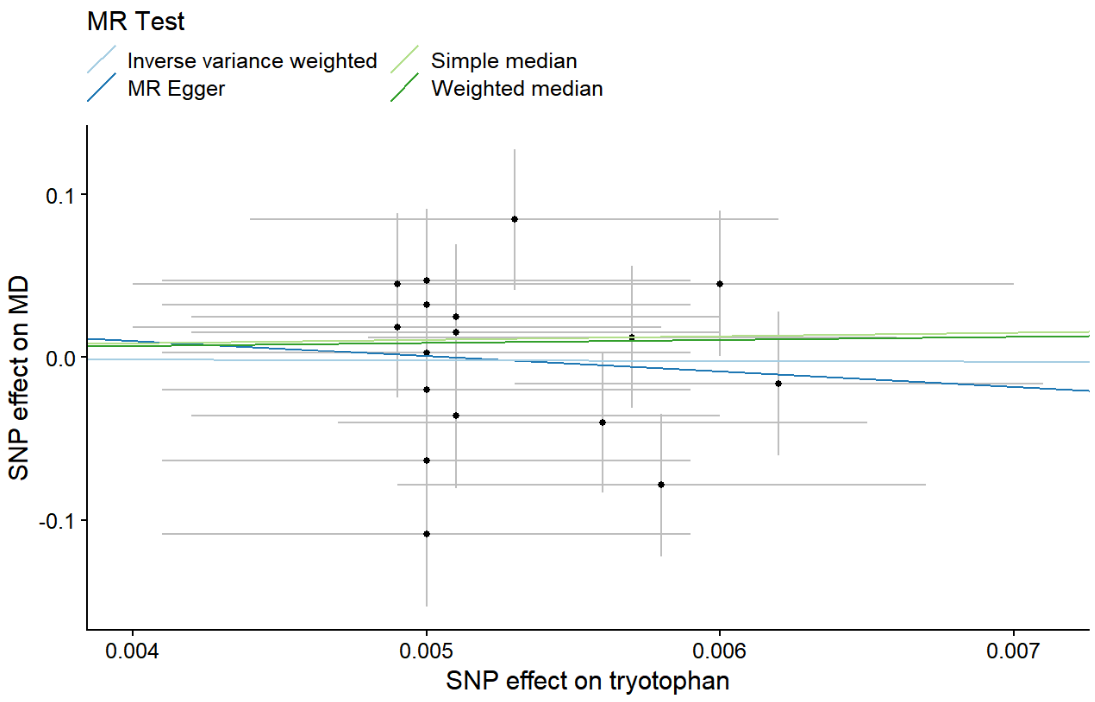

Figure S5.11 Scatterplot of tryptophan and MD

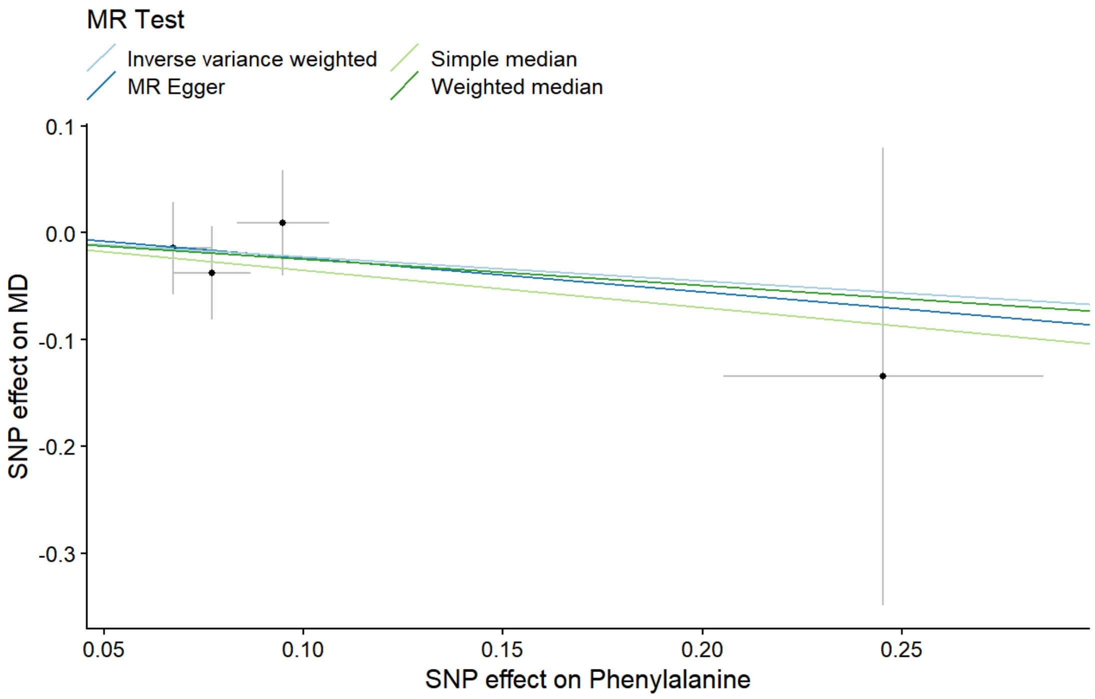

Figure S5.12 Scatterplot of phenylalanine and MD

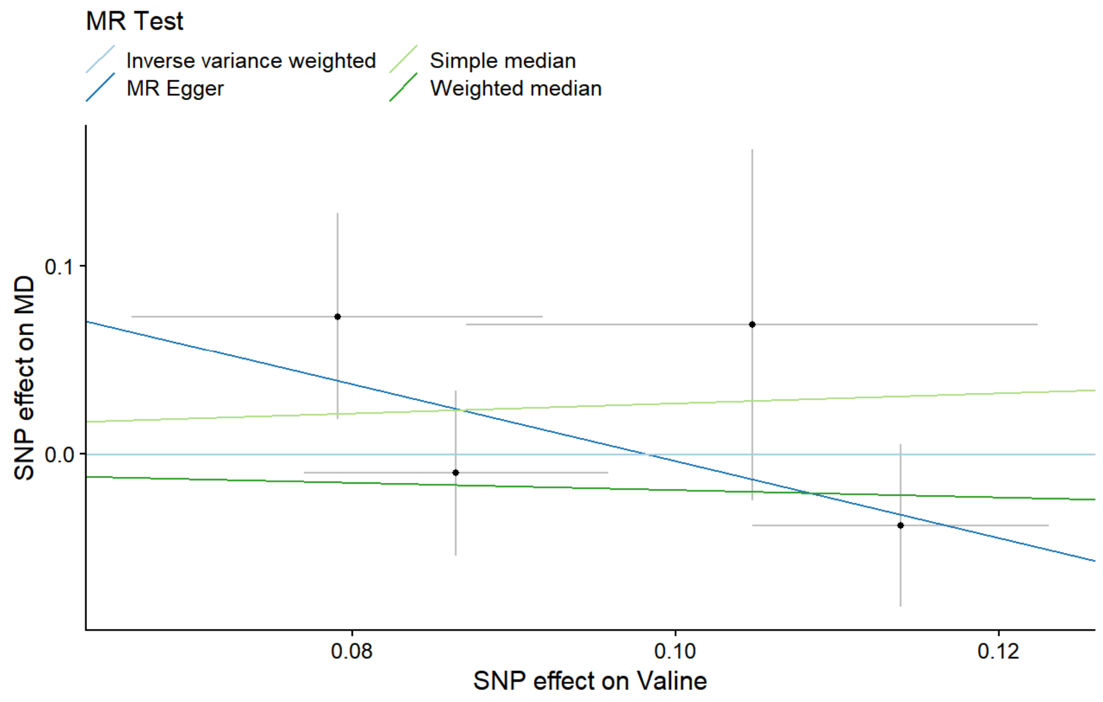

Figure S5.13 Scatterplot of valine and MD

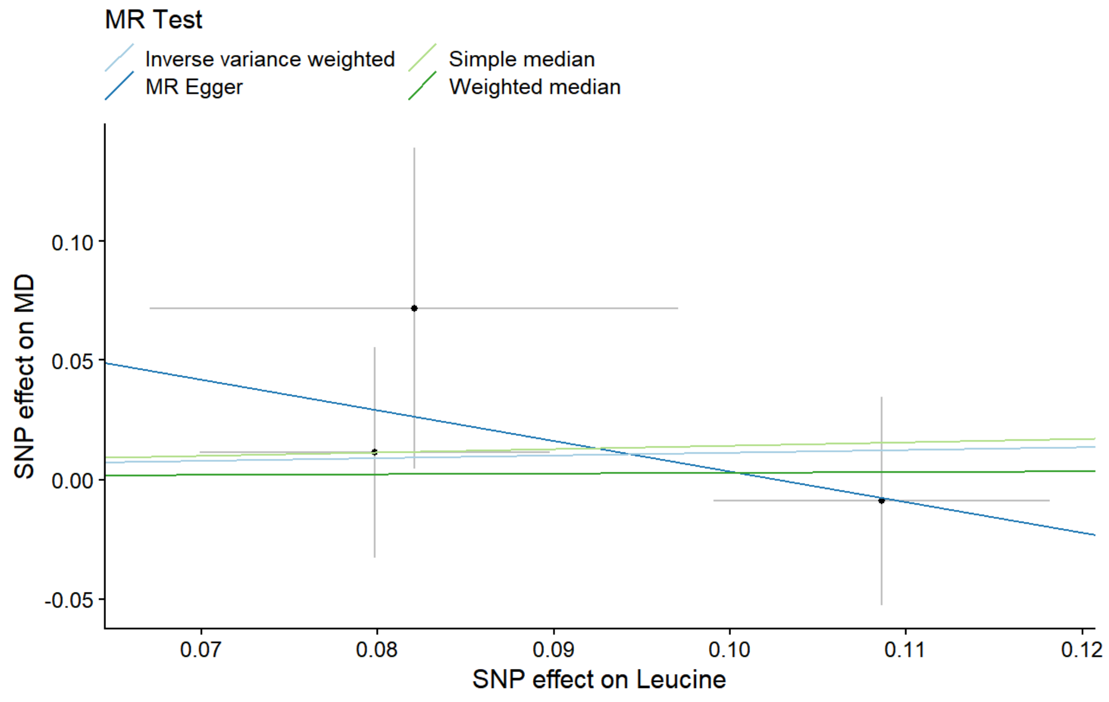

Figure S5.14 Scatterplot of leucine and MD

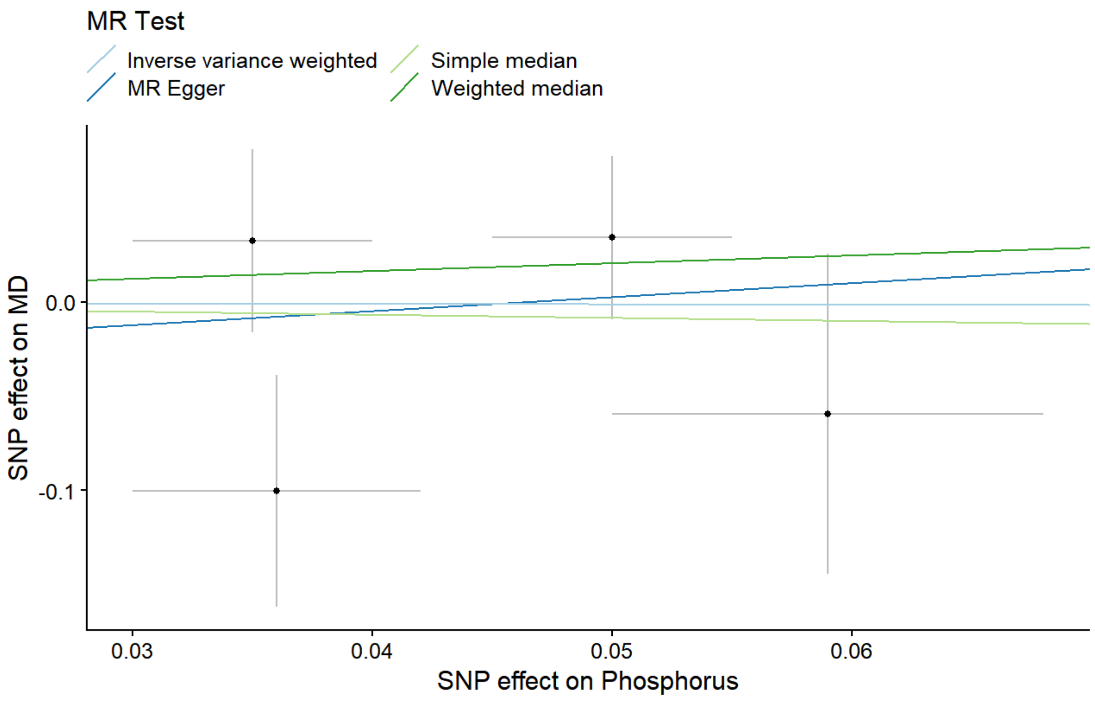

Figure S5.15 Scatterplot of phosphorus and MD

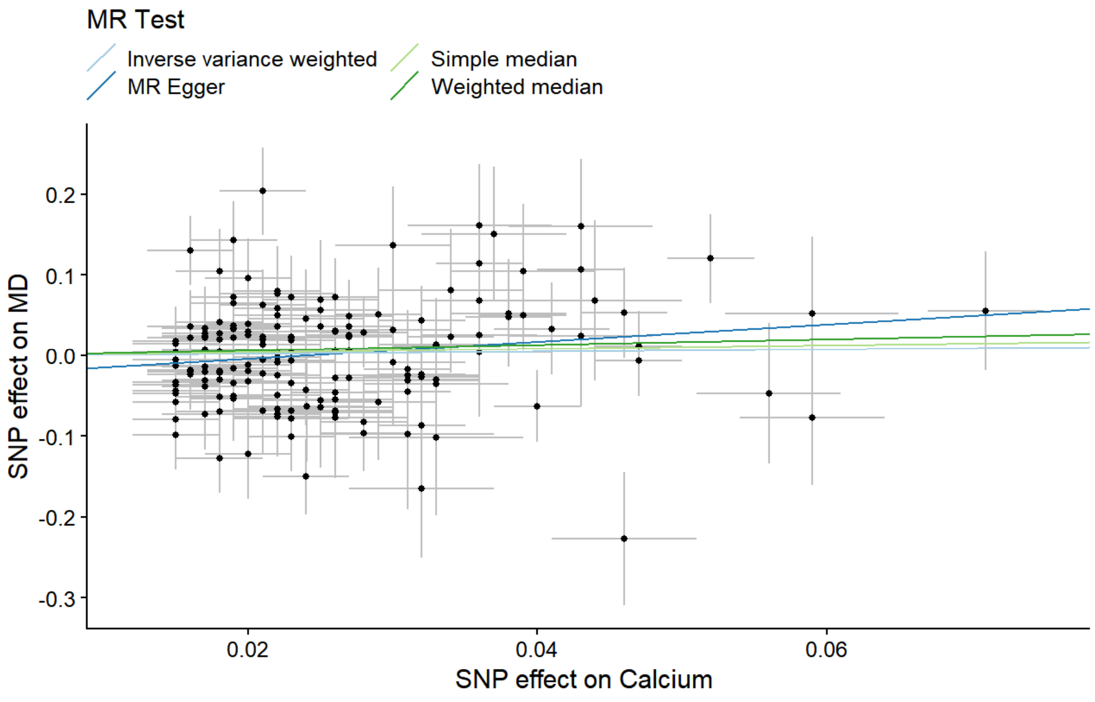

Figure S5.16 Scatterplot of calcium and MD

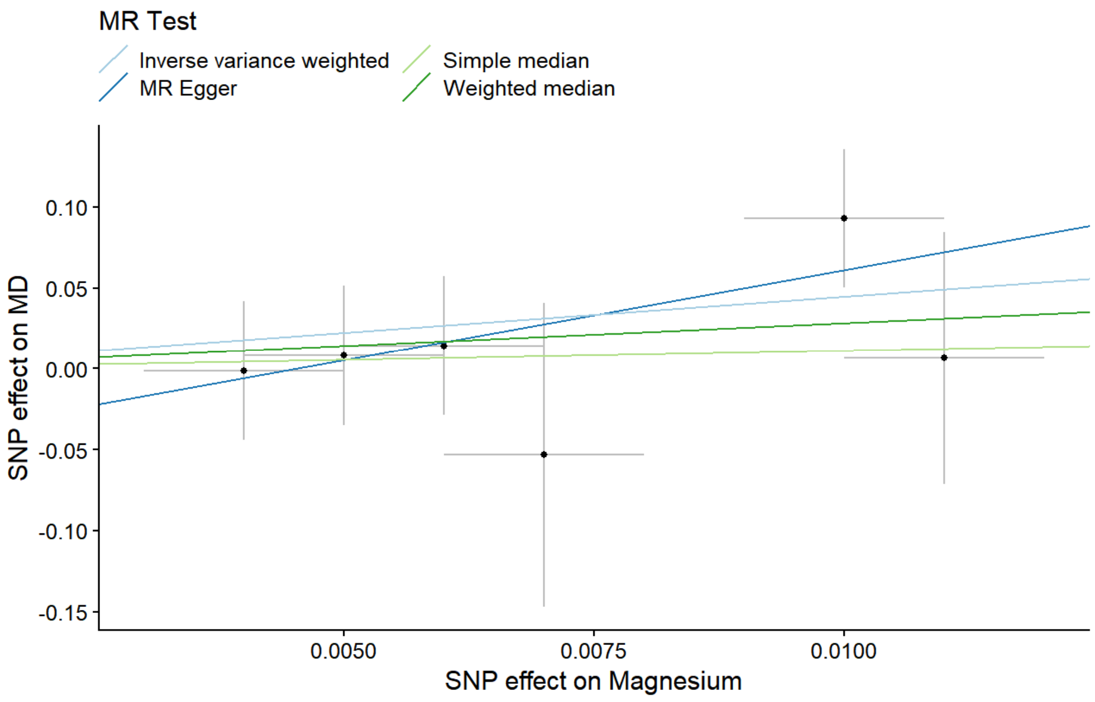

Figure S5.17 Scatterplot of magnesium and MD

Figure S5. Scatterplots of essential nutrients exposures and MD. The x-axis represents the previously published β-estimate for the association between each SNP and essential nutrients. The y-axis represents the β-estimate for the association between each SNP and risk of cerebral hemorrhage. The slope of each line corresponds to the estimated MR effect per method.

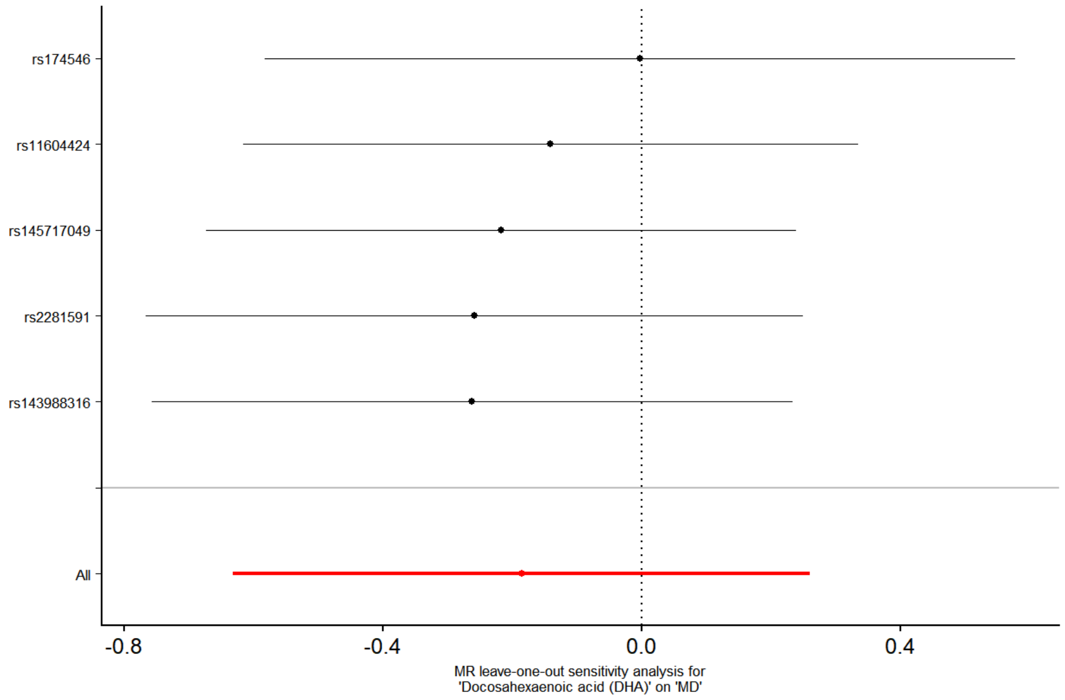

Figure S6.1 Leave-one-out plot of docosahexaenoic acid (DHA) and MD

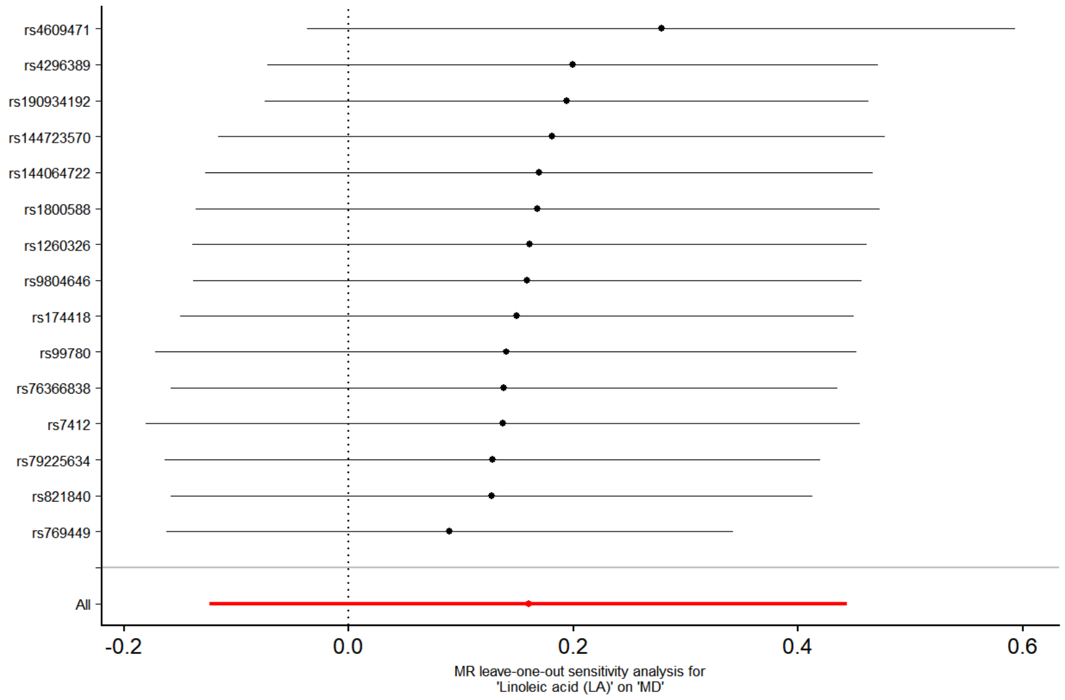

Figure S6.2 Leave-one-out plot of linolenic acid(LA) and MD

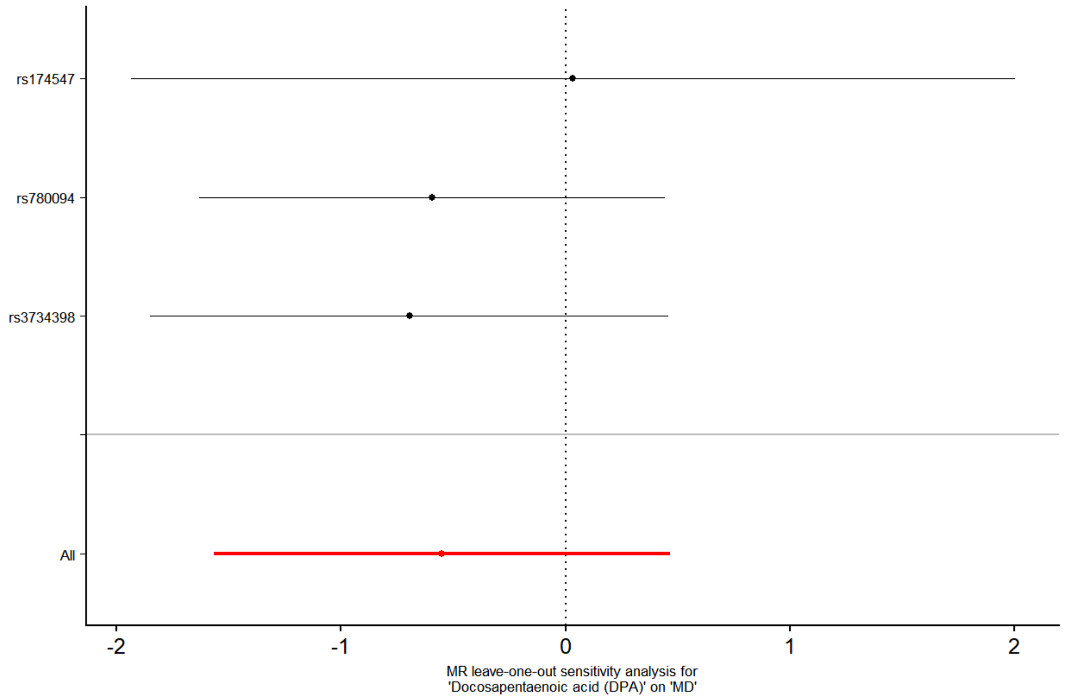

Figure S6.3 Leave-one-out plot of docosapentaenoic acid (DPA) and MD

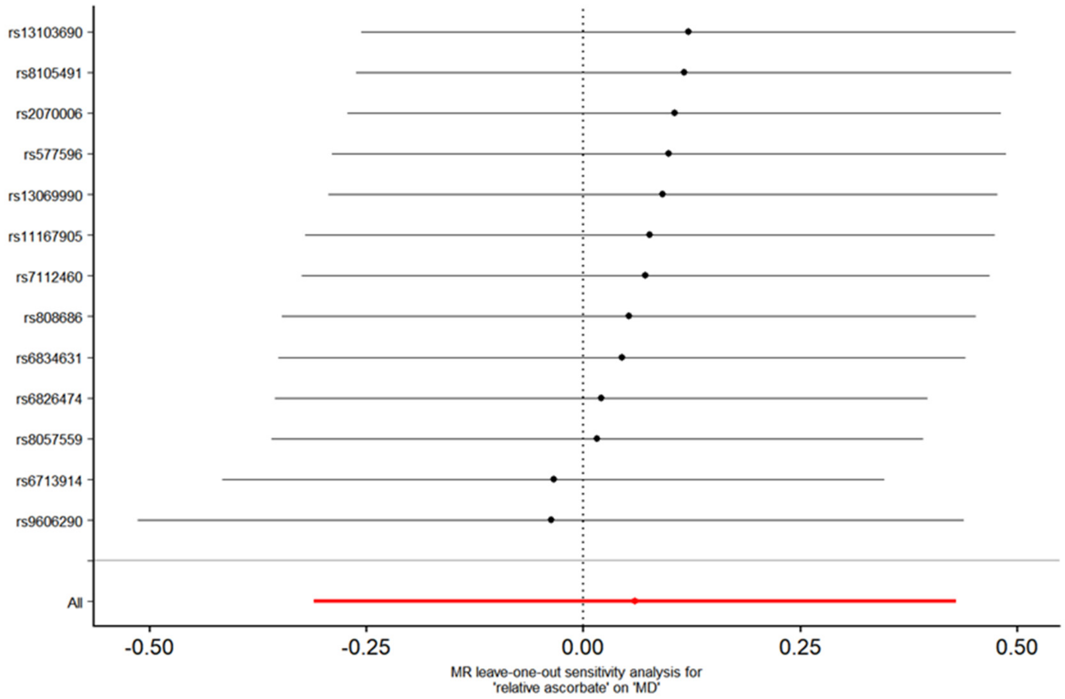

Figure S6.4 Leave-one-out plot of relative ascorbate and MD

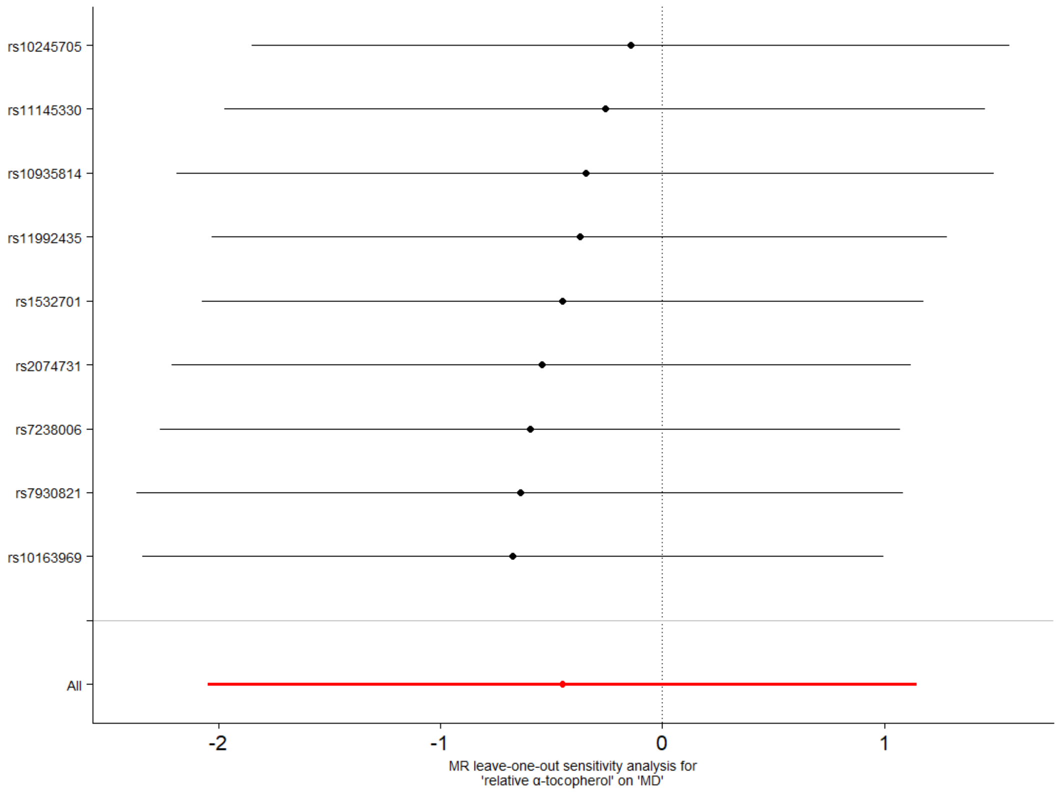

Figure S6.5 Leave-one-out plot of relative α -tocopherol and MD

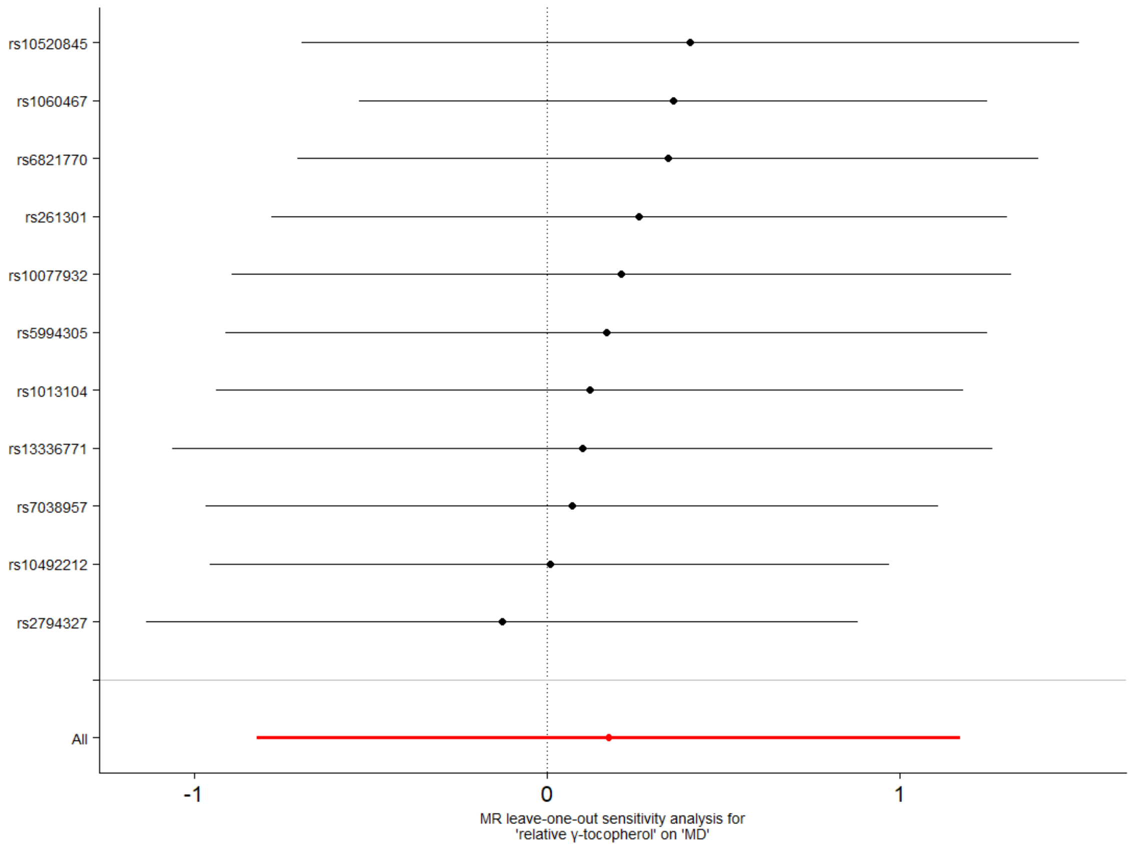

Figure S6.6 Leave-one-out plot of relative γ -tocopherol and MD

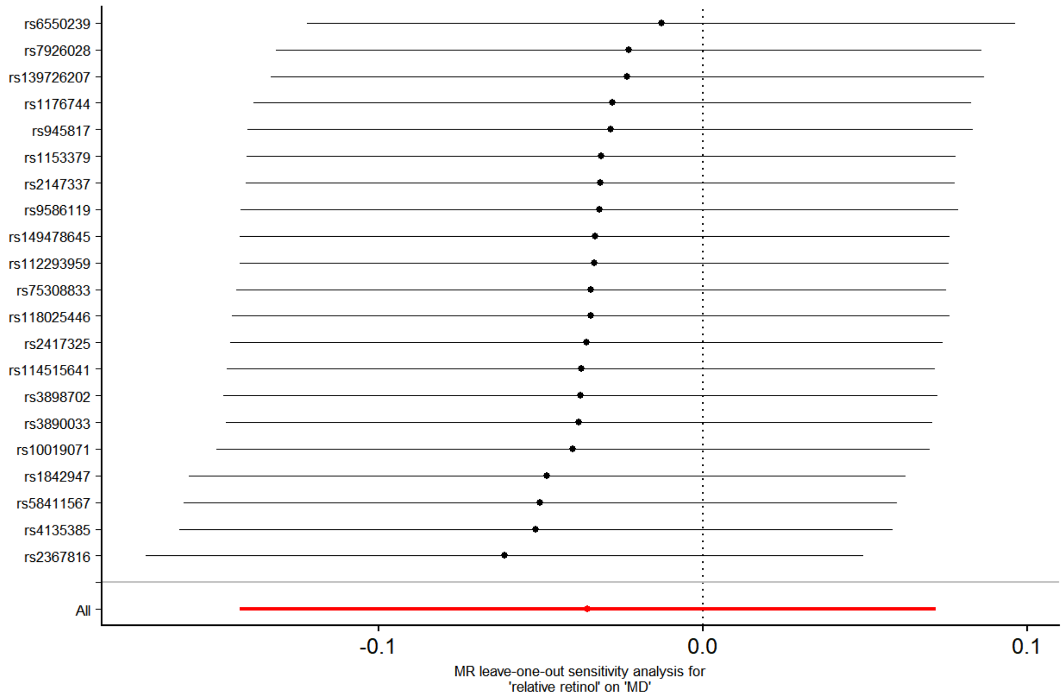

Figure S6.7 Leave-one-out plot of relative retinol and MD

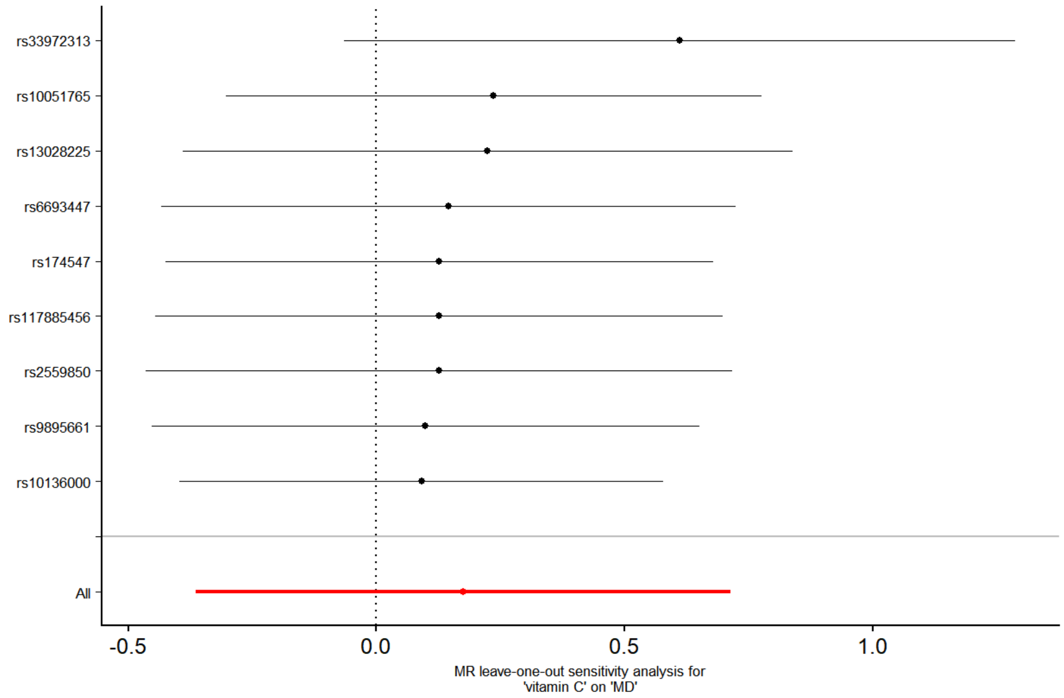

Figure S6.8 Leave-one-out plot of vitamin C and MD

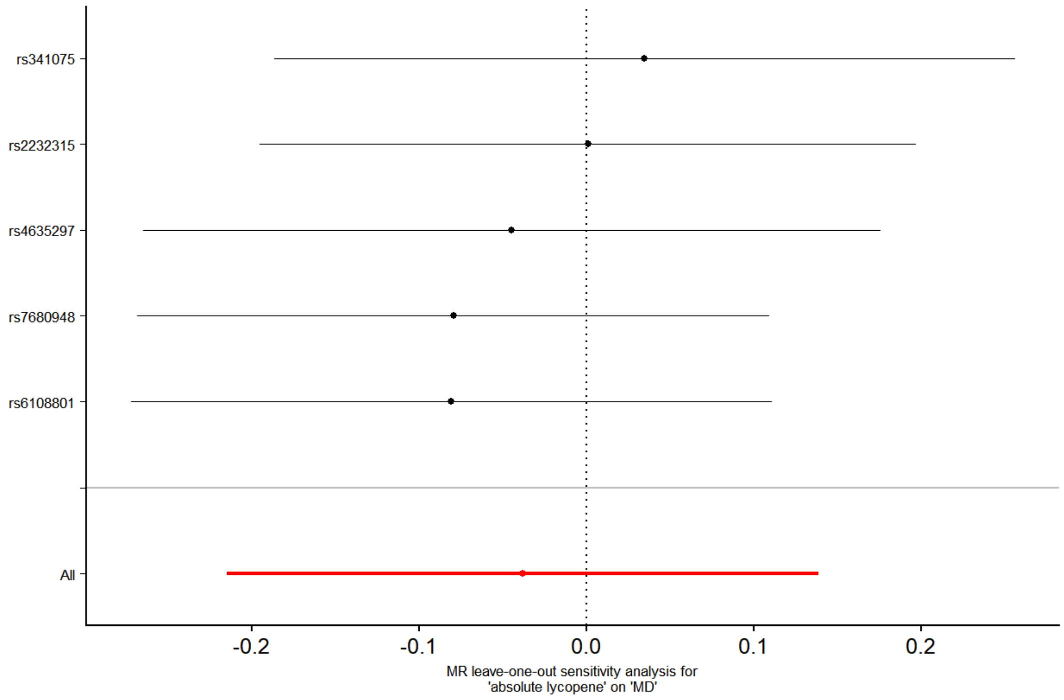

Figure S6.9 Leave-one-out plot of absolute lycopene and MD

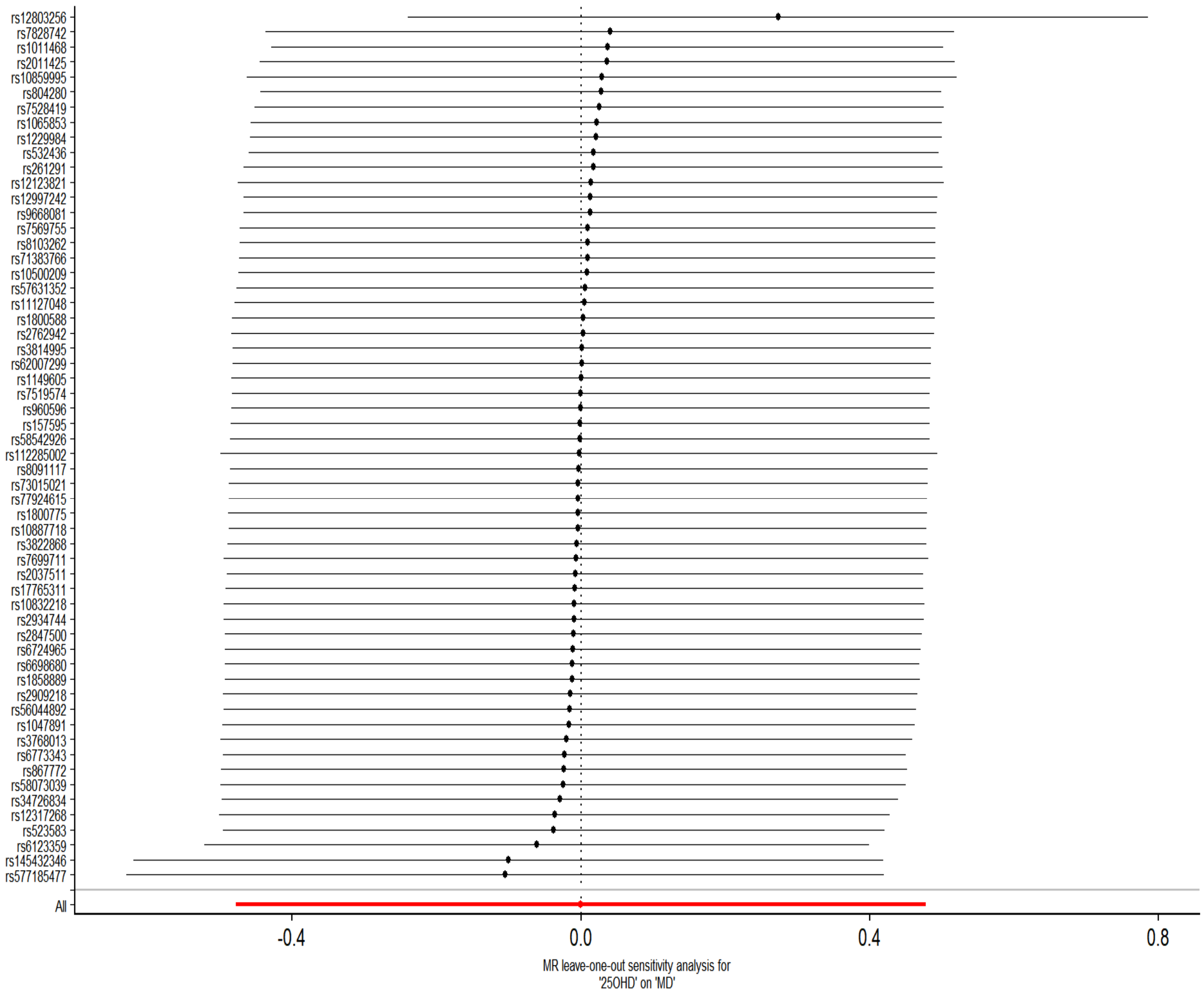

Figure S6.10 Leave-one-out plot of 25OHD and MD

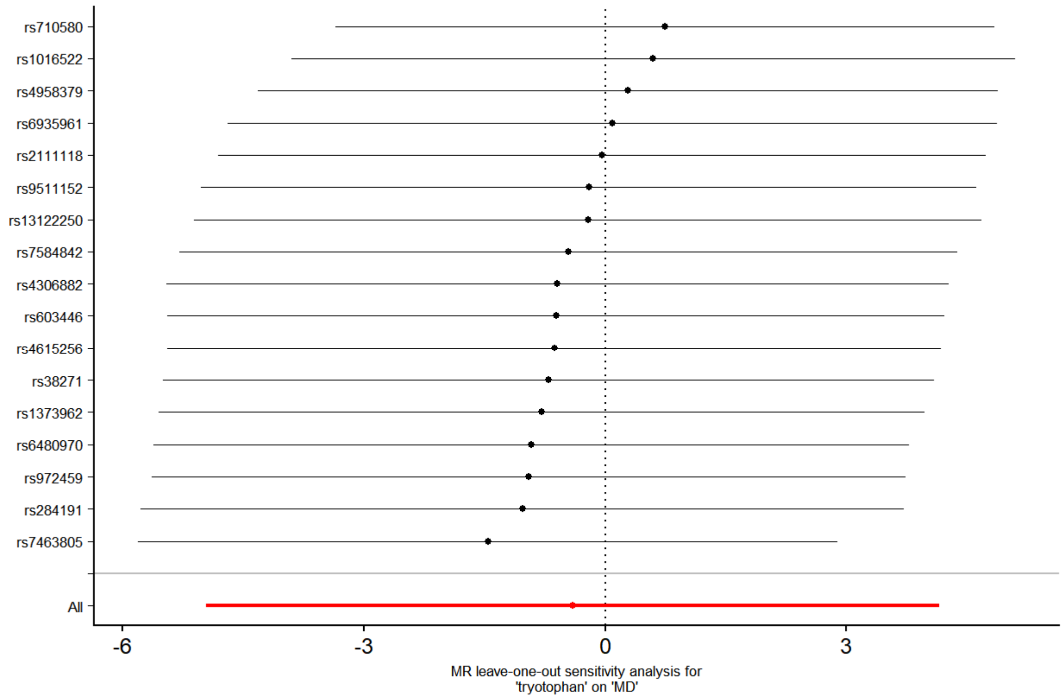

Figure S6.11 Leave-one-out plot of tryptophan and MD

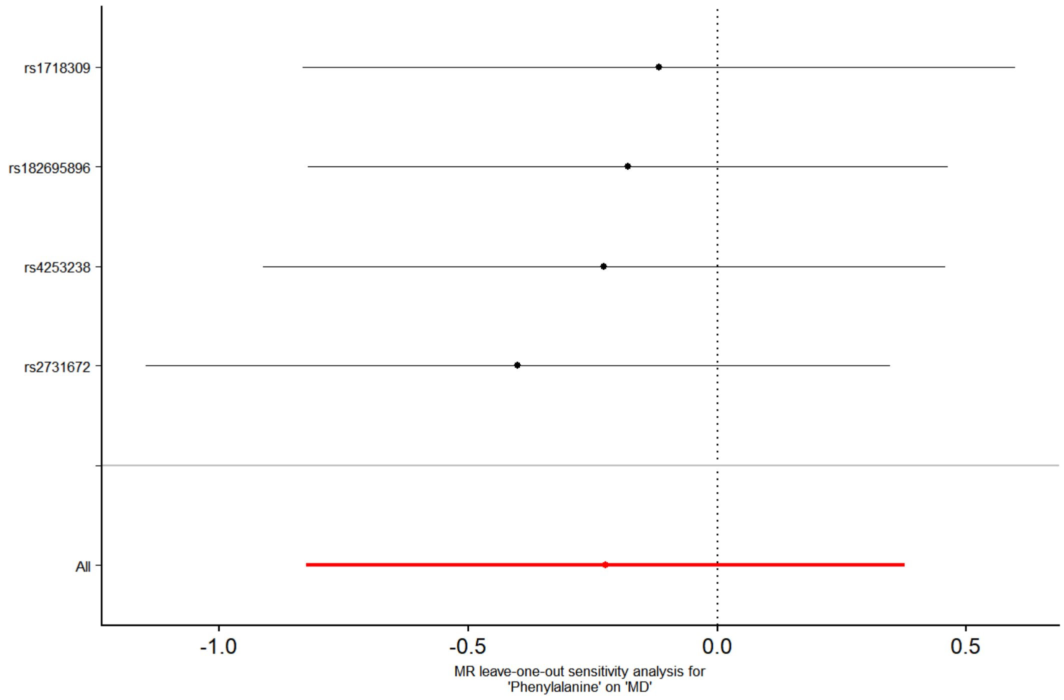

Figure S6.12 Leave-one-out plot of phenylalanine and MD

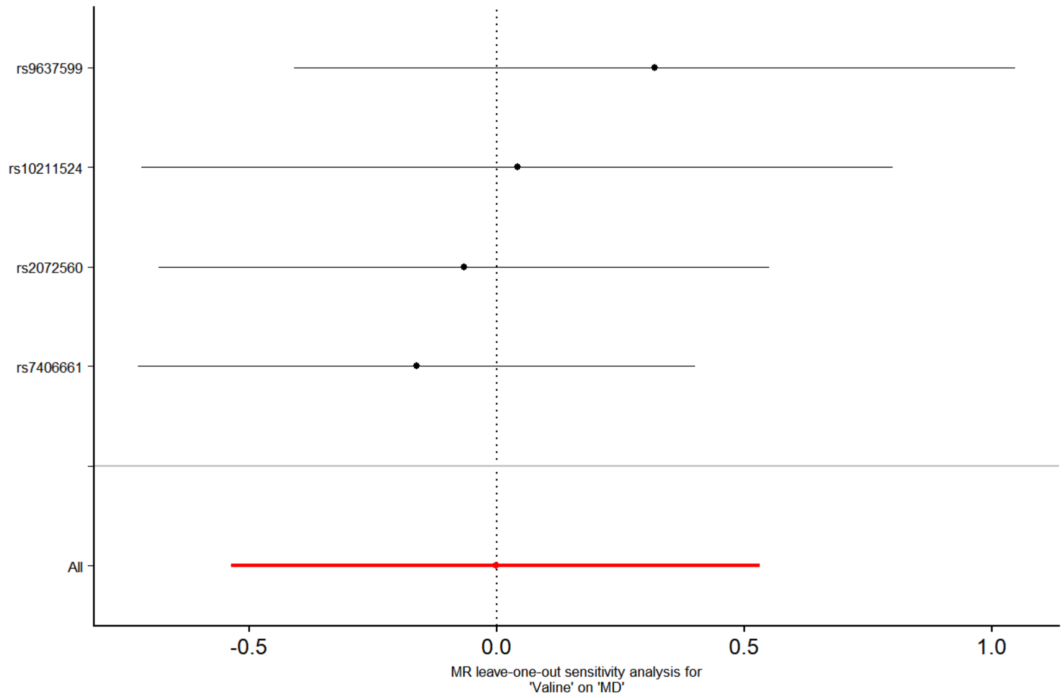

Figure S6.13 Leave-one-out plot of valine and MD

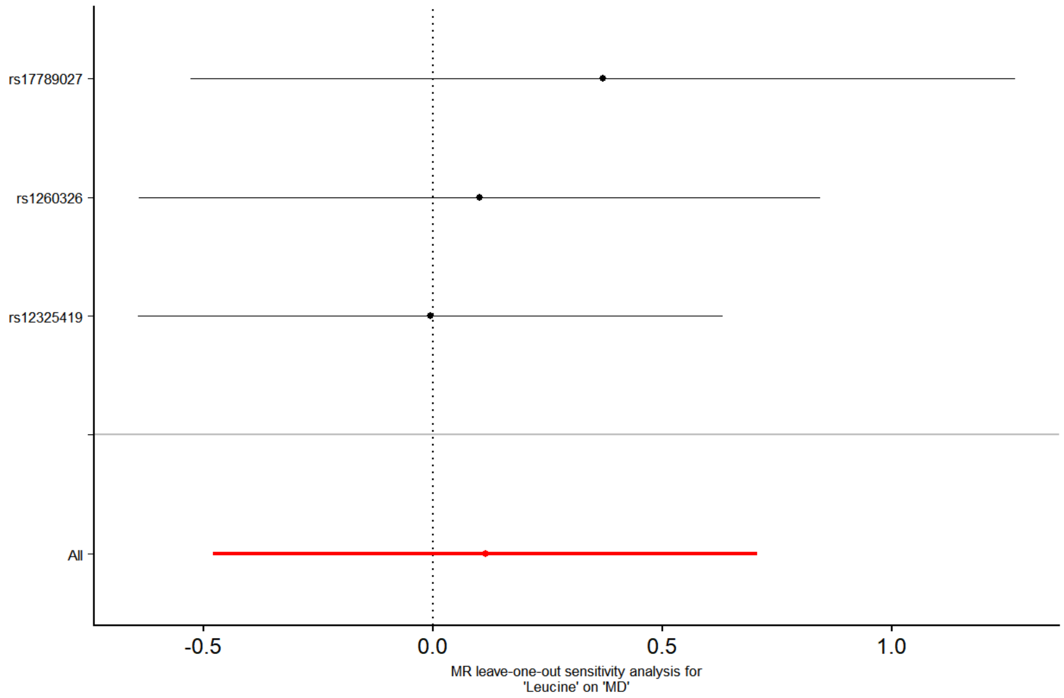

Figure S6.14 Leave-one-out plot of leucine and MD

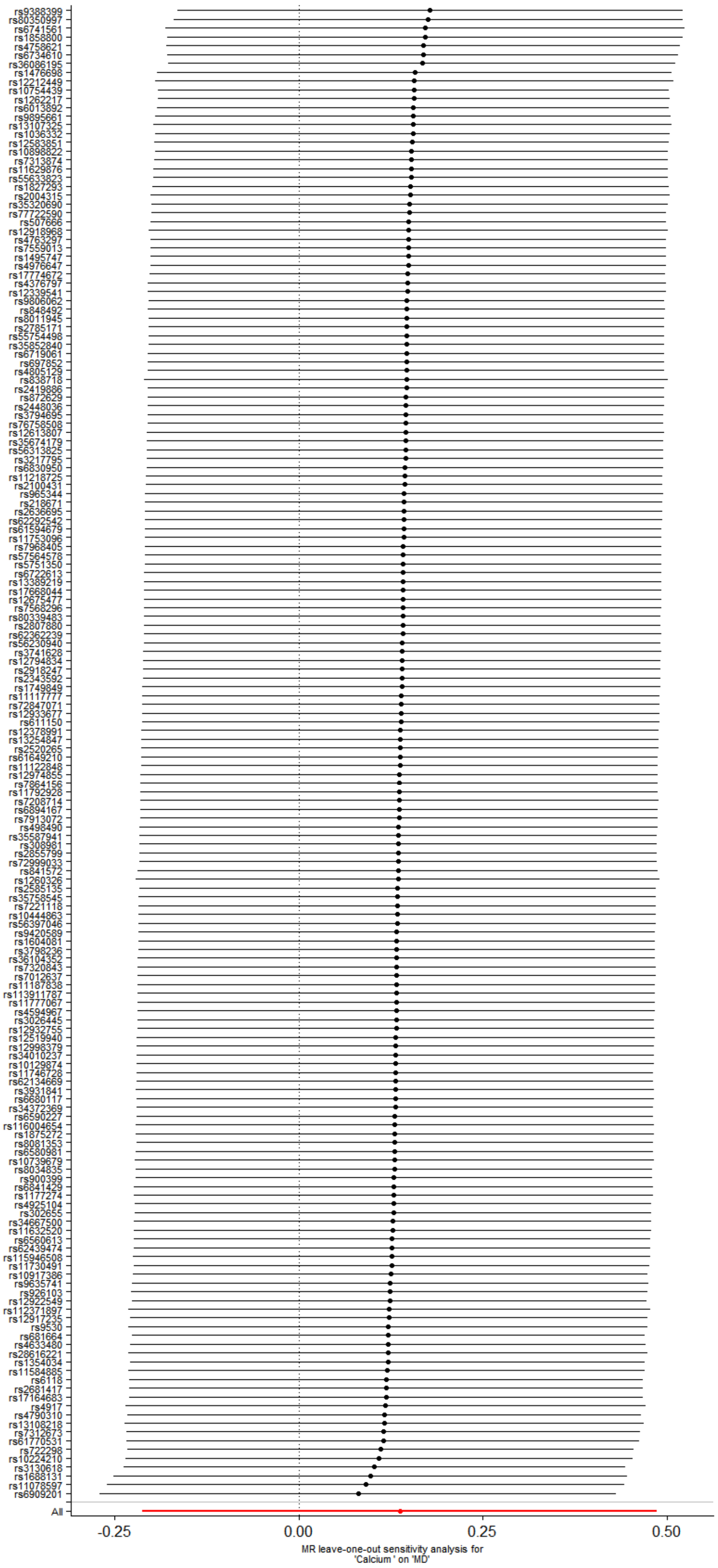

Figure S6.15 Leave-one-out plot of calcium and MD

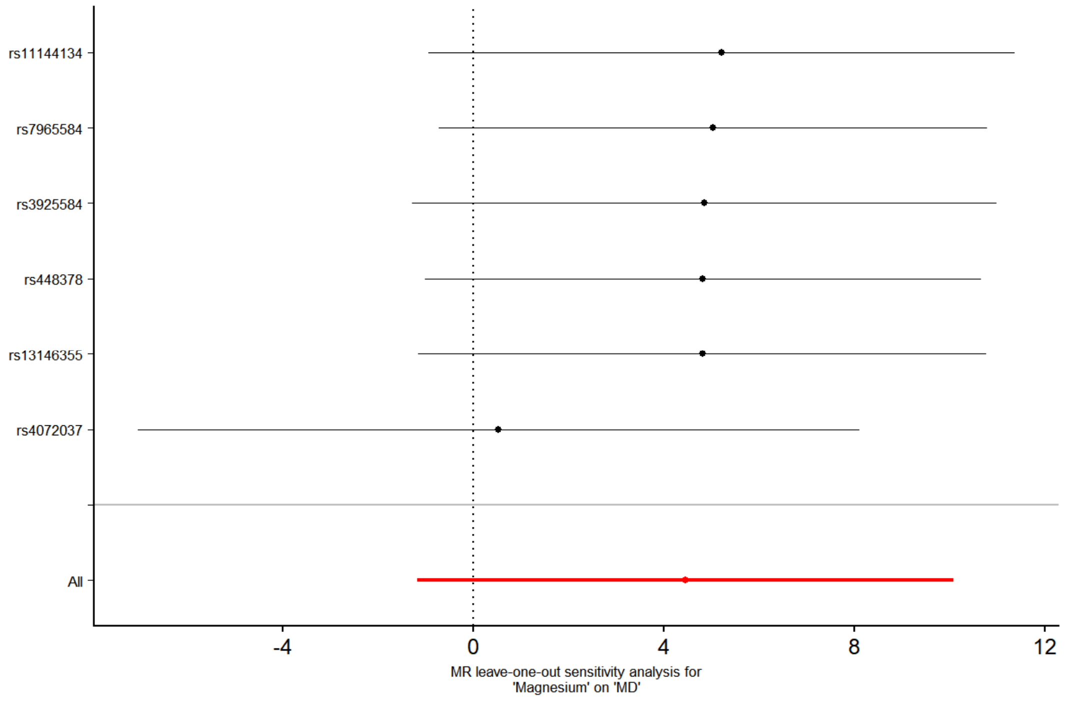

Figure S6.16 Leave-one-out plot of magnesium and MD

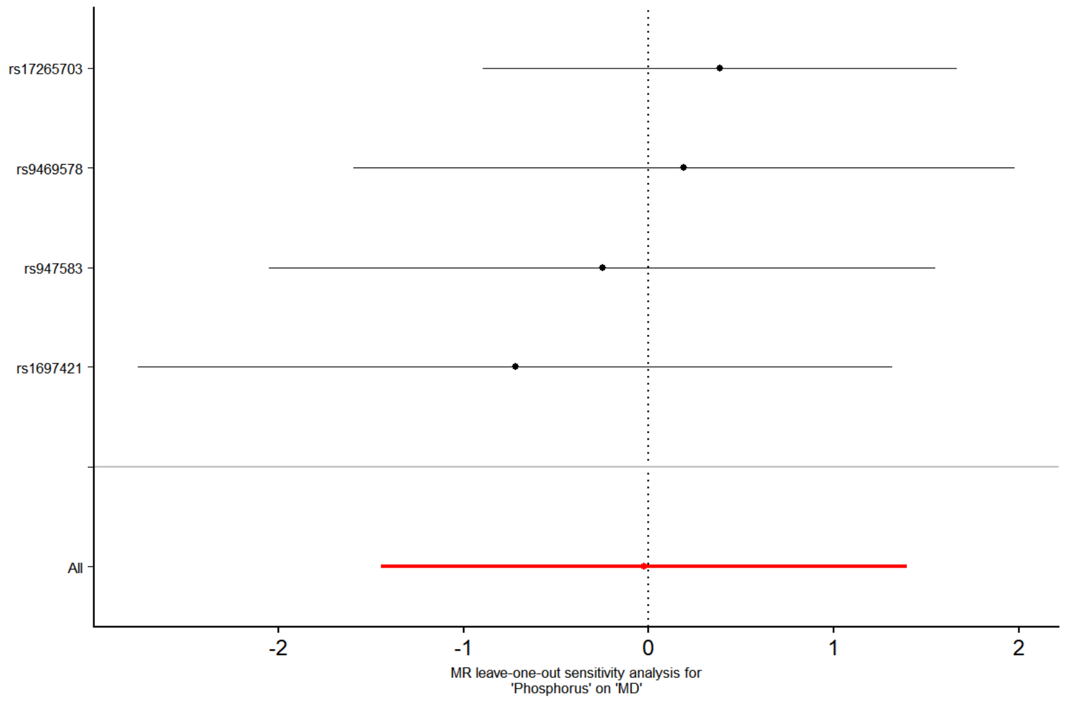

Figure S6.17 Leave-one-out plot of phosphorus and MD

Figure S6 Leave-one-out plots of essential nutrients exposures and MD. The x-axis represents the beta value for the outcome obtained by removing the left SNP from the IVW analysis (i.e., the dots on each solid line). The y-axis represents the SNP removed for each analysis. Each solid line represents the 95% CI for the beta value. The bottom red line is the overall result obtained by all the SNPs of exposure.
